# [3 + 2] Cycloadditions of Tertiary Amine *N*-Oxides and Silyl Imines as an Innovative Route to 1,2-Diamines

**DOI:** 10.1021/acs.orglett.3c01396

**Published:** 2023-06-15

**Authors:** Sarah L. Hejnosz, Danielle R. Beres, Alexander H. Cocolas, Martin J. Neal, Benjamin S. Musiak, Marianne M. B. Hanna, Aaron J. Bloomfield, Thomas D. Montgomery

**Affiliations:** Department of Chemistry and Biochemistry, Duquesne University, 600 Forbes Avenue, Pittsburgh, Pennsylvania 15282, United States

## Abstract

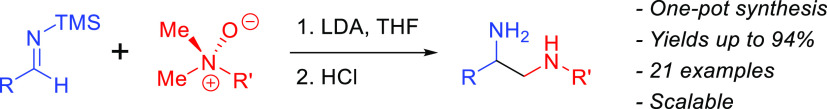

We have developed a one-pot synthetic method for producing 1,2-diamines from easily prepared and commercially available precursors through a formal umpolung process. Our method utilizes an efficient [3 + 2] cycloaddition as the key step in forming substituted 1,2-diamines in moderate to high yields. These resulting compounds can undergo subsequent transformations, demonstrating their utility as synthetic building blocks for more complex scaffolds. Finally, we propose a reasonable mechanism for this transformation using density functional theory modeling, justifying the experimental observations.

1,2-Diamines are privileged structures found in an array of bioactive compounds, such as natural products and pharmaceuticals.^1^ They are useful building blocks for complex synthetic targets as a result of the myriad of transformations that they can undergo,^1a,1b,2^ and they are commonly employed as ligands for transition-metal-catalyzed processes.^3^ Given the versatility of this motif, more efficient methods for synthesizing this scaffold from base materials are highly sought.

There are numerous syntheses of 1,2-diamines in the literature, but the search for new and efficient methods to make these privileged motifs is still ongoing.^2a,4^ Most synthetic pathways focus on producing the diamine through C–N bond formations (Scheme 1).^5^ Recent examples include work by Hull and co-workers, where they demonstrated a rhodium-catalyzed directed hydroamination forming a broad scope of 1,2-diamines through C–N disconnection **A** (Scheme 1).^6^ Wen and co-workers effectively coupled disconnections **A** and **C** through a base-promoted diamination of styrene sulfonium salts (Scheme 1).^7^ Their method tolerated a good scope of sulfonium salts and amines, but it is limited to chemically indistinct arylamines.^7^ While these approaches are effective, some of the synthetic precursors or needed ligands are not commercially available, requiring more time and energy.^6,7^ Additionally, these transformations, like many others, result only in the installation of amines, while the carbon backbone remains unchanged throughout the scope (disconnections **A** and **C** in Scheme 1). When this is not the case, such as with aza-Henry^8^ and Strecker^9^ reactions, harsh conditions and several steps are required to form the desired diamines. Alemán and co-workers recently advanced the field through their acid-mediated imidazolidine formation, which can be converted into a functionalized diamine in two steps.^10^ Here, we report a [3 + 2] cycloaddition between silyl imines generated *in situ* and an *N*-oxide forming a wide range of 1,2-diamines through a C–C bond-forming reaction.

**Scheme 1:**
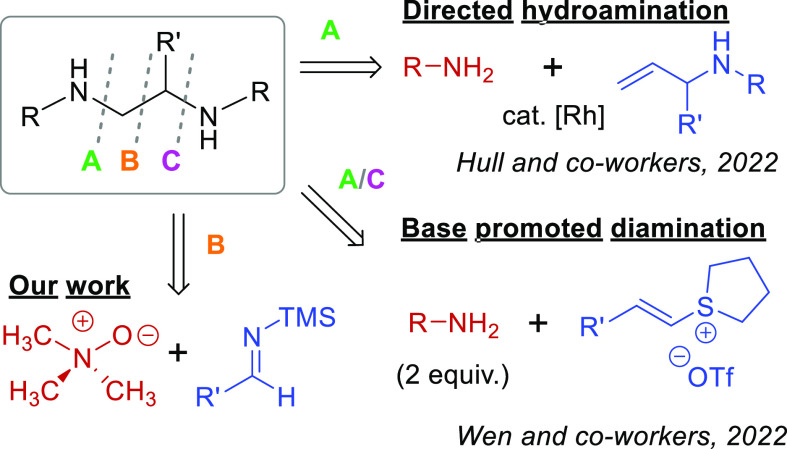
Synthetic Methods Forming 1,2-Diamines Highlighting Common Disconnections

1,3-Dipolar cycloadditions using azomethine ylides have been well-studied in the literature^11^ and have seen significant utility in the synthesis of heterocycles.^12^ Many tactics have been employed for generating azomethine ylides.^13^ While effective, these approaches typically require multiple steps and the incorporation of anion-stabilizing groups. A direct route to non-stabilized azomethine ylides was pioneered by Roussi and co-workers in the 1980s and involves conversion of tertiary amine *N*-oxides to azomethine ylides for [3 + 2] cycloaddition reactions.^14^ Given the efficiency of this route, this chemistry has been relatively underused and underdeveloped in the following years, with only a handful of more recent examples appearing in the literature.^15^

Chastanet and Roussi’s original reports were limited to aryl alkenes, with styrene and *trans*-stilbene accounting for the majority of reported examples.^14d^ More recent developments were made by Davoren et al.^15a^ and Bao and Liu,^15b^ focusing on greater tolerance for *trans*-stilbene derivatives. Similarly, Mirzayans and co-workers’ 2014 paper discussed using *N*-methylmorpholine *N*-oxide (NMO) as a 1,3-dipolar cycloaddition precursor.^15c^ However, apart from two specific examples from Roussi and co-workers’ 1987 report, all work in this area has involved using olefins as dipolarophiles.^14b^ Liu and co-workers were recently able to synthesize similar structures via a 1,3-dipolar cycloaddition reaction starting from an aziridine and a formyl imine precursor, which allows for late-stage functionalization with the tosyl-protected amine.^12a^ Given the ubiquity of azomethine ylide [3 + 2] cycloadditions and the versatility of the *N*-oxides as precursors, we felt that this area deserved to be more deeply explored.

We recently reported that a tertiary amine *N*-oxide forms an azomethine ylide through a multi-ion-bridged intermediate, as opposed to a discrete iminium intermediate.^16^ Our proposed reaction pathway strongly suggested that this transformation should have a greater synthetic utility than had been previously reported. We explored the reaction between tertiary amine *N*-oxides and silyl-protected imines **2** (Scheme 2). Silyl-protected imine **2** was formed via the addition of sodium hexamethyldisilazide (NaHMDS) to aldehyde **1**, with the resulting hemiaminal undergoing an aza-Brook rearrangement.^17^ Compound **2** was then combined with *N*-oxide and lithium diisopropylamide (LDA) to undergo the [3 + 2] cycloaddition reaction, forming the imidazolidine intermediate **3**. Imidazolidine **3** was hydrolytically unstable, presumably as a result of the weak N–Si bond. We elected to trigger ring opening to form substituted 1,2-diamine **4** through the addition of hydrochloric acid and hydroxylamine hydrochloride, with the latter serving as a formaldehyde scavenger.^18^ We optimized the reaction conditions (Table S1 of the Supporting Information) to a 1:1 ratio of compound **2a** and trimethylamine *N*-oxide (TMAO) with 3 equiv of base in tetrahydrofuran (THF) to achieve diamine **4a** in a 94% yield. With the optimized conditions in hand, we investigated the substrate scope with various imines (Scheme 3).

**Scheme 2:**
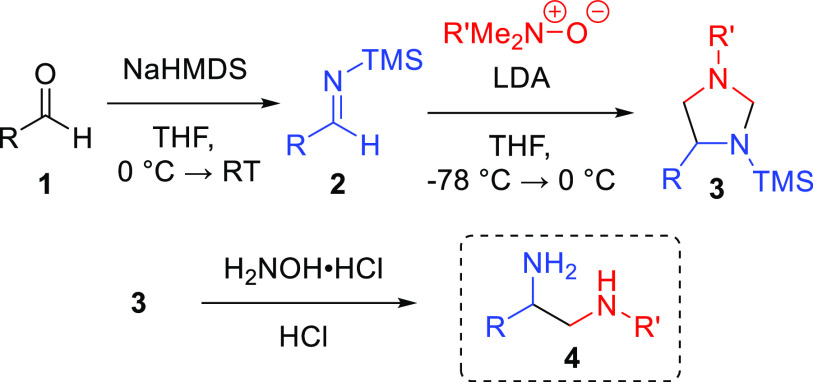
Three-Step One-Pot Synthesis Generating 1,2-Diamines

**Scheme 3:**
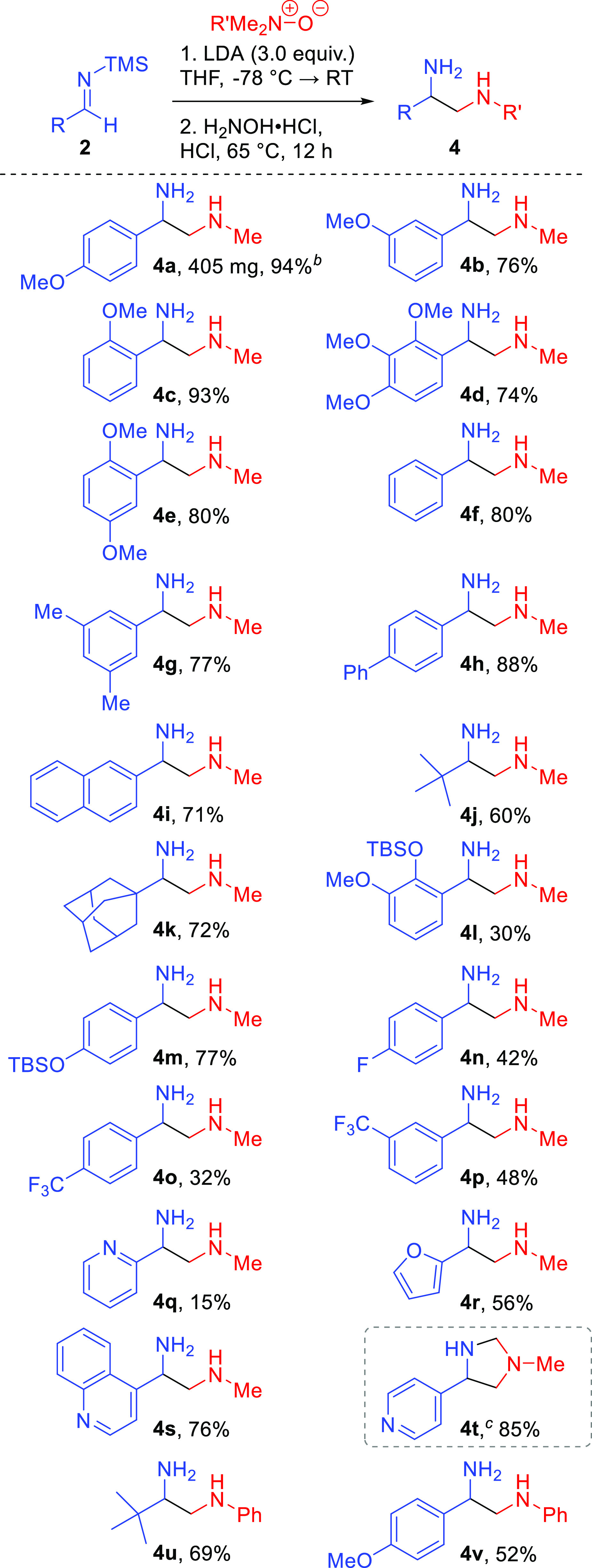
Substrate Scope of Silyl Imines and *N*-Oxides Reactions carried on a 0.4–2.4 mmol scale. Conditions: *N*-oxide (1.0 equiv), LDA (3.0 equiv), THF (0.10 M), silyl imine (1.0 equiv), and from −78 °C to room temperature (RT); hydroxylamine hydrochloride (5.0 equiv), 1.2 M HCl (0.01 M), and 65 °C. The reaction was carried out on a 2.4 mmol scale. Hydroxylamine was not used.

Aromatic silyl imines with electron-donating substituents in the *ortho* and *para* positions benefited the reaction, resulting in excellent yields of 94 and 93% for compounds **4a** and **4c**. The lower yield for compound **4b** is likely due to the low electronic contribution of the methoxy group at the *meta* position. We also observed good yields for compounds **4d** and **4e** of 74 and 80%. Dimethyl-aryl-substituted compound **4g** achieved a 77% yield. Aromatic imines **4f**, **4h**, and **4i** worked well, with yields of 80, 88, and 71% respectively. Gratifyingly, this reaction tolerates alkyl substituents only with non-enolizable protons demonstrated by compounds **4j** and **4k**, with yields of 60 and 72%. Diamine **4l** resulted in a 30% yield, presumably as a result of the steric bulk of the *ortho* OTBS group. This reaction does tolerate silyl-protected alcohols provided that the steric bulk is farther away from the cycloaddition, as demonstrated by compound **4m** giving a 77% yield. This is beneficial because the OTBS can be used as a functional group handle for future chemistry if needed.

Strong electron-withdrawing groups hindered this reaction. Fluoro-substituted compound **4n** resulted in a 42% yield, demonstrating some halide tolerance; however, chloro and bromo were incompatible likely as a result of *ortho*-lithiation or lithium–halide exchange.^19^ Trifluoromethyl diamines **4o** and **4p** also resulted in lower yields of 32 and 48%.

Heteroaryl substituents were also implemented, although pyridyl-substituted compound **4q** was not high yielding (15%). However, furyl-substituted compound **4r** was reasonably tolerated, giving a 56% yield over three steps from 2-furaldehyde. Additionally, quinoline-substituted compound **4s** satisfyingly resulted in a 76% yield. It was found that the imidazolidines for the heterocyclic substituents were fairly stable and did not readily hydrolyze into 1,2-diamines. Pyridyl-substituted compound **4t** was isolated as the imidazolidine in a 85% yield, which shows promising results for future imidazolidine synthesis. Phenyl *N*-oxide was also tolerated (**4u** and **4v**) with alkyl and aryl groups, achieving modest to good yields.

To demonstrate the synthetic utility of these compounds, they were subjected to a variety of different transformations (Scheme 4). Both amines can undergo tosylation, forming compound **5** in a >95% yield. When the reaction is cooled to −78 °C, selective tosylation of the primary amine is observed, forming compound **6** in a 72% yield. Moreover, selective protections of the primary amine are observed with nosyl **7** and pivaloyl **8**, giving 75 and 60% yields, respectively. The synthetic utility of these 1,2-diamines as building blocks for heterocyclics was examined as a result of the presence of the diamine backbone in heterocycles. Diamine **4a** was reacted with triphosgene to afford imidazolidinone **9** in a modest 43% yield. Additionally, compound **4a** and ethyl benzimidate hydrochloride formed 4,5-dihydroimidazole **10** in a 60% yield (Scheme 4). This clearly demonstrates that these 1,2-diamines can be used as synthetic building blocks for either selective functionalization of the amines or the formation of novel heterocycles. This is particularly exciting because structures containing similar cores exhibit bioactivities, such as anti-inflammatory,^20^ antihyperglycemic,^21^ and NF-κB inhibition.^22^

**Scheme 4:**
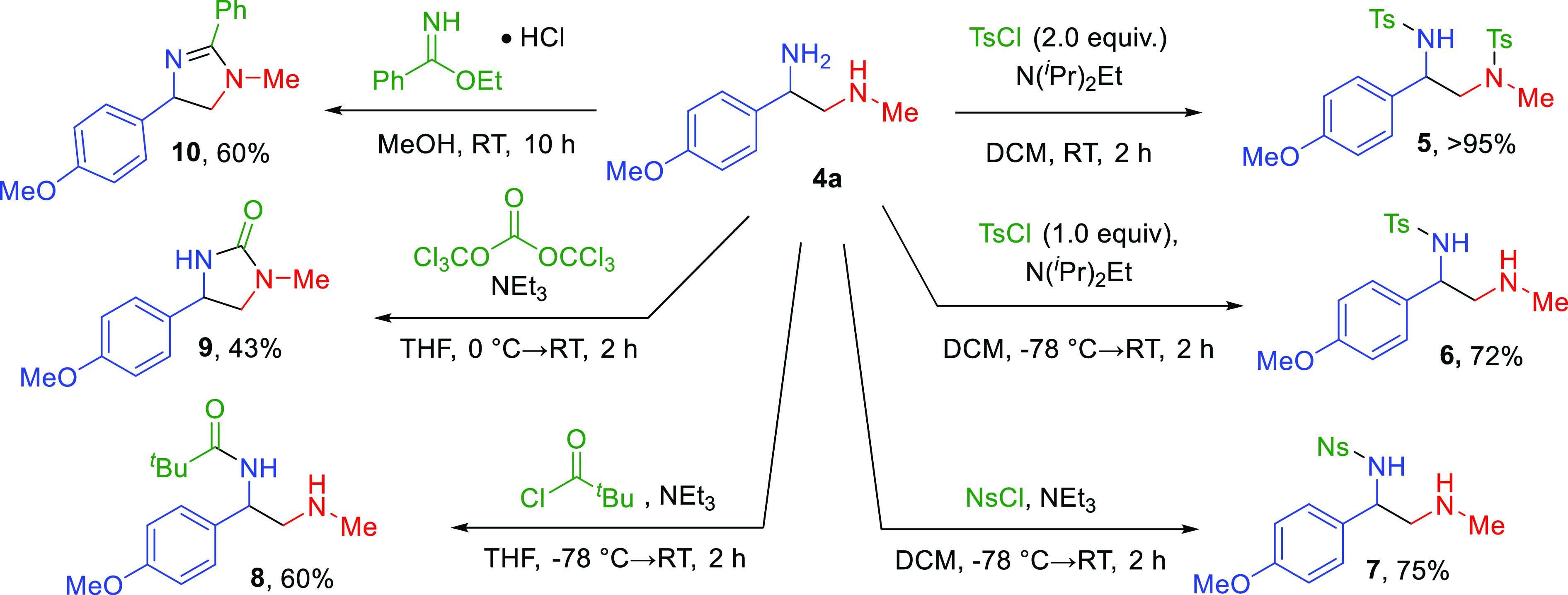
Transformations of 1,2-Diamine **4a**

Computationally, Houk and others have explored similar [3 + 2] cycloaddition reactions, establishing a mechanistic framework for understanding selectivity.^23^ Therefore, using the density functional theory (DFT),^24^ we examined the probable mechanistic pathway that converts trimethylamine *N*-oxide **11** to the requisite 1,3-dipole **14**. Employing the M06-2x functional^25^ with Dunning’s correlation consistent JUL-cc-pVDZ basis set,^26^ all ground and transition structures were calculated (Figure S1 of the Supporting Information). Following our previously published work, two explicit equivalents of LDA/diisopropylamine and THF plus implicit solvation through a polarizable continuum model^27^ were utilized within our method.^16^ These results were consistent with the previously reported mechanism,^16^ where a second deprotonation **12a** is energetically preferred over dissociation of the nitrogen–oxygen bond **12b** (Figure 1). This forms the multi-ion-bridge intermediate **13a**, eliminating the strongly electrophilic iminium intermediate from the mechanism, thus allowing for broader substrate flexibility than previously postulated. Once trimethylamine *N*-oxide has been converted to 1,3-dipole **14**, a [3 + 2] cycloaddition reaction with the silyl-protected imine **2** generates imidazolidine **3**.

**Figure 1:**
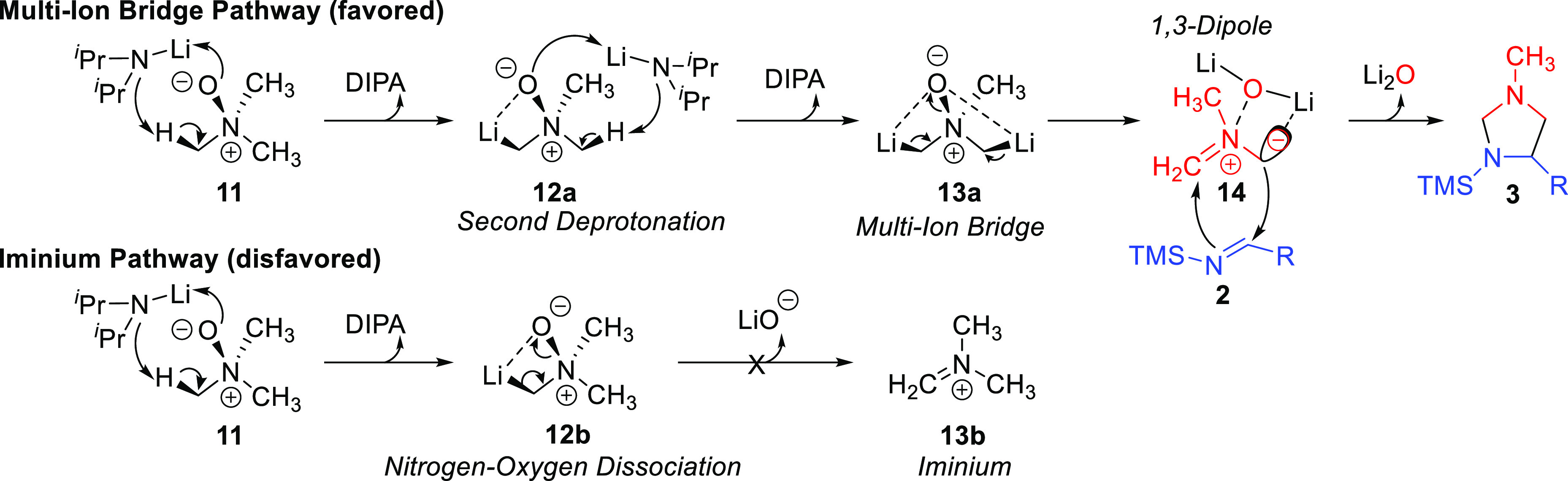
Proposed mechanism for [3 + 2] cycloaddition of TMAO and a silyl imine.

We present here a straightforward, three-step, one-pot synthetic method for producing a wide range of 1,2-diamines in yields up to 94%. The lynchpin [3 + 2] cycloaddition step works best for electron-rich and neutral arenes, with electron-deficient substrates giving lower yields. Moreover, heterocyclics were also well-tolerated, with one resulting in a stable imidazolidine. These products can then undergo further transformations to produce more complex scaffolds via selective functionalization as well as cyclizations. Finally, using DFT modeling, we propose a reasonable mechanism that agrees with our previously reported data. This approach allows for variable substitutions and flexibility that other methods do not offer; therefore, we further expanded the chemical space of these privileged motifs and constructed an array of bioactive targets.

## Data Availability

The data underlying this study are available in the published article and its Supporting Information.

## Supporting Information Available

Experimental protocols, compound characterization, nuclear magnetic resonance (NMR) spectra, and input/output files for computed structures (PDF)
FAIR data, including the primary NMR FID files, for compounds **4a**–**4t** and **5**–**10** (ZIP)
