# Supplementary material for: [3 + 2] Cycloadditions of Tertiary Amine N-Oxides and Silyl Imines as an Innovative Route to 1,2-Diamines
Source: Org Lett. 2023 Jun 15;25(25):4638–43. doi: 10.1021/acs.orglett.3c01396 (PMC10325142; doi:10.1021/acs.orglett.3c01396)
Supplement: Supplementary file 1 — ol3c01396_si_001.zip [file ol3c01396_si_001.zip › 10/10_1HNMR/pdata/1/Exp.html]

Experimentals report

1H NMR (400 MHz, CDCl3) δ 7.74 (d, *J* = 7.4 Hz, 1H), 7.53 (d, *J* = 7.4 Hz, 1H), 7.27 (d, *J* = 8.6 Hz, 1H), 6.82 (d, *J* = 8.6 Hz, 1H), 5.26 (t, *J* = 10.0 Hz, 1H), 4.51 (t, *J* = 11.6 Hz, 1H), 3.69 (d, *J* = 8.2 Hz, 1H).
