# Supplementary material for: [3 + 2] Cycloadditions of Tertiary Amine N-Oxides and Silyl Imines as an Innovative Route to 1,2-Diamines
Source: Org Lett. 2023 Jun 15;25(25):4638–43. doi: 10.1021/acs.orglett.3c01396 (PMC10325142; doi:10.1021/acs.orglett.3c01396)
Supplement: Supplementary file 1 — ol3c01396_si_001.zip [file ol3c01396_si_001.zip › 4b/4b_13CNMR/pdata/1/Exp.html]

Experimentals report

1H NMR (400 MHz, CDCl3) δ 7.26 (m, *J* = 3.9 Hz, 1H), 6.92 (m, *J* = 1.8 Hz, 1H), 6.80 (m, *J* = 2.3 Hz, 1H), 4.04 (q, *J* = 4.4 Hz, 1H), 3.81 (d, *J* = 0.7 Hz, 1H), 2.75 (m, *J* = 3.9 Hz, 1H), 2.44 (d, *J* = 0.9 Hz, 1H).
