# Supplementary figures and images for: [3 + 2] Cycloadditions of Tertiary Amine N-Oxides and Silyl Imines as an Innovative Route to 1,2-Diamines
Source: Org Lett. 2023 Jun 15;25(25):4638–43. doi: 10.1021/acs.orglett.3c01396 (PMC10325142; doi:10.1021/acs.orglett.3c01396)

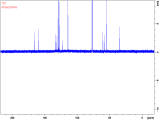

Supplement: Supplementary file 1 — ol3c01396_si_001.zip [file ol3c01396_si_001.zip › 10/10_13CNMR/pdata/1/thumb.png]

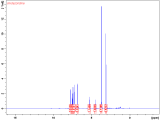

Supplement: Supplementary file 1 — ol3c01396_si_001.zip [file ol3c01396_si_001.zip › 10/10_1HNMR/pdata/1/thumb.png]

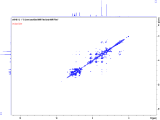

Supplement: Supplementary file 1 — ol3c01396_si_001.zip [file ol3c01396_si_001.zip › 10/10_COSY/pdata/1/thumb.png]

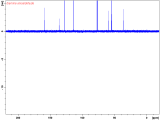

Supplement: Supplementary file 1 — ol3c01396_si_001.zip [file ol3c01396_si_001.zip › 4a/4a_13CNMR/pdata/1/thumb.png]

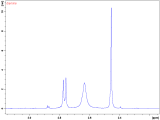

Supplement: Supplementary file 1 — ol3c01396_si_001.zip [file ol3c01396_si_001.zip › 4a/4a_1HNMR/pdata/1/thumb.png]

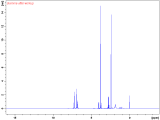

Supplement: Supplementary file 1 — ol3c01396_si_001.zip [file ol3c01396_si_001.zip › 4b/4b_13CNMR/pdata/1/thumb.png]

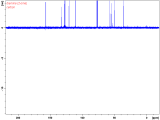

Supplement: Supplementary file 1 — ol3c01396_si_001.zip [file ol3c01396_si_001.zip › 4c/4c_13CNMR/pdata/1/thumb.png]
