# Supplementary material for: [3 + 2] Cycloadditions of Tertiary Amine N-Oxides and Silyl Imines as an Innovative Route to 1,2-Diamines
Source: Org Lett. 2023 Jun 15;25(25):4638–43. doi: 10.1021/acs.orglett.3c01396 (PMC10325142; doi:10.1021/acs.orglett.3c01396)
Supplement: Supplementary file 2 — ol3c01396_si_002.pdf [file ol3c01396_si_002.pdf]

[3+2]-Cycloadditions of Tertiary Amine *N*-oxides and Silyl Imines as an Innovative Route to 1,2-Diamines

Supporting Information

Sarah L. Hejnosz, Danielle R. Beres, Alexander H. Cocolas, Martin J. Neal, Benjamin S. Musiak, Marianne M.B. Hanna, Aaron J. Bloomfield, and Thomas D. Montgomery

Department of Chemistry and Biochemistry, Duquesne University, Pittsburgh PA 15282, United States

Experimental Section General Information……………………………………………………………………………..……..S-2

Optimization of Reaction Conditions………..S-2

General procedures for the preparation of starting materials and products……….………………………………………..S-3

Compound Characterization Section...S-4

^{1}H, ^{13}C, and 2D NMR Spectra……………………………..………………………………………….……………..S-16

Computational Section General Information…………………………………………………………………..……………….S-47

Computational Reaction Coordinate Diagram...S-47

Computational Calculations Outputs…………………………………………………….………………………….………..…S-48

References……………………………………………………………………………..……………………………….S-69

Experimental Section General Information

Nuclear magnetic resonance (NMR) spectra were measured on a Bruker at 400 MHz or 500 MHz. ^{1}H NMR spectra were calibrated from standard TMS (δ 0.0) or solvent resonance ($CDCl_3$: δ 7.27, MeOD: δ 3.31). ^{13}C NMR spectra were calibrated from solvent resonance ($CDCl_3$: δ 77.16, MeOD: δ 49.00). Structural assignments were made with additional information from gCOSY, gHSQC, and gHMBC experiments. High-resolution mass spectrometric analysis (HRMS) was measured on an Agilent Technologies 6530 Accurate-Mass QTOF LC/MS equipped with the Agilent Technologies 1200 series LC system. Infrared (IR) spectral analysis was performed on a Thermoscientific Everest ATR. Reactions monitored by thin layer chromatography (TLC) used TLC Silica gel 60 F_{254} and visualized under a 4-Watt 254/365 nm UV lamp. Flash column chromatography (FCC) (EtOAc/Hex or DCM/MeOH) was performed using a Biotage Isolera One Flash Chromatography instrument with a 25 g Biotage® Sfär Silica D- Duo 60 µm column, and with a 10 g Biotage® Sfär C18 D- Duo 100 Å 30 µm column for reverse phase FCC (0.1% TFA water/MeOH). A heating mantel was used as the heat source for transformations that required heating.

Materials

All materials were used as purchased from MilliporeSigma, Thermo Fisher Scientific, TCI, or Oakwood Chemical, unless otherwise noted. Tetrahydrofuran (THF) was purified by a column of activated alumina and 4 Å molecular sieves via Inert PurSolv Solvent System. Trimethylamine *N*-oxide (TMAO) was stored under rigorous anhydrous conditions with drierite and phosphorus pentoxide. Benzaldehyde was purified by vacuum distillation before use. Solutions of lithium diisopropyl amine (LDA), sodium bis(trimethylsilyl)amide (NaHMDS), and lithium bis(trimethylsilyl)amide were titrated using salicylaldehyde phenylhydrazone before use.[1]

Table S1. Optimization of reaction Conditions[a]

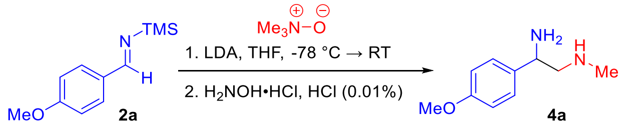

entry	base	solvent	time	yield (%)[b]
1	LDA	THF	3h	70
**2**	**LDA**	**THF**	**2h**	**75**
3	LDA	THF	1h	54
4	LDA (2 equiv.)	THF	2h	50
5	LDA (2.5 equiv)	THF	2h	67
6	LiHMDS	THF	2h	NR
7	NaHMDS	THF	2h	NR
10	LDA	Et_2O	2h	67
11	LDA	TBME	2h	45
12	LDA	THF	2h	90[c] (70)[d]

[a]Reactions were conducted on a 0.4 mmol scale using **2a** (1 equiv.), TMAO (1 equiv.), and LDA (3 equiv.), in 0.1 M THF, followed by hydroxylamine hydrochloride (5 equiv.) and 1.2 M HCl (0.01M). [b]Isolated yields. [c]Reaction was carried out on a 2 mmol scale. [d]Reaction was carried out on a 5 mmol scale.

Our initial conditions involved mixing the 4-methoxyphenyl silyl imine **2a** and TMAO together in a 1:1 ratio with three equivalents of base in THF. Excitingly, following aqueous workup we generated diamine **4a** in good (70%) yield after a three-hour reaction time (Entry 1). Decreasing the reaction time (Entry 2) to two hours gave a mild improvement in yield, (75% yield). Further decreasing the reaction time had a negative impact on the yield, as did using fewer than three equivalents of LDA (Entries 3-5). This agrees with our prior work, where we noted that two equivalents of LDA are required to doubly deprotonate the system, and a third is required to stabilize the transition state by complexing to it.[2]

Neither LiHMDS nor NaHMDS were compatible with the reaction, returning only starting material (Entries 6-7). This was ultimately fortuitous because it meant that excess NaHMDS could be used to form the silyl imine without compromising the later cycloaddition. Finally, neither diethyl ether nor tert-butyl methyl ether offered any advantage over THF (Entry 10-11), again agreeing with prior mechanistic work which indicates that solvent chelation plays a major role.[2-5] We next tested the scalability of this method. Remarkably a 90% yield was achieved when the scale was increased to a 2 mmol scale (Entry 12[c]). When the reaction scale was further increased to a 5 mmol scale the yield was about the same as it was on a 0.4 mmol scale (Entry 12[d]).

General procedure for the preparation of silyl imines (2a-p,u-v)[6, 7]

A solution of *p*-anisaldehyde (0.29 mL, 2.3 mmol, 1.0 equiv.) in dry THF (2.3 mL, 1.0 M) maintained under a positive pressure of nitrogen was stirred and cooled to 0 °C. A solution of 0.79 M NaHMDS (2.9 mL, 2.3 mmol, 1.0 equiv.) was added dropwise via syringe and the reaction mixture was allowed to warm up to room temperature over the course of two hours. ^{1}H NMR confirmed the formation of the silyl imine with the disappearance of the aldehyde proton around 10 ppm and the appearance of the imine proton at around 9 ppm.

General procedure for the preparation of silyl imines (2q-t)

A solution of aldehyde (1.2 mmol, 1.0 equiv.) in dry THF (1.2 mL, 1.0 M) maintained under a positive pressure of nitrogen was stirred and cooled to -78 °C in a dry ice/acetone bath. A solution of 1.0 M LiHMDS (1.2 mL, 1.2 mmol, 1.0 equiv.) was added dropwise via syringe and the reaction mixture. After stirring for 30 min at -78 °C, the solution was allowed to warm up to room temperature and stir for an additional 30 min. ^{1}H NMR confirmed the formation of the silyl imine with the disappearance of the aldehyde proton around 10 ppm and the appearance of the imine proton at around 9 ppm.

General procedure for the preparation of imidazolidines (3)

A solution of trimethylamine N-oxide (182 mg, 2.4 mmol, 1.0 equiv.) in dry THF (7.0 mL, 0.10 M) maintained under a positive pressure of nitrogen was stirred and cooled to -78°C. A solution of 1.7 M LDA (4.0 mL, 6.9 mmol, 3.0 equiv.) was added dropwise via syringe, followed by the slow addition of the crude 0.46 M silyl imine (4.0 mL, 2.3 mmol, 1.0 equiv.). LDA was titrated before use using salicylaldehyde phenylhydrazone.[1] The reaction was removed from the cold bath and allowed to stir and warm to room temperature for 2 h. The formation of the imidazolidine was confirmed by ^{1}H NMR and was taken onto the next step directly without further purification.

General procedure for the preparation of 1,2 diamines (4a-s,u-v)

Under ambient conditions, hydroxylamine hydrochloride (850 mg, 12 mmol, 5.2 equiv.) was added to the crude reaction mixture of the imidazolidine along with 1.2 M HCl (0.20 mL, 0.01 M) The reaction vessel was equipped with a reflux condenser and the mixture was heated to 65 °C using a heating mantle for 12 h. The reaction was cooled to RT, acidified using 1.0 M HCl and washed with DCM (20 mL x 3), hexanes (x 2), and EtOAc (x 3). The aqueous layer was basified with 15% NaOH and the product extracted with DCM (20 mL x 3). The organic layers were combined, dried over $MgSO_4$, and concentrated under reduced pressure to afford pure 1,2 diamines unless otherwise noted. Product was isolated as a colorless oil. (94% yield, 405 mg, 2.2 mmol).

General procedure for the preparation of imidazolidine (4t)

Volatiles were removed from the crude reaction mixture of the imidazolidine under reduced pressure. The residue was dissolved in THF (10 mL, 0.10 M)) and 1.0 M HCl (5 mL, 0.20 M) The reaction vessel was equipped with a reflux condenser and the mixture was heated to 65 °C for 12 h. The reaction was cooled to RT, transferred to a separatory funnel, and washed with DCM (20 mL x 3), hexanes (x 2), and EtOAc (x 3). The aqueous layer was basified with 15% NaOH and the product extracted with DCM (20 mL x 3). The organic layers were combined, dried over $MgSO_4$, and concentrated under reduced pressure to afford pure 1,2 diamines unless otherwise noted. Product was isolated as an orange oil. (85% yield, 69 mg, 0.43 mmol).

1-(4-methoxyphenyl)-*N*^{2}-methylethane-1,2-diamine (4a)

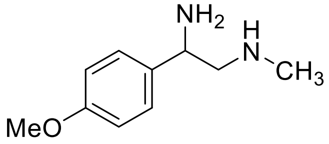

Silyl imine prepared according to the general procedure using *p*-anisaldehyde (0.29 mL, 2.3 mmol, 1.0 equiv.), 0.79 M NaHMDS (2.9 mL, 2.3 mmol, 1.0 equiv.), and THF (2.3 mL, 1.0 M). Silyl imine solution was used without further purification.

Diamine was prepared according to the general procedure using TMAO (182 mg, 2.4 mmol, 1.0 equiv.), 1.7 M LDA (4.0 mL, 6.9 mmol, 3.0 equiv.), crude 0.46 M silyl imine (4.0 mL, 2.3 mmol, 1.0 equiv.), THF (7.0 mL, 0.10 M), hydroxylamine hydrochloride (850 mg, 12 mmol, 5.2 equiv.) and 1.2 M HCl (0.20 mL, 0.01 M). Following aqueous work-up, no further purification was necessary. Product was isolated as a colorless oil. (94% yield, 405 mg, 2.2 mmol)

^{1}H NMR (400 MHz, $CDCl_3$) δ 7.26 (d, *J* = 8.7 Hz, 2H), 6.87 (d, *J* = 8.7 Hz, 2H), 4.03 (t, *J* = 6.7 Hz, 1H), 3.79 (s, 3H), 2.76 – 2.71 (m, 2H), 2.44 (s, 3H), 2.14 (br. s, 3H).

^{13}C NMR (101 MHz, $CDCl_3$) δ 158.8, 136.1, 127.5, 114.0, 59.4, 55.3, 54.3, 36.0.

IR: 2914, 2778, 1739, 1653, 1618, 1604, 1577, 1512, 1466, 1446, 1439, 1424, 1402, 1356, 1308, 1278, 1261, 1224, 1188, 1146, 1108, 1088, 1032, 1001, 970, 954, 940, 909, 883, 825, 808, 733, 715, 645, 569, 540, 507 cm^{-1}

HRMS (ESI) m/z: $[M+H]^+$ Calc'd for $C_{10}H_{17}N_2O$ 181.1336; Found 181.1340.

1-(3-methoxyphenyl)-*N*^{2}-methylethane-1,2-diamine (4b)

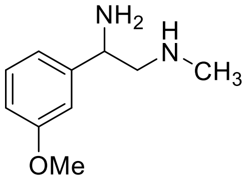

Silyl imine prepared according to the general procedure using *m*-anisaldehyde (0.29 mL, 2.4 mmol, 1.0 equiv.), 1.9 M NaHMDS (1.3 mL, 2.4 mmol, 1.0 equiv.), and THF (2.4 mL, 1.0 M). Silyl imine solution was used without further purification.

Diamine was prepared according to the general procedure using TMAO (180 mg, 2.4 mmol, 1.0 equiv.), 1.6 M LDA (4.5 mL, 7.2 mmol, 3.0 equiv.), crude 0.68 M silyl imine solution (3.5 mL, 2.4 mmol, 1.0 equiv.), THF (12 mL, 0.10 M), hydroxylamine hydrochloride (833 mg, 12 mmol, 5.0 equiv.) and 1.2 M HCl (0.2 mL, 0.01 M). Following aqueous work-up, no further purification was necessary. Product was isolated as a yellow oil. (76% yield, 328 mg, 1.8 mmol)

^{1}H NMR (400 MHz, $CDCl_3$) δ 7.26 (t, *J* = 7.7 Hz, 1H), 6.93 (s, 1H), 6.91 (d, *J* = 8.4 Hz, 1H), 6.80 (dd, *J* = 8.0, 2.6 Hz, 1H), 4.04 (dd, *J* = 7.8, 5.3 Hz, 1H), 3.81 (s, 3H), 2.81 – 2.70 (m, 2H), 2.44 (s, 3H), 1.88 (br. s, 3H).

^{13}C NMR (101 MHz, $CDCl_3$) δ 160.0, 146.3, 129.7, 118.8, 112.7, 112.1, 59.7, 55.4, 55.3, 36.8.

IR: 3290, 2937, 2836, 2793, 1668, 1599, 1584, 1486, 1464, 1453, 1435, 1377, 1347, 1317, 1254, 1152, 1112, 1078, 1040, 995, 855, 780, 740, 699, 629, 579, 564, 541 cm^{-1}

HRMS (ESI) m/z (z=2): [M+H+Na]+2 Calc'd for $C_{10}H_{17}N_2ONa$ 102.0614; Found 102.0615.

1-(2-methoxyphenyl)-*N*^{2}-methylethane-1,2-diamine (4c)

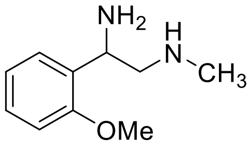

Silyl imine prepared according to the general procedure using *o*-anisaldehyde (328 mg, 2.4 mmol, 1.0 equiv.), 1.9 M NaHMDS (1.3 mL, 2.4 mmol, 1.0 equiv.), and THF (2.4 mL, 1.0 M). Silyl imine solution was used without further purification.

Diamine was prepared according to the general procedure using TMAO (193 g, 2.4 mmol, 1.0 equiv.), 1.9 M LDA (3.7 mL, 7.2 mmol, 3.0 equiv.), crude 0.60 M silyl imine (4.0 mL, 2.4 mmol, 1.0 equiv.), THF (12 mL, 0.10 M), hydroxylamine hydrochloride (834 g, 12 mmol, 5.0 equiv.) and 1.2 M HCl (0.20 mL, 0.01 M). Following aqueous work-up, no further purification was necessary. Product was isolated as a yellow oil. (93% yield, 396 mg, 2.2 mmol)

^{1}H NMR (400 MHz, $CDCl_3$) δ 7.31 (dd, *J* = 7.5, 1.7 Hz, 1H), 7.26 – 7.22 (m, 1H), 6.96 (td, *J* = 7.6, 6.3 Hz, 1H), 6.88 (dd, *J* = 8.2, 1.1 Hz, 1H), 4.34 (dd, *J* = 8.4, 4.8 Hz, 1H), 3.84 (s, 3H), 2.88 – 2.77 (m, 2H), 2.48 (s, 3H), 2.40 (br. s, 3H)

^{13}C NMR (101 MHz, $CDCl_3$) δ 157.0, 132.3, 128.2, 127.1, 120.8, 110.6, 57.4, 55.3, 50.2, 36.0.

IR: 3285, 2937, 2837, 2793, 1905, 1600, 1586, 1490, 1462, 1438, 1374, 1289, 1237, 1179, 1161, 1105, 1050, 1025, 932, 878, 810, 752, 703, 666, 617, 602, 579, 557, 542 cm^{-1}

HRMS (ESI) m/z: $[M+H_3O]^+$ Calc'd $C_{10}H_{19}N_2O_2$ 199.1441; Found 199.1437.

N^1-methyl-2-(2,3,4-trimethoxyphenyl)ethane-1,2-diamine (4d)

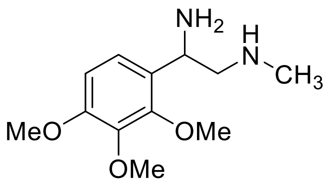

Silyl imine prepared according to the general procedure using 2,3,4-trimethoxybenzaldehyde (237 mg, 1.2 mmol, 1.0 equiv.), 1.9 M NaHMDS (0.63 mL, 1.2 mmol, 1.0 equiv.), and THF (1.2 mL, 1.0 M). Silyl imine solution was used without further purification.

Diamine was prepared according to the general procedure using TMAO (107 mg, 1.4 mmol, 1.4 equiv.), 1.8 M LDA (1.6 mL, 3.0 mmol, 3.0 equiv.), crude 0.67 M silyl imine (1.5 mL, 1.0 mmol, 1.0 equiv.), THF (10 mL, 0.10 M), hydroxylamine hydrochloride (354 g, 5.0 mmol, 5.0 equiv.) and 1.2 M HCl (0.10 mL, 0.01 M). Following aqueous work-up, no purification was necessary. Product was isolated as a yellow oil. (74% yield, 178mg, 0.74 mmol)

^1H NMR (400 MHz, $CDCl_3$) δ 7.02 (d, J = 8.7 Hz, 1H), 6.66 (d, J = 8.6 Hz, 1H), 4.28 (dd, J = 8.3, 4.9 Hz, 1H), 3.92 (s, 3H), 3.86 (s, 3H), 3.85 (s, 3H), 2.83 – 2.72 (m, 2H), 2.48 (s, 3H), 2.35 (br. s, 3H)

^{13}C NMR (126 MHz, $CDCl_3$) δ 153.0, 151.6, 142.2, 130.0, 121.3, 107.4, 61.3, 60.8, 58.4, 56.1, 49.7, 36.0.

IR: 3263, 2940, 2837, 1599, 1494, 1463, 1435, 1417, 1390, 1282, 1230, 1201, 1091, 1011, 964, 904, 853, 797, 728, 688, 666, 645, 602, 582, 574, 559, 539 cm^{-1}

HRMS (ESI) m/z: $[M+Na]^+$ Calc'd for $C_{12}H_{20}N_2O_3Na$ 263.1366; Found 263.1359.

1-(2,5-dimethoxyphenyl)-N^2-methylethane-1,2-diamine (4e)

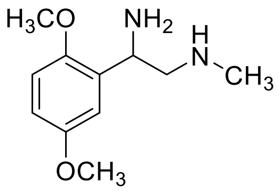

Silyl imine prepared according to the general procedure using 2,5-dimethoxybenzaldehyde (185 mg, 1.1 mmol, 1.0 equiv.), 1.2 M NaHMDS (1.0 mL, 1.2 mmol, 1.1 equiv.), and THF (1.1 mL, 1.0 M). Silyl imine solution was used without further purification.

Diamine was prepared according to the general procedure using TMAO (78 mg, 1.0 mmol, 1.0 equiv.), 2.7 M LDA (1.1 mL, 3.0 mmol, 3.0 equiv.), crude 0.5 M silyl imine (2 mL, 1.0 mmol, 1.0 equiv.), THF (7.0 mL, 0.10 M), hydroxylamine hydrochloride (416 mg, 6.0 mmol, 6.0 equiv.) and 1.2 M HCl Following aqueous work-up, no further purification was necessary. Product was isolated as a yellow oil. (80% yield, 168 mg, 0.80 mmol)

^1H NMR (400 MHz, $CDCl_3$) δ 6.93 (d, J = 3.1 Hz, 1H), 6.79 (d, J = 8.6 Hz, 1H), 6.73 (d, J = 8.7 Hz, 1H), 4.31 (t, 1H), 3.78 (s, 3H), 3.76 (s, 3H), 2.84 – 2.79 (m, 1H), 2.72 (dd, J = 11.6, 8.3 Hz, 1H), 2.45 (s, 3H), 2.03 (br. s, 3H).

^{13}C NMR (101 MHz, $CDCl_3$) δ 153.7, 151.1, 133.5, 113.4, 112.1, 111.5, 57.4, 55.8, 55.8, 50.2, 36.0.

IR: 2937, 2834, 2792, 1589, 1493, 1464, 1428, 1369, 1275, 1214, 1178, 1155, 1104, 1044, 1024, 871, 801, 732, 713, 603 cm^{-1}

HRMS (ESI) m/z: $[M+H_3O]^+$ Calc'd for $C_{11}H_{21}N_2O_3$ 229.1547; Found 229.1567.

N^1-methyl-2-phenylethane-1,2-diamine (4f)

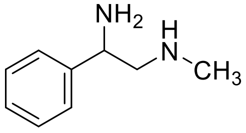

Silyl imine prepared according to the general procedure using benzaldehyde (0.10 mL, 1.0 mmol, 1.0 equiv.), 1.1 M NaHMDS (0.9 mL, 1.0 mmol, 1.0 equiv.), and THF (1.0 mL, 1.0 M). Silyl imine solution was used without further purification.

Diamine was prepared according to the general procedure using TMAO (34 mg, 0.40 mmol, 1.0 equiv.), 2.9 M LDA (0.41 mL, 1.2 mmol, 3.0 equiv.), crude 0.53 M silyl imine (0.80 mL, 0.40 mmol, 1.0 equiv.), THF (3.2 mL, 0.10 M), hydroxylamine hydrochloride (192 g, 2.0 mmol, 5.0 equiv.) and 1.2 M HCl (0.4 mL, 0.01 M). Following aqueous work-up, no further purification was necessary. Product was isolated as a yellow oil. (80% yield, 48 mg, 0.32 mmol). The spectrum is in agreement with previously reported.[8]

^{1}H NMR (400 MHz, $CDCl_3$) δ 7.33 – 7.30 (m, 4H), 7.28 – 7.22 (m, 1H), 4.06 – 4.02 (dd, J = 7.8, 5.4 Hz, 1H), 2.78 – 2.69 (m, 2H) 2.42 (s, 3H).

1-(3,5-dimethylphenyl)-*N*^{2}-methylethane-1,2-diamine (4g)

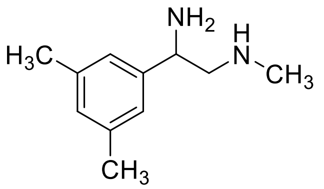

Silyl imine prepared according to the general procedure using 3,5-dimethylbenzaldehyde (0.16 mL, 1.2 mmol, 1.0 equiv.), 1.2 M NaHMDS (1.0 mL, 1.2 mmol, 1.0 equiv.), and THF (1.0 mL, 1.0 M). Silyl imine solution was used without further purification.

Diamine was prepared according to the general procedure using TMAO (80 mg, 1.0 mmol, 1.0 equiv.), 1.5 M LDA (2.0 mL, 3.0 mmol, 3.0 equiv.), crude 0.55 M silyl imine solution (1.8 mL, 1.0 mmol, 1.0 equiv.), THF (8.0 mL, 0.1 M), hydroxylamine hydrochloride (351 g, 5.0 mmol, 5.0 equiv.) and 1.2 M HCl (0.10 mL, 0.01 M). Following aqueous work-up, no further purification was necessary. Product was isolated as a yellow oil. (77% yield, 132 mg, 0.77 mmol)

^{1}H NMR (400 MHz, $CDCl_3$) δ 6.95 (s, 2H), 6.89 (s, 1H), 3.98 (t, 1H), 2.78 – 2.68 (m, 2H), 2.43 (s, 3H), 2.31 (s, 6H), 2.06 (br. s, 3H)

^{13}C NMR (101 MHz, $CDCl_3$) δ 144.5, 138.0, 128.8, 124.2, 59.8, 55.2, 36.3, 21.3.

IR: 3002, 2917, 2887, 2793, 2135, 1639, 1608, 1566, 1449, 1418, 1379, 1298, 1275, 1232, 1167, 1112, 1080, 1040, 975, 927, 909, 845, 806, 730, 705, 697, 634, 620 cm^{-1}

HRMS (ESI) m/z: $[M+CH_3OH_2]^+$ Calc'd for $C_{12}H_{23}N_2O$ 211.1805; Found 211.1819.

1-([1,1'-biphenyl]-4-yl)-*N*^{2}-methylethane-1,2-diamine (4h)

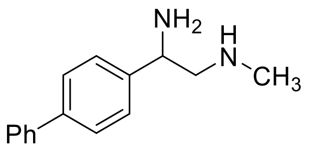

Silyl imine prepared according to the general procedure using [1,1'-biphenyl]-4-carbaldehyde (222 mg, 1.2 mmol, 1.0 equiv.), 1.9 M NaHMDS (0.63 mL, 1.2 mmol, 1.0 equiv.), and THF (1.2 mL, 1.0 M). Silyl imine solution was used without further purification.

Diamine was prepared according to the general procedure using TMAO (93 mg, 1.2 mmol, 1.0 equiv.), 0.86 M LDA (1.4 mL, 1.2 mmol, 3.0 equiv.), crude 0.68 M silyl imine solution (1.8 mL, 1.2 mmol, 1.0 equiv.), THF (7.0 mL, 0.10 M), hydroxylamine hydrochloride (418 mg, 6.0 mmol, 5.0 equiv.) and 1.2 M HCl (0.12 mL, 0.01M). Following aqueous work-up, no further purification was necessary. Product was isolated as a yellow oil. (88% yield, 233 mg, 1.0 mmol)

^{1}H NMR (400 MHz, $CDCl_3$) δ 7.58 – 7.54 (m, 4H), 7.43 – 7.38 (m, 4H), 7.31 (tt, J = 7.4, 1.3 Hz, 1H) 4.08 (dd, J = 7.9, 5.3 Hz, 1H), 2.82 – 2.71 (m, 2H), 2.44 (s, 3H), 1.86 (br. s, 3H)

^{13}C NMR (101 MHz, $CDCl_3$) δ 143.7, 140.8, 140.0, 128.7, 127.2, 127.2, 127.0, 126.9, 59.9, 55.0, 36.4.

IR: 3346, 3057, 3027, 2947, 2837, 2778, 1674, 1599, 1487, 1454, 1428, 1151, 1007, 839, 764 cm^{-1}

HRMS (ESI) m/z: $[M+H]^+$ Calc'd for $C_{15}H_{19}N_2$ 227.1543; Found 227.1548.

N^1-methyl-2-(naphthalen-2-yl)ethane-1,2-diamine (4i)

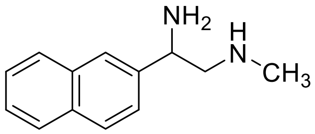

Silyl imine prepared according to the general procedure using 2-naphthaldehyde (391 mg, 2.5 mmol, 1.0 equiv.), 1.8 M NaHMDS (1.4 mL, 2.5 mmol, 1.0 equiv.), and THF (2.5 mL, 1.0 M). Silyl imine solution was used without further purification.

Diamine was prepared according to the general procedure using TMAO (184 mg, 2.4 mmol, 1.0 equiv.), 1.4 M LDA (5.2 mL, 7.2 mmol, 3.0 equiv.), crude 0.64 M silyl imine (3.8 mL, 2.4 mmol, 1.0 equiv.), THF (16 mL, 0.10 M), hydroxylamine hydrochloride (835 mg, 12 mmol, 5.0 equiv.) and 1.2 M HCl (0.24 mL, 0.01 M). Following aqueous work-up, no further purification was necessary. Product was isolated as a yellow oil, (71% yield, 341 mg, 1.8 mmol)

^{1}H NMR (400 MHz, $CDCl_3$) δ 7.85 – 7.81 (m, 4H), 7.50 – 7.46 (m, 3H), 4.25 (dd, *J* = 7.8, 5.3 Hz, 1H), 2.91 – 2.80 (m, 2H), 2.47 (s, 3H), 2.14 (br. s, 3H).

^{13}C NMR (101 MHz, $CDCl_3$) δ 142.1, 133.5, 133.0, 128.4, 127.9, 127.8, 126.3, 125.8, 125.1, 124.9, 59.8, 55.5, 36.5.

IR: 3275, 3053, 2931, 2845, 2790, 1633, 1601, 1507, 1447, 1365, 1270, 1125, 1018, 892, 856, 817, 744, 659, 540 cm^{-1}

HRMS (ESI) m/z: $[M+CH_3OH_2]^+$ Calc'd for $C_{14}H_{21}N_2O$ 233.1648; Found 233.1636.

N^1,3,3-trimethylbutane-1,2-diamine (4j)

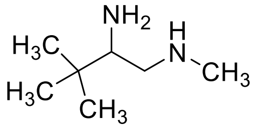

Silyl imine prepared according to the general procedure using pivaldehyde (0.13 mL, 1.2 mmol, 1.0 equiv.), 1.2 M NaHMDS (1.0 mL, 1.2 mmol, 1.0 equiv.), and THF (1.2 mL, 1.0 M). Silyl imine solution was used without further purification.

Diamine was prepared according to the general procedure using TMAO (76 mg, 1.0 mmol, 1.0 equiv.), 2.7 M LDA (1.1 mL, 3.0 mmol, 3.0 equiv.), crude 0.55 M silyl imine (2.0 mL, 1.0 mmol, 1.0 equiv.), THF (8.0 mL, 0.10 M), hydroxylamine hydrochloride (368 mg, 5.0 mmol, 5.0 equiv.) and 1.2 M HCl (0.10 mL, 0.01 M). Following aqueous work-up, product was purified by acidifying with 10% HCl until precipitate formed, removing solvent, and washing with cold DCM. Isolated as an off-white solid. (60% yield, 78 mg, 0.60 mmol)

^{1}H NMR (400 MHz, MeOD) δ 3.48 (dd, *J* = 35.6, 11.7 Hz, 2H), 3.25 (dd, *J* = 14.3, 9.0 Hz, 1H), 2.79 (br. s, 3H), 1.07 (s, 9H).

^{13}C NMR (101 MHz, MeOD) δ 59.3, 50.2, 34.7, 34.4, 26.4.

IR: 2965, 2692, 2384, 2131, 1591, 1519, 1474, 1408, 1380, 1350, 1125, 1037, 1012, 932, 810, 750, 553, 511, 460, 423 cm^{-1}

HRMS (ESI) m/z: $[M+H]^+$ Calc'd for $C_7H_{19}N_2$ 131.1543; Found 131.1553.

1-((3*r*,5*r*,7*r*)-adamantan-1-yl)-N^2-methylethane-1,2-diamine (4k)

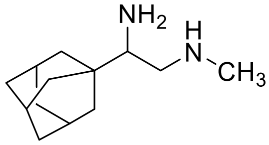

Silyl imine prepared according to the general procedure using adamantane-1-carbaldehyde (80 mg, 0.50 mmol, 1.0 equiv.), 1.0 M NaHMDS (0.50 mL, 0.50 mmol, 1.0 equiv.), and THF (0.50 mL, 1.0 M). Silyl imine solution was used without further purification.

Diamine was prepared according to the general procedure using TMAO (37.7 mg, 0.50 mmol, 1.3 equiv.), 1.5 M LDA (0.80 mL, 1.2 mmol, 3.0 equiv.), crude 0.50 M silyl imine (0.80 mL, 0.40 mmol, 1.0 equiv.), THF (4.0 mL, 0.10 M), hydroxylamine hydrochloride (150 mg, 2.2 mmol, 5.5 equiv.) and 1.2 M HCl (0.04 mL, 0.01M). Following aqueous work-up, no further purification was necessary. Product was isolated as a white powder. (72% yield, 60 mg, 0.28 mmol).

^{1}H NMR (400 MHz, CDCl3) δ 2.75 (dd, J = 10.9, 2.1 Hz, 1H), 2.43 (s, 3H), 2.34 – 2.24 (m, 2H), 1.96 (br. s, 3H), 1.72 – 1.58 (m, 8H), 1.52 (s, 7H).

^{13}C NMR (101 MHz, CDCl3) δ 60.0, 52.6, 38.6, 37.3, 36.4, 35.5, 28.5.

IR: 3366, 3314, 2900, 2846, 2679, 2464, 2190, 2063, 1591, 1449, 1363, 1344, 1316, 1299, 1250, 1184, 1139, 1123, 1099, 1037, 983, 937, 921, 894, 811, 771, 712, 632, 576, 563, 541 cm^{-1}

HRMS (ESI) m/z: $[M+Na]^+$ Calc'd for $C_{13}H_{24}N_2Na$ 231.1832; Found 231.1840.

2-((tert-butyldimethylsilyl)oxy)-3-methoxybenzaldehyde (SI-1)

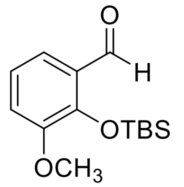

Prepared according to literature procedure.[9] An oven dried round bottom flask under positive nitrogen pressure was charged with *o*-vanillin (1.523 g, 10 mmol, 1.0 equiv.) and it was dissolved in DCM (50 mL). 4-Dimethylaminopyridine (65 mg, 0.53 mmol, 0.053 equiv), and triethylamine (2.1 mL, 15 mmol, 1.5 equiv.) were added to the reaction and the mixture was cooled to 0°C. *tert*-Butyldimethylsilyl chloride (2.3601 g, 15 mmol, 1.5 equiv.) was added portionwaise and the reaction was allowed to warm up to RT. The reaction was monitored by TLC (1:1 Hex/EtOAc). Once complete the reactions mixture was poured into cold brine, extracted with DCM (x 3), dried over $MgSO_4$, and concentrated *in vacuo*. The residue was purified using FCC to afford a clear oil. (98% yield, 2.610 g, 9.8 mmol). R_f 0.95. The ^{1}H NMR is in agreement with that previously reported.[10]

^{1}H NMR (400 MHz, $CDCl_3$) δ 10.53 (s, 1H), 7.40 (dd, *J* = 7.9, 1.6 Hz, 1H), 7.17 – 6.83 (m, 2H), 3.85 (s, 3H), 1.01 (s, 9H), 0.23 (s, 6H).

1-(2-((tert-butyldimethylsilyl)oxy)-3-methoxyphenyl)-*N*^{2}-methylethane-1,2-diamine (4l)

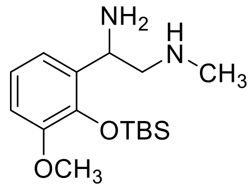

Silyl imine prepared according to the general procedure using 2-((tert-butyldimethylsilyl)oxy)-3-methoxybenzaldehyde (319 mg, 1.2 mmol, 1.0 equiv.), 1.0 M NaHMDS (1.2 mL, 1.2 mmol, 1.0 equiv.), and THF (1.2 mL, 1.0 M). Silyl imine solution was used without further purification.

Diamine was prepared according to the general procedure using TMAO (77 mg, 1.0 mmol, 1.0 equiv.), 1.5 M LDA (2.0 mL, 3.0 mmol, 3.0 equiv.), crude 0.50 M silyl imine (1.0 mmol, 1.0 equiv.), THF (8.0 mL, 0.10 M), hydroxylamine hydrochloride (349 mg, 5.0 mmol, 5.0 equiv.) and 1.2 M HCl (0.10 mL, 0.01 M). The standard aqueous work-up was followed with careful pH control. The solution was acidified to a pH and 4 and then basified to a pH of 10. No further purification was necessary. Product was isolated as a brown oil. (30% yield, 94 mg, 0.30 mmol)

^{1}H NMR (400 MHz, $CDCl_3$) δ 6.96 (dd, *J* = 7.8, 1.7 Hz, 1H), 6.91 (t, *J* = 7.8 Hz, 1H), 6.75 (dd, *J* = 7.9, 1.8 Hz, 1H), 4.51 (dd, *J* = 8.3, 4.4 Hz, 1H), 3.78 (s, 3H), 2.83 – 2.68 (m, 2H), 2.44 (s, 3H), 1.00 (s, 9H), 0.22 (s, 3H), 0.19 (s, 3H).

^{13}C NMR (101 MHz, $CDCl_3$) δ 149.7, 142.1, 135.8, 121.1, 118.1, 109.9, 57.9, 54.8, 48.2, 36.5, 26.2, 19.1, -3.5, -3.7.

IR: 2952, 2929, 2896, 2855, 2800, 1584, 1472, 1463, 1441, 1387, 1361, 1251, 1226, 1183, 1062, 1007, 907, 832, 810, 778, 733, 664, 610, 585, 574, 562, 551, 541, 531, 523 cm^{-1}

HRMS (ESI) m/z (z=2): $[M+2H]^{+2}$ Calc'd for $C_{16}H_{32}N_2O_2Si$ 156.1111; Found 156.1103.

4-((tert-butyldimethylsilyl)oxy)benzaldehyde (SI-2)

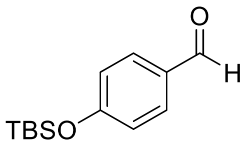

Prepared according to literature procedure.[9] 4-Hydroxybenzaldehye (610 mg, 5.0 mmol, 1.0 equiv.), DCM (30 mL), 4-dimethylaminopyridine (40 mg, 0.33 mmol, 0.066 equiv.), triethylamine (1.1 mL, 7.5 mmol, 1.5 equiv.) and *tert*-Butyldimethylsilyl chloride (1.090 g, 7.5 mmol, 1.5 equiv.) were used. The residue was purified by FCC, TLC conditions 1:1 Hex/EtOAc, R_f =0.95. (83% yield, 982 mg, 4.2 mmol). The ^{1}H NMR is in agreement with that previously reported.[11]

^{1}H NMR (400 MHz, $CDCl_3$) δ 9.90 (s, 1H), 7.80 (d, *J* = 8.7 Hz, 2H), 6.95 (d, *J* = 8. Hz, 2H), 1.00 (s, 9H), 0.26 (s, 6H).

1-(4-((*tert*-butyldimethylsilyl)oxy)phenyl)-N^2-methylethane-1,2-diamine (4m)

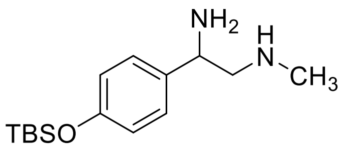

Silyl imine prepared according to the general procedure using 4-((tert-butyldimethylsilyl)oxy)benzaldehyde (595 mg, 2.4 mmol, 1.0 equiv.), 1.9 M NaHMDS (1.3 mL, 2.5 mmol, 1.0 equiv.), and THF (2.4 mL, 1M). Silyl imine solution was used without further purification.

Diamine was prepared according to the general procedure using TMAO (180 mg, 2.4 mmol, 1.0 equiv.), 1.6 M LDA (4.5 mL, 7.2 mmol, 3.0 equiv.), crude 0.66 M silyl imine (3.7 mL, 2.4 mmol, 1.0 equiv.), THF (16 mL, 0.10 M), hydroxylamine hydrochloride (840 mg, 12 mmol, 5.0 equiv.) and 1.2 M HCl (0.24 mL, 0.01 M). The standard aqueous work-up was followed with careful pH control. The solution was acidified to a pH and 4 and then basified to a pH of 10. No further purification was necessary. Product was isolated as a yellow oil. (77% yield, 518 mg, 1.8 mmol)

^{1}H NMR (400 MHz, $CDCl_3$) δ 7.16 (d, *J* = 8.5 Hz, 2H), 6.76 (d, *J* = 8.5 Hz, 2H), 4.02 (d, *J* = 7.6 Hz, 1H), 3.32 (br. s, 3H), 2.74 (dd, *J* = 6.8, 2.7 Hz, 2H), 2.41 (s, 3H), 0.94 (s, 9H), 0.15 (s, 6H).

^{13}C NMR (126 MHz, $CDCl_3$) δ 161.9, 134.8, 128.4, 127.5, 123.7, 120.3, 115.0, 36.0, 27.2, 25.8, 17.8, -4.5.

IR: 2951 2927 2882 2853 1593 1510 1470 1409 1361 1252 1238 1140 1072 1007 908 872 834 822 808 768 730 691 665 600 577 cm^{-1}

HRMS (ESI) m/z (z=2): $[M+2H]^{+2}$ Calc'd for $C_{15}H_{30}N_2OSi$ 141.1058; Found 141.1057.

1-(4-fluorophenyl)-N^2-methylethane-1,2-diamine (4n)

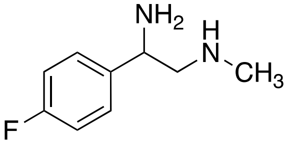

Silyl imine prepared according to the general procedure using 4-fluorobenzaldehyde (0.05 mL, 0.50 mmol, 1.0 equiv.), 0.86 M NaHMDS (0.58 mL, 0.50 mmol, 1.0 equiv.), and THF (0.50 mL, 1.0 M). Silyl imine solution was used without further purification.

Cycloadduct was prepared according to the general procedure using TMAO (41 mg, 0.54 mmol, 1.4 equiv.), 0.93 M LDA (0.86 mL, 0.8 mmol, 2.0 equiv.), crude 0.50 M silyl imine (0.80 mL, 0.40 mmol, 1.0 equiv.), THF (3.0 mL, 0.10 M). The reaction was quenched with water at 0 °C and the product was extracted with DCM (x 3). The organic layer was dried over $MgSO_4$, filtered and concentrated under reduced pressure. The residue was redissolved in THF (10 mL) and 1.0 M HCl (7.0 mL, 0.7 M). The procedure was then the same as the general method for forming 1,2-diamines. Following aqueous work-up, no further purification was necessary. Product was isolated as a colorless oil. (42% yield, 28 mg, 0.17 mmol)

^{1}H NMR (400 MHz, CDCl3) δ 7.34 (dd, J = 8.6, 5.4 Hz, 2H), 7.04 (t, J = 8.6 Hz, 2H), 4.07 (dd, J = 8.0, 5.1 Hz, 1H), 2.74 (m, 2H), 2.47 (s, 3H), 1.69 (br. s, 3H).

^{13}C NMR (101 MHz, CDCl3) δ 163.2, 160.8, 140.4, 140.4, 128.1, 128.0, 115.5, 115.3, 60.2, 54.8, 36.6.

IR: 3291, 3041, 2933, 2889, 2848, 2792, 2252, 2208, 2042, 1892, 1602, 1507, 1472, 1448, 1418, 1366, 1345, 1294, 1219, 1155, 1111, 1095, 1075, 1014, 832, 754, 719, 705, 652, 634, 625, 601, 559 cm^{-1}

HRMS (ESI) m/z: $[M+CH_3OH_2]^+$ Calc'd for $C_{10}H_{18}FN_2O$ 201.1398; Found 201.1391.

N^1-methyl-2-(4-(trifluoromethyl)phenyl)ethane-1,2-diamine (4o)

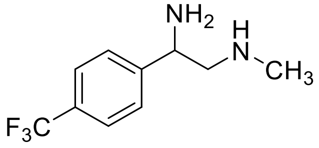

Silyl imine prepared according to the general procedure using 4-(trifluoromethyl)benzaldehyde (0.16 mL, 1.2 mmol, 1.0 equiv.), 1.9 M NaHMDS (0.63 mL, 1.2 mmol, 1.0 equiv.), and THF (1.2 mL, 1.0 M). Silyl imine solution was used without further purification.

Diamine was prepared according to the general procedure using TMAO (82 mg, 1 mmol, 1 equiv.), 1.9 M LDA (1.5 mL, 3.0 mmol, 3.0 equiv.), crude 0.66 M silyl imine (1.5 mL, 1.0 mmol, 1.0 equiv.), THF (10 mL, 0.10 M), hydroxylamine hydrochloride (377 mg, 5.0 mmol, 5.0 equiv.) and 1.2 M HCl (0.10 mL). Following aqueous work-up, no further purification was necessary. Product was isolated as a yellow oil. (32% yield, 71 mg, 0.28 mmol)

^{1}H NMR (400 MHz, $CDCl_3$) δ 7.60 (d, *J* = 8.7 Hz, 2H), 7.49 (d, *J* = 8.1 Hz, 2H), 4.17 (dd, *J* = 8.1, 5.0 Hz, 1H), 2.84 – 2.73 (m, 2H), 2.47 (s, 3H), 2.42 (br. s, 2H).

^{13}C NMR (101 MHz, CDCl3) δ 148.4 (s), 129.5 (q, *J* = 32.4 Hz), 128.2 (q, *J* = 272.7 Hz), 126.8 (s), 125.5 (q, *J* = 3.9 Hz), 122.8 (s), 120.1 (s), 59.4 (s), 54.8 (s), 36.1 (s).

IR: 3274, 2933, 2851, 2797, 2254, 2119, 1664, 1619, 1451, 1419, 1322, 1162, 1109, 1066, 1016, 955, 837, 735, 703, 652, 636, 605, 576, 561, 552, 530 cm^{-1}

HRMS (ESI) m/z: $[M+H_3O]^+$ Calc'd for $C_{10}H_{16}F_3N_2O$ 237.1209; Found 237.1204.

***N*^{1}-methyl-2-(3-(trifluoromethyl)phenyl)ethane-1,2-diamine (4p)**

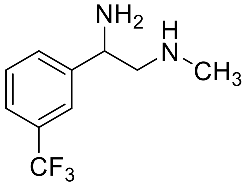

Silyl imine prepared according to the general procedure using 3-(trifluoromethyl)benzaldehyde (0.07 mL, 0.50 mmol, 1.0 equiv.), 1.8 M NaHMDS (0.29 mL, 0.50 mmol, 1.0 equiv.), and THF (0.5 mL, 1.0 M). Silyl imine solution was used without further purification.

Diamine was prepared according to the general procedure using TMAO (38 mg, 0.4 mmol, 1 equiv.), 1.4 M LDA (0.86 mL, 1.2 mmol, 3.0 equiv.), crude 0.63 M silyl imine (0.63 mL, 0.40 mmol, 1.0 equiv.), THF (3.0 mL, 0.10 M), hydroxylamine hydrochloride (140 mg, 2.0 mmol, 5.0 equiv.) and 1.2 M HCl (0.04 mL, 0.01 M). Following aqueous work-up, no further purification was necessary. Product was isolated as a yellow oil. (48% yield, 42 mg, 0.20 mmol)

^{1}H NMR (400 MHz, $CDCl_3$) δ 7.63 (ddp, *J* = 1.9, 1.3, 0.7 Hz, 1H), 7.60 – 7.51 (m, 2H), 7.49 – 7.44 (m, 1H), 4.17 (dd, *J* = 8.4, 4.8 Hz, 1H), 2.89 – 2.69 (m, 2H), 2.48 (s, 3H).

^{13}C NMR (101 MHz, $CDCl_3$) δ 145.5, 131.5 (q, *J* = 32.3 Hz), 130.1 (d, *J* = 1.0 Hz), 131.1, (d, *J* = 2.0 Hz), 129.1, 128.3 (q, *J* = 272.7 Hz), 124.4 (q, *J* = 4.04), 123.4 (d, *J* = 3.0 Hz), 123.4 (d, *J* = 4.0 Hz), 59.6, 54.9, 36.3.

IR: 3275, 2937, 2851, 2797, 1597, 1449, 1327, 1161, 1117, 1071, 1002, 899, 842, 802, 749, 703, 662, 621, 597, 560, 540, 528 cm^{-1}

HRMS (ESI) m/z: $[M+Na]^+$ $C_{10}H_{13}F_3N_2Na$ 241.0923; Found 241.0943.

***N*^{1}-methyl-2-(pyridin-2-yl)ethane-1,2-diamine (4q)**

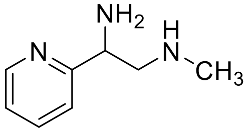

Silyl imine prepared according to the general procedure using picolinaldehyde (0.23 mL, 2.4 mmol, 1.0 equiv.), 1.5 M LiHMDS (2.4 mL, 2.4 mmol, 3.6 equiv.), and THF (2.4 mL, 1.0 M). Silyl imine solution was used without further purification.

Diamine was prepared according to the general procedure using TMAO (192 mg, 2.4 mmol, 1.0 equiv.), LDA (4.8 mL, 7.2 mmol, 3.0 equiv.), crude 0.50 M silyl imine (4.8 mL, 2.4 mmol, 1.0 equiv.), THF (10 mL, 0.10 M), hydroxylamine hydrochloride (840 mg, 12 mmol, 5.0 equiv.) and 1.2 M HCl (0.20 mL, 0.01%). No aqueous work-up was performed. Product was purified by reverse phase FCC, 0.1% TFA in water/MeOH. Isolated as an orange oil, (15% yield, 54 mg, 0.36 mmol)

^{1}H NMR (400 MHz, $CDCl_3$) δ 8.54 (d, *J* = 4.8 Hz, 1H), 7.70 (td, *J* = 7.7, 1.8 Hz, 1H), 7.34 (d, *J* = 7.8 Hz, 1H), 7.23 (ddd, *J* = 7.6, 4.8, 1.1 Hz, 1H), 5.38 (br. s, 3H), 4.33 (dd, *J* = 9.3, 4.3 Hz, 1H), 3.24 (dd, *J* = 12.2, 4.4 Hz, 1H), 3.10 (dd, *J* = 12.2, 9.4 Hz, 1H), 2.71 (s, 3H).

^{13}C NMR (101 MHz, $CDCl_3$) δ 160.5, 149.5, 137.4, 123.1, 121.8, 55.5, 53.5, 34.1.

IR: 3358, 3021, 2808, 1673, 1594, 1573, 1473, 1438, 1199, 1177, 1125, 1048, 997, 831, 799, 751, 720, 619, 597, 566, 545, 536 cm^{-1}

HRMS (ESI) m/z: $[M+H_3O]^+$ Calc'd for $C_8H_{16}N_3O$ 170.1288; Found 170.1295.

1-(furan-2-yl)-*N*[2]-methylethane-1,2-diamine (4r)

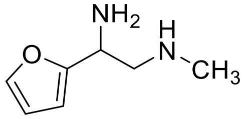

Silyl imine prepared according to the general procedure using furfural (0.22 mL, 2.6 mmol, 1.0 equiv.), 0.97 M LiHMDS (3.2 mL, 3.1 mmol, 1.2 equiv.), and THF (2.6 mL, 1.0 M). Silyl imine solution was used without further purification.

Diamine was prepared according to the general procedure using TMAO (184 mg, 2.4 mmol, 1.0 equiv.), 1.5 M LDA (4.8 mL, 7.2 mmol, 3.0 equiv.), crude 0.46 M silyl imine (2.4 mmol, 1.0 equiv.), THF (14 mL, 0.10 M), hydroxylamine hydrochloride (837 mg, 12 mmol, 5.0 equiv.) and 1.2 M HCl (0.24 mL, 0.01 M). No aqueous work-up was performed. Product was purified by reverse phase FCC, 0.1% TFA in water/MeOH. Isolated as an orange oil. (56% yield, 187 mg, 1.3 mmol)

^{1}H NMR (400 MHz, $CDCl_3$) δ 7.39 – 7.29 (m, 1H), 6.31 (dd, *J* = 3.3, 1.7 Hz, 1H), 6.19 – 6.11 (m, 1H), 4.06 (dd, *J* = 7.8, 5.1 Hz, 1H), 2.95 – 2.70 (m, 2H), 2.45 (s, 3H), 1.59 (s, 3H).

^{1}H NMR (400 MHz, $CDCl_3$) δ 7.34 (d, *J* = 0.9 Hz, 1H), 6.38 – 6.23 (m, 1H), 6.16 (d, *J* = 3.1 Hz, 1H), 4.06 (dd, *J* = 7.6, 5.2 Hz, 1H), 2.84 (ddd, *J* = 19.6, 11.8, 6.4 Hz, 2H), 2.45 (s, 3H), 1.5 (br. s, 3H).

^{13}C NMR (101 MHz, $CDCl_3$) δ 157.8, 141.6, 110.2, 104.9, 57.0, 49.4, 36.5.

IR: 3282, 2935, 2848, 2794, 1599, 1506, 1450, 1362, 1229, 1147, 1071, 1008, 912, 883, 863, 729, 620, 599, 558, 546, 539 cm^{-1}

HRMS (ESI) m/z: $[M+CH_3OH_2]^+$ Calc'd for $C_8H_{17}N_2O_2$ 173.1285; Found 173.1285.

***N*[1]-methyl-2-(quinolin-4-yl)ethane-1,2-diamine (4s)**

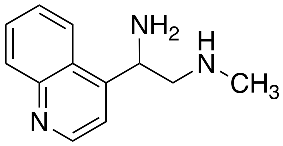

Silyl imine prepared according to the general procedure using 4-quinolinecarboxaldehyde (193 mg, 1.2 mmol, 1.0 equiv.), 1.0 M LiHMDS (1.2 mL, 1.2 mmol, 1.0 equiv.), and THF (1.2 mL, 1.0 M). Silyl imine solution was used without further purification.

Diamine was prepared according to the general procedure using TMAO (39.2 mg, 0.50 mmol, 1.0 equiv.), 0.75 M LDA (2.0 mL, 1.5 mmol, 3.0 equiv.), crude 0.50 M silyl imine (1.0 mL, 0.5 mmol, 1.0 equiv.), THF (3.0 mL, 0.10 M), 1.0M HCl (1.0 mL, 0.50 M). Following aqueous work-up, no further purification was necessary. Product was isolated as yellow oil. (76% yield, 77 mg, 0.38 mmol).

^{1}H NMR (400 MHz, $CDCl_3$) δ 8.90 (d, J = 4.5 Hz, 1H), 8.13 (d, J = 10.0 Hz, 1H), 8.10 (d, J = 10.0 Hz, 1H), 7.74 –7.68 (m, 1H), 7.61 – 7.54 (m, 2H), 4.91 (dd, J = 8.3, 3.8 Hz, 1H), 2.98 (dd, J = 12.0, 3.9 Hz, 1H), 2.77 (dd, J = 12.1, 8.3 Hz, 1H), 2.48 (s, 3H), 1.67 (br. s, 3H).

^{13}C NMR (101 MHz, $CDCl_3$) δ 150.4, 150.0, 148.3, 130.5, 129.1, 126.6, 126.3, 122.6, 117.6, 58.6, 50.1, 36.4.

IR: 3280, 3064, 2933, 2847, 2792, 2205, 1930, 1845, 1656, 1614, 1590, 1570, 1508, 1463, 1449, 1422, 1352, 1308, 1240, 1210, 1141, 1115, 1092, 1076, 1027, 953, 908, 869, 848, 814, 792, 759, 727, 642, .623, 599, 580, 556 cm^{-1}

HRMS (ESI) m/z: $[M+H]^+$ Calc'd for $C_{12}H_{16}N_3$ 202.1339; Found 202.1335.

4-(1-methylimidazolidin-4-yl)pyridine (4t)

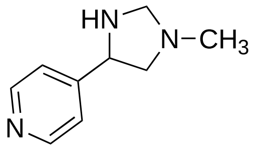

Silyl imine prepared according to the general procedure using 4-pyridylcarboxaldehyde (0.10 mL, 1.1 mmol, 1.0 equiv.), 1.0 M LiHMDS (1.1 mL, 1.1 mmol, 1.0 equiv.), and THF (1.1 mL, 1.0 M). Silyl imine solution was used without further purification.

Imidazolidine was prepared according to the general procedure using TMAO (39 mg, 0.52 mmol, 1.2 equiv.), 0.75 M LDA (2.0 mL, 1.5 mmol, 3.0 equiv.), crude 0.50 M silyl imine (1.0 mL, 0.50 mmol, 1.0 equiv.), THF (3.0 mL, 0.10 M), and 1.0 M HCl (1.0 mL, 0.01 M). Following aqueous work-up, no further purification was necessary. Product was isolated as an orange oil. (85% yield, 69 mg, 0.43 mmol)

^{1}H NMR (400 MHz, $CDCl_3$) δ 8.53 (dd, J = 4.4, 1.5 Hz, 2H), 7.31 (dd, J = 6.1, 0.6 Hz, 2H), 4.48 (t, J = 7.6 Hz, 1H), 3.82 (d, J = 7.3 Hz, 1H), 3.44 (d, J = 7.3 Hz, 1H), 3.28 (dd, J = 9.6, 7.6 Hz, 1H), 2.38 (s, 3H), 2.34 (d, J = 2.1 Hz, 1H).

^{13}C NMR (101 MHz, $CDCl_3$) δ 153.3, 149.9, 121.5, 73.0, 62.4, 59.9, 39.4.

IR: 3255, 3027, 2941, 2847, 1788, 2251, 1941, 1666, 1597, 1557, 1491, 1451, 1411, 1363, 1308, 1219, 1151, 1125, 1063, 992, 895, 820, 732, 699, 668, 630, 622, 608, 584, 573 cm^{-1}

HRMS (ESI) m/z (z=2): $[M+2Na]^{+2}$ Calc'd for $C_9H_{13}N_3Na_2$ 104.5447; Found 104.5440.

_N,N_-dimethylaniline-_N_-oxide (SI-3)

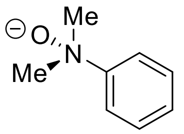

Dimethylaniline (1.26 mL, 10 mmol, 1.0 equiv.) was added to a round-bottom flask and dissolved in chloroform (10 mL, 1.0 M). The reaction was cooled to 0 °C in an ice bath, and 75% *meta*-chloroperoxybenzoic acid (2.36 g, 10 mmol, 1.0 equiv.) was added portion-wise over a period of 10 min. After the addition was complete, the reaction was warmed to RT and let to stir for 1 h. Potassium carbonate was then added and the reaction was stirred vigorously for 1 h. Solids were filtered off and the filtrate was dried over sodium sulfate. The sodium sulfate was filtered off and the filtrate was concentrated resulting in the pure *N*-oxide as a yellow solid. (91% yield,1.243 g, 9.1 mmol).

The^{1}H NMR matched the spectrum reported by Chain and coworkers.[12]

^{1}H NMR (400 MHz, $CDCl_3$) δ 7.50 (dd, J = 8.6, 1.4 Hz, 2H), 6.92 (dd, J = 8.6, 7.0 Hz, 2H), 6.88 – 6.81 (m, 1H), 3.06 (s, 6H).

3,3-dimethyl-_N_^{1}-phenylbutane-1,2-diamine (4u)

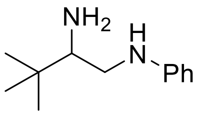

Silyl imine prepared according to the general procedure using pivaldehyde (65 µL, 0.6 mmol, 1.0 equiv.), 0.81 M NaHMDS (0.81 mL, 0.66 mmol, 1.1 equiv.), and THF (0.6 mL, 1.0 M). Silyl imine solution was used without further purification.

Diamine was prepared according to the general procedure using *N,N*-dimethylaniline-*N*-oxide (75.0 mg, 0.55 mmol, 1.0 equiv.), 1.6 M LDA (1.1 mL, 1.7 mmol, 3.0 equiv.), crude 0.43 M silyl imine (1.3 mL, 0.55 mmol, 1.0 equiv.), THF (3.1 mL, 0.10 M), hydroxylamine hydrochloride (191 mg, 2.8 mmol, 5.0 equiv.) and 1.2 M HCl (0.05 mL, 0.01 M). The resulting mixture was purified by FCC (9:9:1 Hexanes:EtOAc:NEt_3 to 19:1 EtOAc:NEt_3) to give compound **4u** as an off-white solid. (69% yield, 73 mg, 0.38 mmol)

^{1}H NMR (400 MHz, CDCl3) δ 7.16 (t, *J* = 7.5 Hz, 2H), 6.72 (t, *J* = 7.2 Hz, 1H), 6.66 (d, *J* = 8.6 Hz, 2H), 3.46 (dd, *J* = 12.6, 2.2 Hz, 1H), 2.98 (dd, *J* = 12.7, 10.1 Hz, 1H), 2.84 – 2.78 (m, 1H), 1.05 (s, 9H).

^{13}C NMR (101 MHz, CDCl3) δ 147.9, 129.3, 117.5, 113.1, 60.3, 44.2, 33.4, 26.4.

IR: 3313 3023 2956 2923 2868 2161 1730 1602 1503 1476 1405 1366 1330 1257 1179 1153 1096 1070 1028 991 867 806 747 692 511 463 430 413 406 cm^{-1}

HRMS (ESI) m/z: $[M+H]^+$ Calc'd for $C_{12}H_{21}N_2$ 193.1699; Found 193.1683.

1-(4-methoxyphenyl)-_N_^{2}-phenylethane-1,2-diamine (4v)

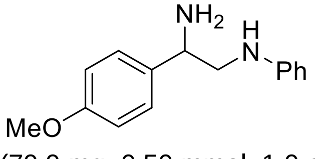

Silyl imine prepared according to the general procedure using *p*-anisaldehyde (0.07 mL, 0.6 mmol, 1.0 equiv.), 0.81 M NaHMDS (0.88 mL, 0.72 mmol, 1.2 equiv.), and THF (0.6 mL, 1.0 M). Silyl imine solution was used without further purification.

Diamine was prepared according to the general procedure using *N,N*-dimethylaniline-*N*-oxide (70.0 mg, 0.50 mmol, 1.0 equiv.), 1.6 M LDA (0.95 mL, 1.5 mmol, 3.0 equiv.), crude 0.40 M silyl imine (1.2 mL, 0.5 mmol, 1.0 equiv.), THF (2.8 mL, 0.10 M), hydroxylamine hydrochloride (140 mg, 2.0 mmol, 5.0 equiv.) and 1.2 M HCl (0.04 mL, 0.01 M). The resulting mixture was purified by FCC (9:9:1 Hexanes:EtOAc:NEt_3 to 19:1 EtOAc:NEt_3) to give compound **4v** as a clear oil. (52% yield, 63 mg, 0.26 mmol)

^1H NMR (400 MHz, CDCl3) δ 7.29 (d, J = 8.7 Hz, 2H), 7.20 – 7.13 (d, J = 8.0 Hz, 2H), 6.90 (d, J = 8.7 Hz, 2H), 6.71 (t, J = 7.3 Hz, 1H), 6.64 (d, J = 7.6 Hz, 2H), 4.15 (dd, J = 8.2, 5.3 Hz, 1H), 3.81 (s, 3H), 3.32 (dd, J = 12.6, 5.1 Hz, 1H), 3.22 (dd, J = 12.7, 8.3 Hz, 1H), 1.84 (br. s, 3H).

^{13}C NMR (101 MHz, CDCl3) δ 158.9, 148.0, 136.0, 129.3, 127.4, 117.5, 114.0, 113.0, 55.3, 54.3, 51.8.

IR: 3292, 2999, 2916, 2835, 1891, 1602, 1584, 1509, 1463, 1441, 1303, 1245, 1202, 1177, 1109, 1071, 1032, 991, 909, 870, 831, 750, 733, 694, 639, 550, 511, 447, 423, 415 cm^{-1}

HRMS (ESI) m/z: $[M+H]^+$ Calc'd for $C_{15}H_{19}N_2O^+$ 243.1492; Found 243.1492.

Procedure for the di-tosylation of 4a (5)

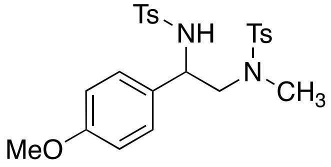

Under ambient conditions, a RBF flask was charged with **4a** (31mg, 0.17 mmol, 1.0 equiv.), diisopropylethylamine (0.073 mL, 0.43 mmol, 2.5 equiv.) and DCM (1.7 mL, 0.1M). *p*-Toluenesulfonylchloride (67 mg, 0.35 mmol, 2.05 equiv.) was added all at once and the solution was stirred at RT while being monitored by TLC until consumption of starting material. After 2h, the organic layer was washed with DI H_2O and the aqueous layers were rinsed with DCM (x2). The organic layers were combined, dried with $MgSO_4$, and concentrated *in vacu*. The compound was purified *via* flash column chromatography (30% EtOAc in hexanes) to afford a white solid. (96% yield, 78 mg 0.16 mmol) (Rf = 0.77 in 1:1 Hex/EtOAc)

^1H NMR (400 MHz, $CDCl_3$) δ 7.71 (d, J = 8.3 Hz, 2H), 7.60 (d, J = 8.3 Hz, 2H), 7.31 – 7.25 (m, 4H), 7.14 (d, J = 8.8 Hz, 2H), 6.79 (d, J = 8.7 Hz, 2H), 5.59 (d, J = 4.2 Hz, 1H), 4.33 (dt, J = 9.2, 4.4 Hz, 1H), 3.79 (s, 3H), 3.41 (dd, J = 14.4, 9.5 Hz, 1H), 2.70 (dd, J = 14.4, 4.8 Hz, 1H), 2.43 (s, 6H), 2.42 (s, 3H).

^{13}C NMR (126 MHz, $CDCl_3$) δ 159.4, 143.9, 143.2, 136.9, 134.0, 130.2, 129.9, 129.5, 128.1, 127.5, 127.3, 114.0, 55.7, 55.3, 55.0, 35.6, 21.6, 21.5.

IR: 3281, 2920, 2850, 1612, 1598, 1514, 1495, 1456, 1331, 1305, 1247, 1212, 1180, 1157, 1120, 1090, 1032, 944, 867, 831, 814, 780, 736, 720, 705, 666 cm^{-1}

HRMS (ESI) m/z (z=2): $[M+2H]^{+2}$ Calc'd for $C_{24}H_{30}N_2O_5S_2$ 245.0793; Found 245.0784.

Procedure for the mono-tosylation of 4a (6)

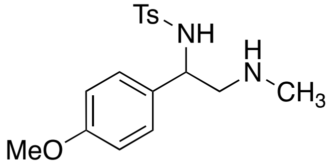

Under ambient conditions, a RBF flask was charged with **4a** (33mg, 0.18 mmol, 1.0 equiv.), diisopropylethylamine (0.061 mL. 0.36 mmol, 2.0 equiv.) and DCM (3.4 mL, 0.1M). The solution was cooled to -78 ˚C while *p*-tolulenesulfonylchloride (34mg, 0.18 mmol 1.0 equiv.) was dissolved in DCM (0.5 mL) in a separate flask. The sulfonylchloride/DCM suspension was added dropwise to the **4a** solution, *via* syringe, over the course of 10 minutes at -78 ˚C. After addition, the solution was allowed to slowly warm to room temperature and was monitored by TLC until consumption of starting material. After 2h, the organic layer was washed with DI H_2O and the aqueous layers were rinsed with DCM (x2). The organic layers were combined, dried with $MgSO_4$, and concentrated *in vacu*. The compound was purified *via* flash column chromatography (5% MeOH in DCM) to afford a white solid. (72% yield, 43 mg, 0.13 mmol.) (Rf = 0.54 in 10:1 DCM/MeOH)

^1H NMR (400 MHz, $CDCl_3$) δ 7.67 (d, J = 8.3 Hz, 2H), 7.31 (d, J = 8.6 Hz, 4H), 6.89 (d, J = 8.7 Hz, 2H), 4.18 (dd, J = 9.2, 4.5 Hz, 1H), 3.82 (s, 3H), 3.29 (dd, J = 13.4, 9.1 Hz, 1H), 2.81 (dd, J = 13.4, 4.5 Hz, 1H), 2.74 (s, 3H), 2.43 (s, 3H), 1.72 (br. s, 2H).

^{13}C NMR (101 MHz, $CDCl_3$) δ 159.1, 143.5, 134.7, 134.2, 129.7, 127.8, 127.5, 114.0, 58.9, 55.3, 53.7, 36.4, 21.5.

IR: 2921, 1739, 1611, 1512, 1456, 1338, 1304, 1247, 1217, 1180, 1160, 1119, 1089, 1033, 949, 833, 816, 775, 753, 720, 703, 653, 583 cm^{-1}

HRMS (ESI) m/z (z=2): $[M+2H]^{+2}$ Calc'd for $C_{17}H_{24}N_2O_3S$ 168.0748; Found 168.0735.

Procedure for the mono-nosylation of 4a (7)

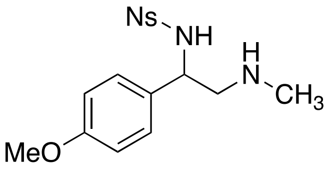

Under ambient conditions, a RBF flask was charged with **4a** (54 mg, 0.3 mmol, 1.0 equiv.), triethylamine (0.080 mL, 0.6 mmol, 2.0 equiv.) and DCM (3.0 mL, 0.1M). The solution was cooled to -78 °C while 2-nitrobenzenesulfonylchloride (66 mg, 0.3 mmol, 1.0 equiv.) was dissolved in DCM (0.5 mL) in a separate flask. The sulfonylchloride/DCM suspension was added dropwise to the **4a** solution, *via* syringe, over the course of 10 minutes at -78 °C. After addition, the solution was allowed to slowly warm to room temperature and was monitored by TLC until consumption of starting material. After 2h, the organic layer was washed with DI H_2O and the aqueous layers were rinsed with DCM (x2). The organic layers were combined, dried with $MgSO_4$, and concentrated *in vacu*. The compound was purified *via* flash column chromatography (5% MeOH in DCM) to afford a light yellow amorphous solid. (75% yield, 82 mg 0.23 mmol) (Rf = 0.60 in 10:1 DCM/MeOH)

^{1}H NMR (400 MHz, $CDCl_3$) δ 7.97 (dd, J = 7.2, 2.1 Hz, 1H), 7.69 (td, J = 7.0, 1.7 Hz, 2H), 7.62 (dd, J = 7.6, 1.9 Hz, 1H), 7.31 (d, J = 8.8 Hz, 2H), 6.89 (d, J = 8.7 Hz, 2H), 4.21 (dd, J = 8.9, 5.0 Hz, 1H), 3.82 (s, 3H), 3.52 (dd, J = 14.1, 8.9 Hz, 1H), 3.17 (dd, J = 14.1, 5.0 Hz, 1H), 2.89 (s, 3H), 1.67 (s, 2H).

^{13}C NMR (126 MHz, $CDCl_3$) δ 159.1, 148.3, 134.6, 133.6, 132.0, 131.6, 131.0, 127.8, 124.1, 114.1, 58.5, 55.3, 53.7, 35.8.

IR: 3378, 3095, 2913, 2838, 1611, 1586, 1541, 1511, 1464, 1440, 1372, 1343, 1303, 1246, 1160, 1124, 1059, 1030, 955, 852, 832, 769, 730, 701, 651, 638, 614, 575 cm^{-1}

HRMS (ESI) m/z: $[M+Na]^+$ Calc'd for $C_{16}H_{19}N_3O_5SNa$ 388.0938; Found 388.0970.

Procedure for the pivaloyl mono-functional of 4a (8)

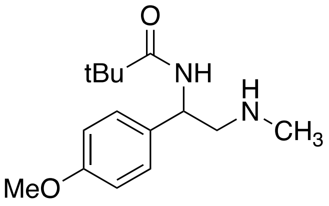

Under ambient conditions, a RBF flask was charged with **4a** (50 mg, 0.28 mmol, 1.0 equiv.), triethylamine (0.074 mL, 0.56 mmol, 2.0 equiv.) and DCM (2.8 mL, 0.1M). The solution was cooled to -78 °C while 2,2-dimethylpropanoyl chloride (pivaloyl chloride) (0.034 mL, 0.28 mmol, 1.0 equiv.) was dissolved in DCM (0.5 mL) in a separate flask. The pivaloyl chloride/DCM suspension was added dropwise to the **4a** solution, *via* syringe, over the course of 10 minutes at -78 °C. After addition, the solution was allowed to slowly warm to room temperature and was monitored by TLC until consumption of starting material. After 2h, the organic layer was washed with DI H_2O and the aqueous layers were rinsed with DCM (x2). The organic layers were combined, dried with $MgSO_4$, and concentrated *in vacu*. The compound was purified *via* flash column chromatography (35% MeOH in DCM) to afford a yellow oil. (60% yield, 44 mg, 0.17 mmol) (Rf = 0.45 in 10:1 DCM/MeOH)

^{1}H NMR (400 MHz, MeOD) δ 7.28 (d, J = 8.8 Hz, 2H), 6.92 (d, J = 8.7 Hz, 2H), 5.13 (dd, J = 8.9, 5.4 Hz, 1H), 3.79 (s, 3H), 3.13 (dd, J = 12.7, 9.1 Hz, 1H), 2.99 (dd, J = 12.7, 5.4 Hz, 1H), 2.51 (s, 3H), 1.22 (s, 9H).

^{13}C NMR (126 MHz, $CDCl_3$) δ 178.6, 159.0, 127.4, 114.2, 55.4, 55.3, 51.3, 38.8, 35.4, 27.6.

IR: 3332, 2958, 2871, 2837, 2802, 1888, 1639, 1613, 1586, 1511, 1480, 1463, 1399, 1366, 1302, 1276, 1246, 1214, 1178, 1118, 1032, 982, 912, 829, 730, 682, 645, 616, 575, 550, 542, 532 cm^{-1}

HRMS (ESI) m/z: $[M+Na]^+$ Calc'd for $C_{15}H_{24}N_2O_2Na$ 287.1730; Found 287.1748.

Synthesis of 4-(4-methoxyphenyl)-1-methylimidazolidin-2-one (9)

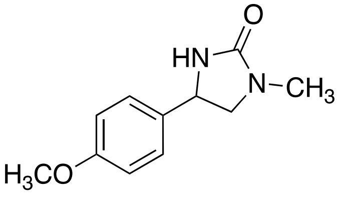

Under ambient conditions, a RBF flask was charged with **4a** (65 mg, 0.36 mmol, 1.0 equiv.), triethylamine (0.14 mL, 1.10 mmol, 3.0 equiv.) and DCM (1.80 mL, 0.2M). The solution was cooled to 0 °C. Once cooled, triphosgene (128 mg, 0.43 mmol, 1.2 equiv.) was added all at once. After addition, the solution was allowed to slowly warm to room temperature and was monitored by TLC until consumption of starting material. After 1.5h, the solution was quenched with saturated sodium bicarbonate, and the aqueous layers were rinsed with DCM (x2). The organic layers were combined, dried with $MgSO_4$, and concentrated *in vacu*. The compound was purified *via* flash column chromatography (5% MeOH in DCM) to afford an off-white solid. (43% yield, 32 mg 0.15 mmol) (Rf = 0.69 in 10:1 DCM/MeOH)

^1H NMR (400 MHz, $CDCl_3$) δ 7.30 (d, J = 8.8 Hz, 2H), 6.92 (d, J = 8.8 Hz, 2H), 4.71 (t, J = 8.3 Hz, 2H), 3.83 (s, 3H), 3.75 (d, J = 8.9 Hz, 1H), 3.20 (d, J = 8.8 Hz, 1H), 2.84 (s, 3H).

^{13}C NMR (126 MHz, $CDCl_3$) δ 162.2, 159.5, 133.4, 127.4, 114.2, 56.3, 55.4, 53.2, 30.6.

IR: 3279, 2919, 2850, 2054, 1694, 1611, 1586, 1512, 1441, 1401, 1361, 1293, 1244, 1175, 1112, 1090, 1029, 954, 831, 813, 759, 662 cm^{-1}

HRMS (ESI) m/z (z=2): [M+2H]+2 Calc'd for $C_{11}H_{16}N_2O_2$ 104.0600; Found 104.0591.

Synthesis of 4-(4-methoxyphenyl)-1-methyl-2-phenyl-4,5-dihydro-1*H*-imidazole (10)[13]

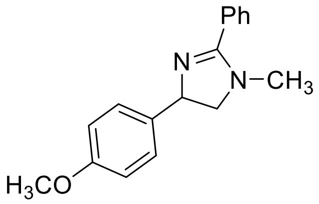

Diamine **4a** (323 mg, 1.8 mmol, 1.0 equiv.) was dissolved in 10 mL of methanol and ethyl benzimidate hydrochloride (415 mg, 2.2 mmol, 1.2 equiv.) was added. The reaction mixture was stirred at RT for 10 h and then volatiles were removed *in vacuo*. Residue was purified by flash column chromatography with a DCM/MeOH gradient to afford an off-white amorphous solid. (62% yield, 165 mg, 1.1 mmol). (Rf = 0.27 in 9:1 DCM/MeOH).

^1H NMR (400 MHz, $CDCl_3$) δ 7.74 (d, 2H), 7.54 (t, *J* = 7.5 Hz, 1H), 7.44 (t, *J* = 7.7 Hz, 2H), 7.27 (d, *J* = 8.6 Hz, 2H), 6.82 (d, *J* = 8.7 Hz, 2H), 5.26 (t, *J* = 10.0 Hz, 1H), 4.51 (t, *J* = 11.6 Hz, 1H), 3.72 (s, 3H), 3.69 (d, *J* = 8.3 Hz, 1H), 3.11 (s, 3H).

^{13}C NMR (126 MHz, $CDCl_3$) δ 165.7, 159.6, 133.4, 131.3, 129.5, 129.2, 127.7, 122.4, 114.5, 60.6, 58.3, 55.4, 34.9.

IR: 2836, 1603, 1560, 1514, 1491, 1445, 1376, 1246, 1178, 1095, 1027, 926, 832, 777, 725, 699, 616, 560, 545, 539 cm^{-1}

HRMS (ESI) m/z: $[M+H_3O]^+$ Calc'd for $C_{17}H_{21}N_2O_2$ 285.1598; Found 285.1575.

^{1}H NMR spectrum of **4a** (In $CDCl_3$, 400 MHz)

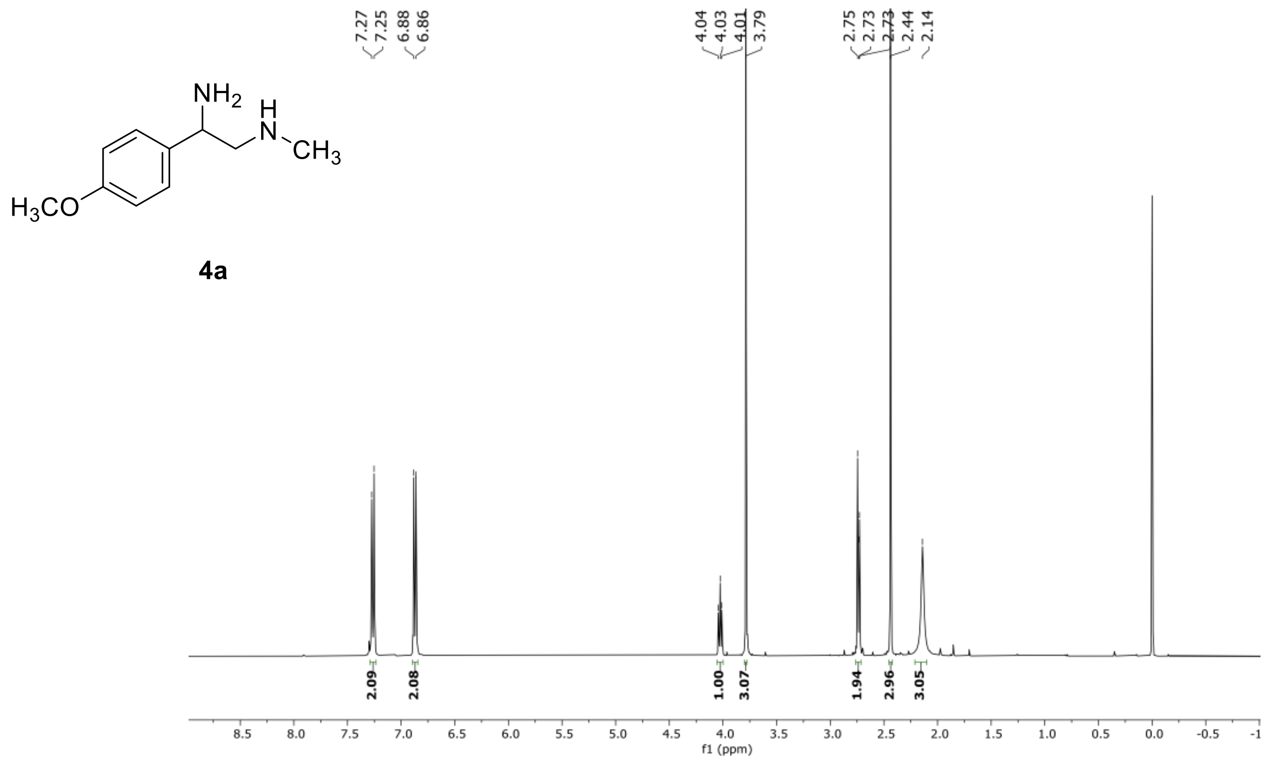

^{13}C NMR spectrum of **4a** (In $CDCl_3$, 101 MHz)

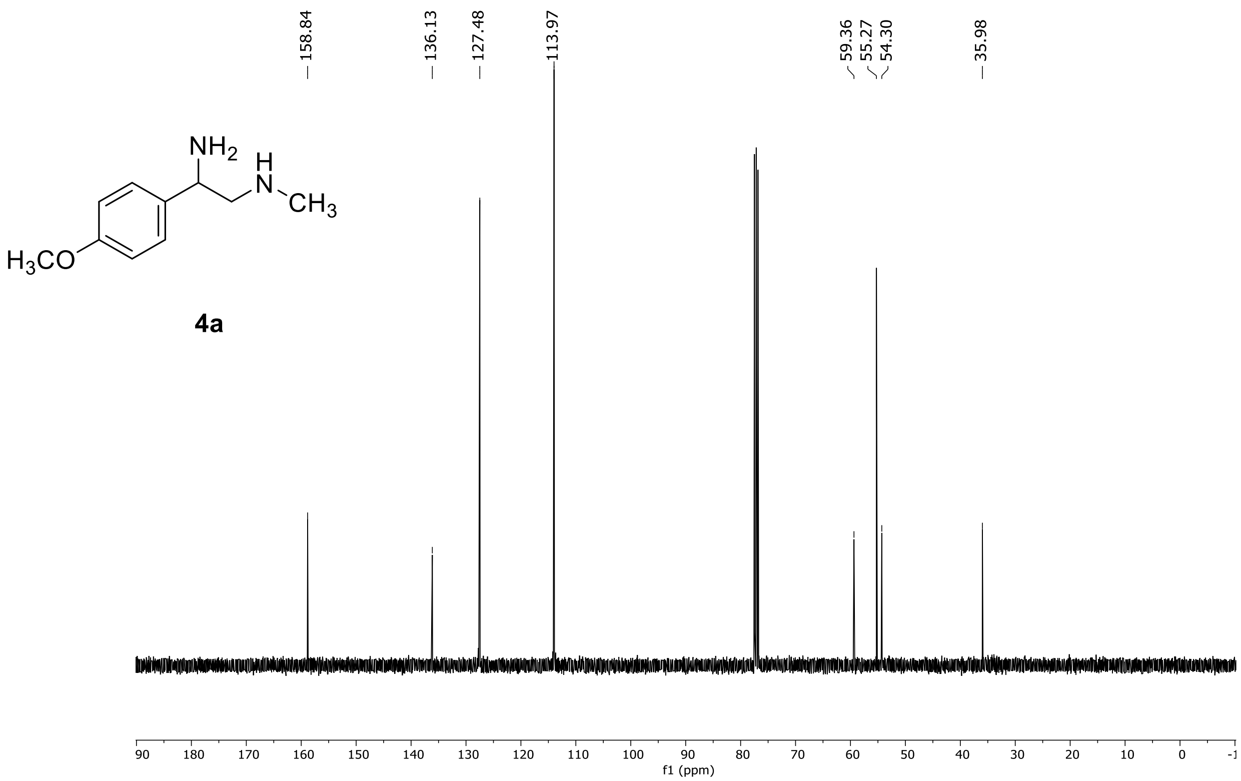

^{1}H NMR spectrum of **4b** (In $CDCl_3$, 400 MHz)

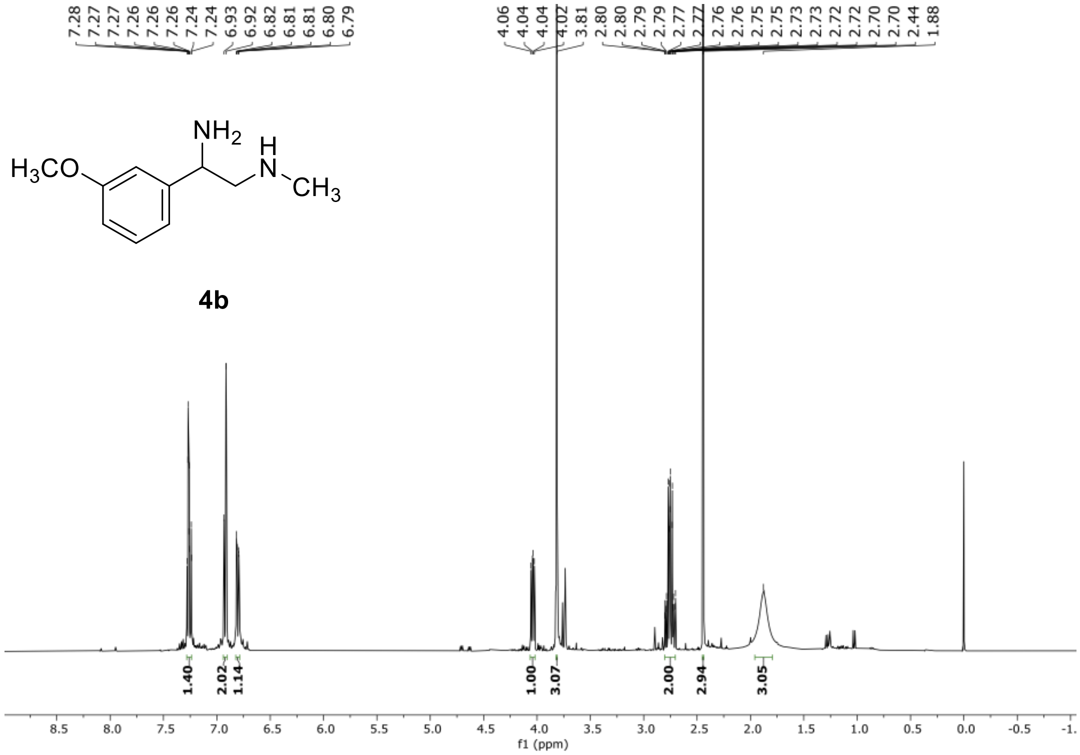

^{13}C NMR spectrum of **4b** (In $CDCl_3$, 101 MHz)

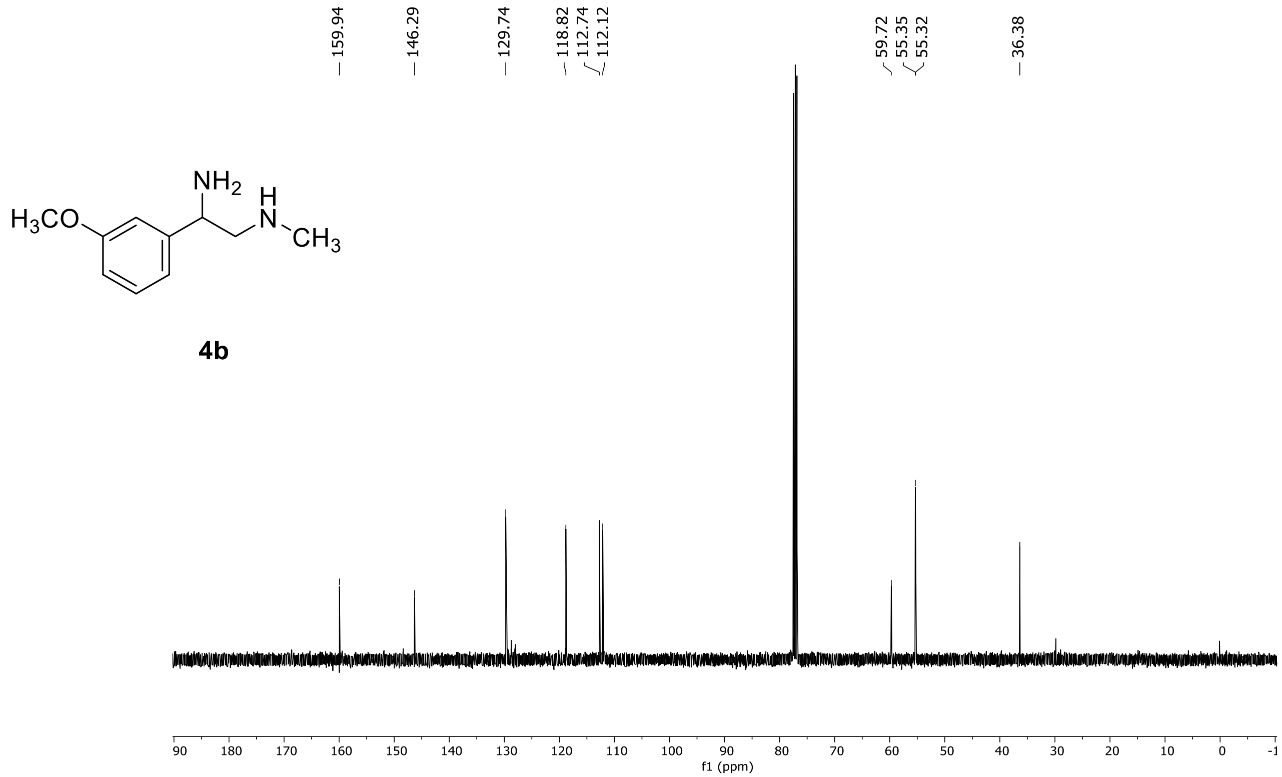

^{1}H NMR spectrum of **4c** (In $CDCl_3$, 400 MHz)

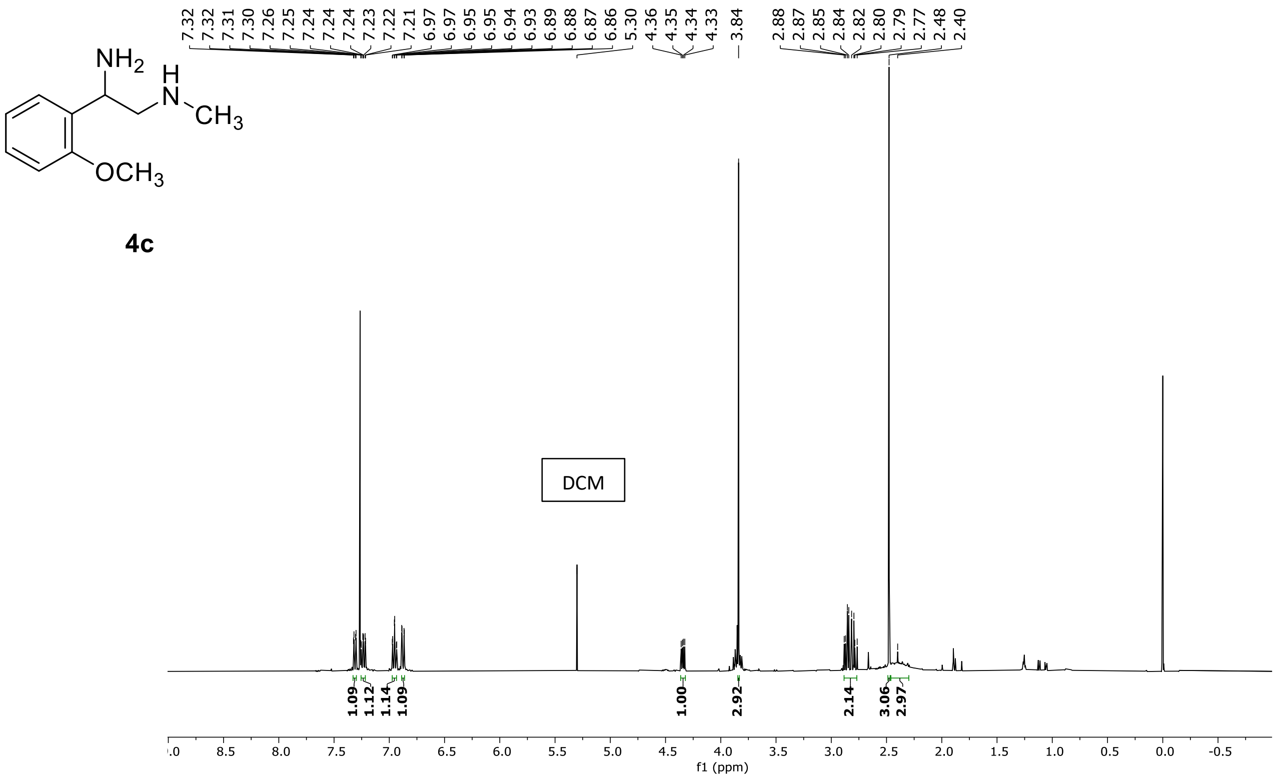

^{13}C NMR spectrum of **4c** (In $CDCl_3$, 101 MHz)

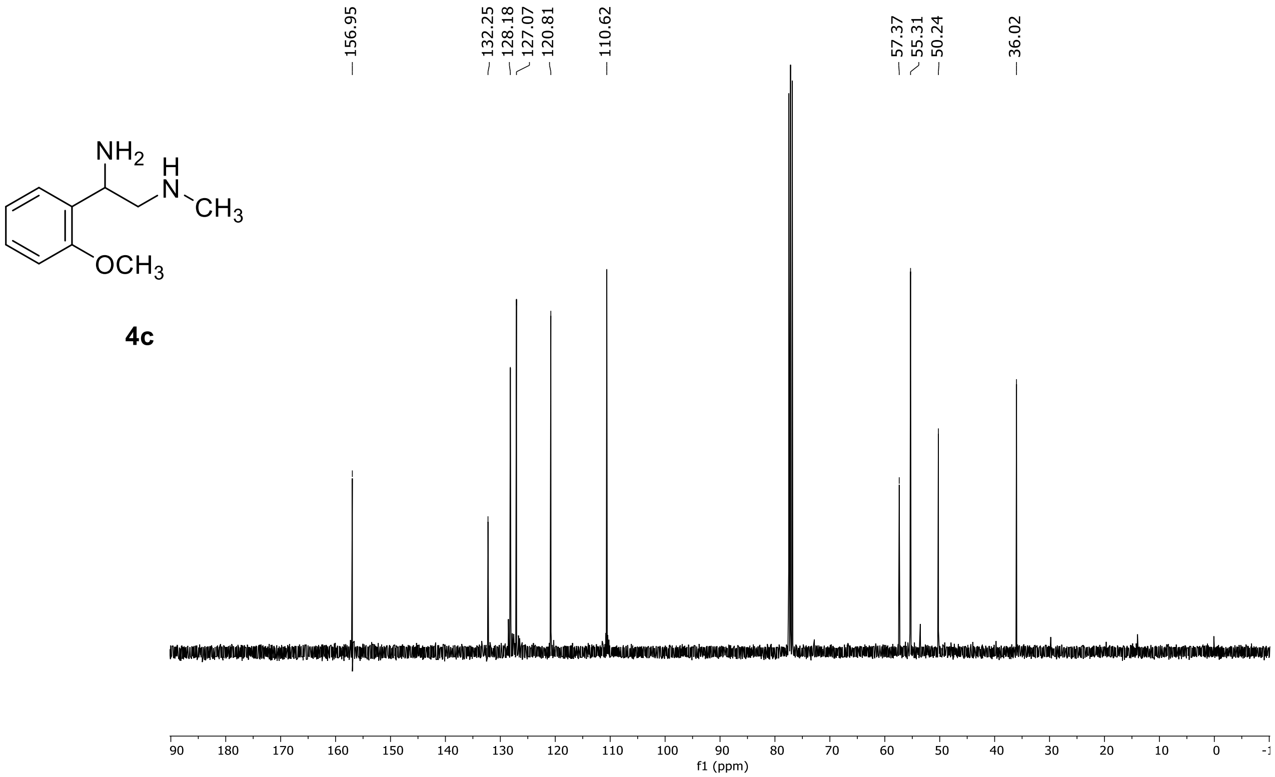

^{1}H NMR spectrum of **4d** (In $CDCl_3$, 400 MHz)

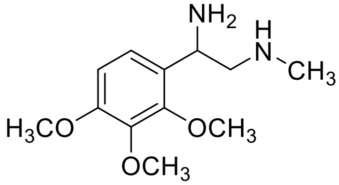

4d

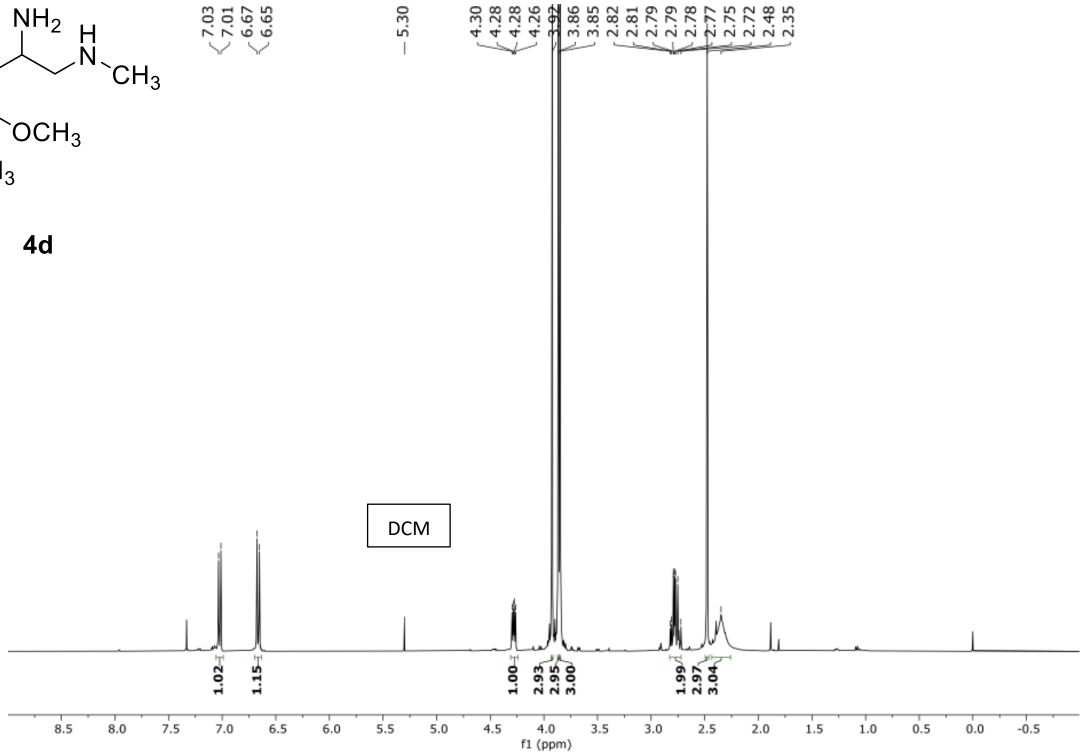

^{13}C NMR spectrum of **4d** (In $CDCl_3$, 126 MHz)

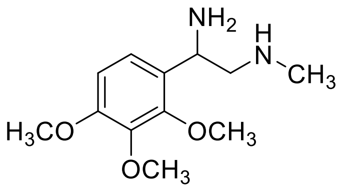

4d

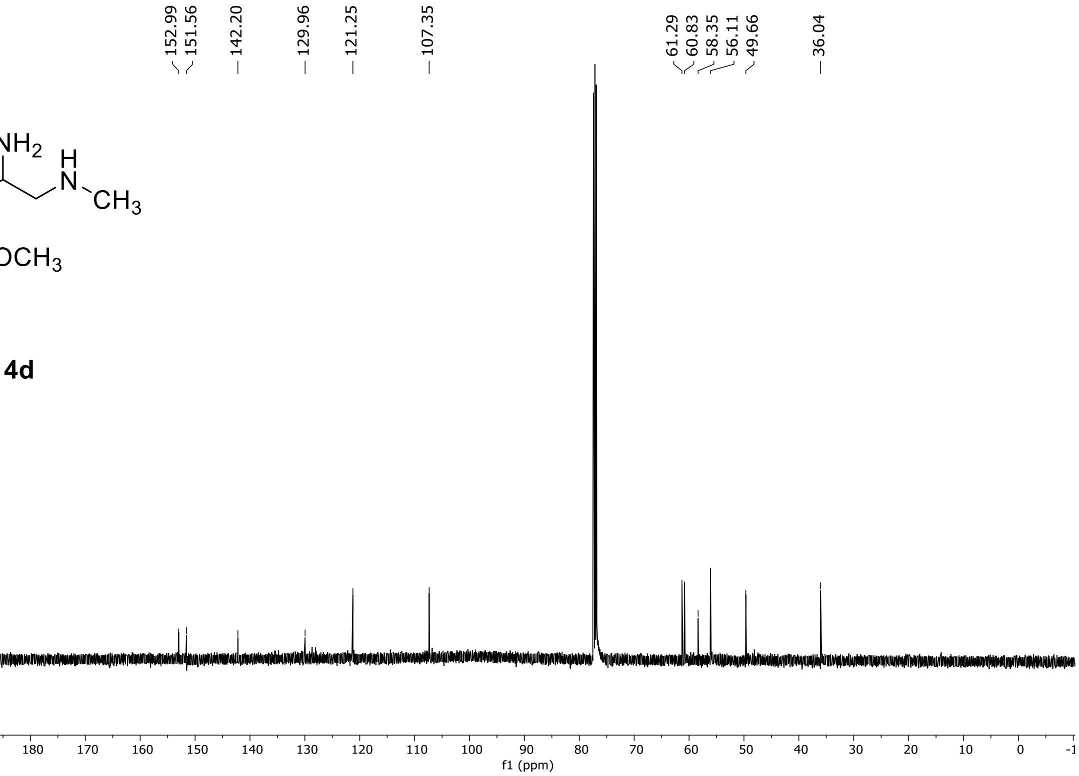

^{1}H NMR spectrum of **4e** (In $CDCl_3$, 400 MHz)

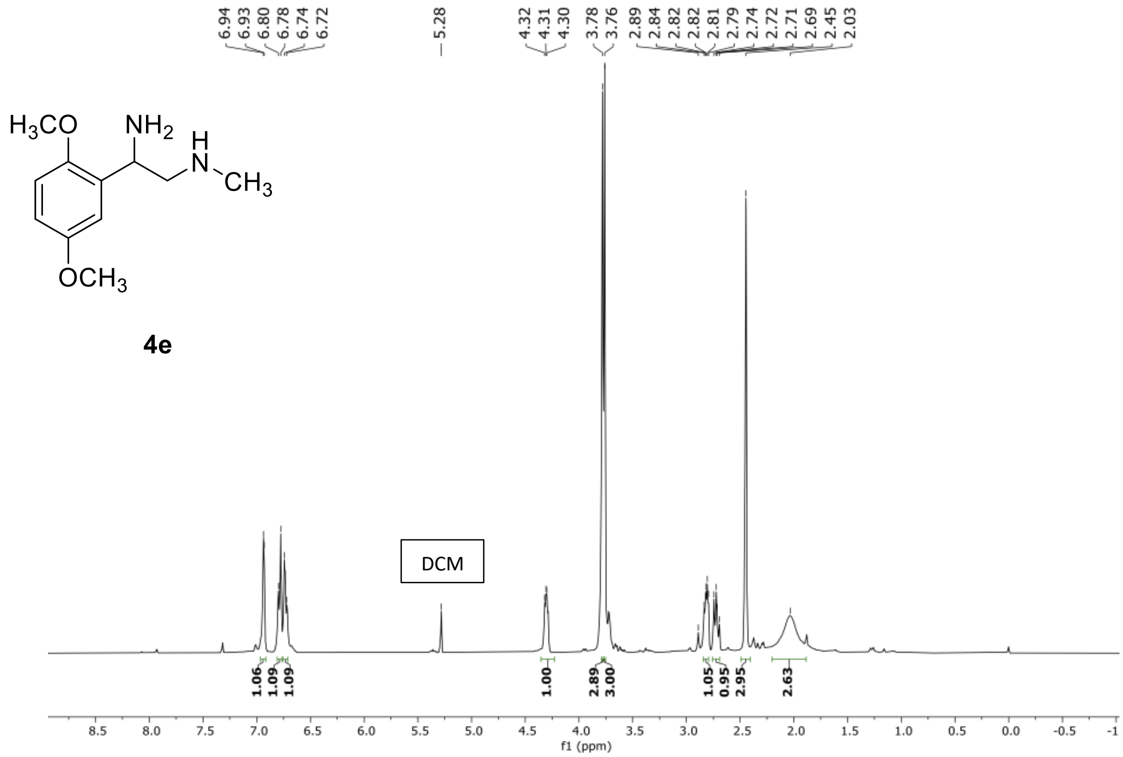

^{13}C NMR spectrum of **4e** (In $CDCl_3$, 101 MHz)

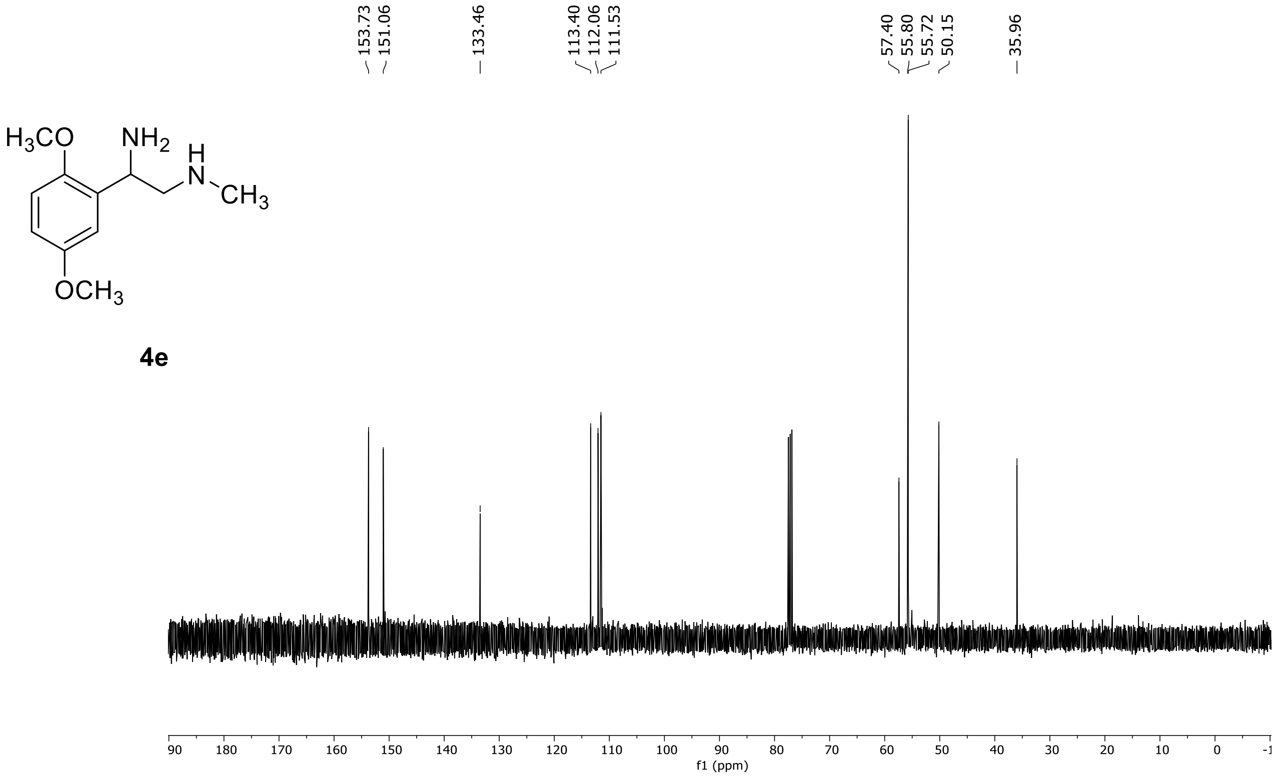

^1H NMR spectrum of **4f**(In $CDCl_3$, 400 MHz)

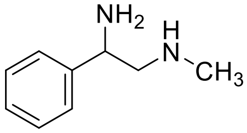

4f

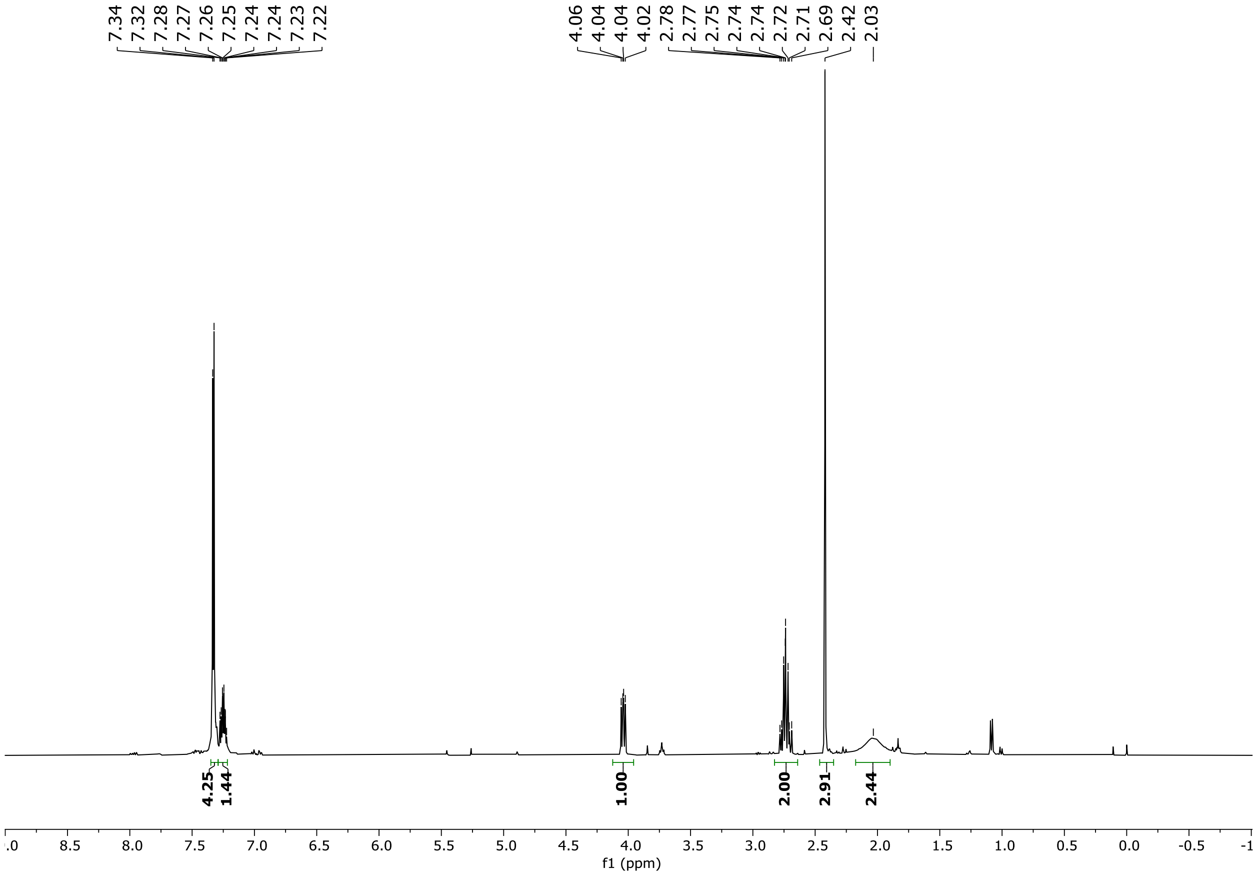

^{1}H NMR spectrum of **4g** (In $CDCl_3$, 400 MHz)

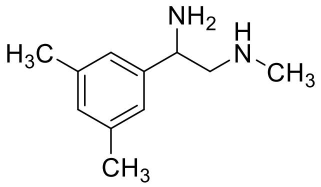

4g

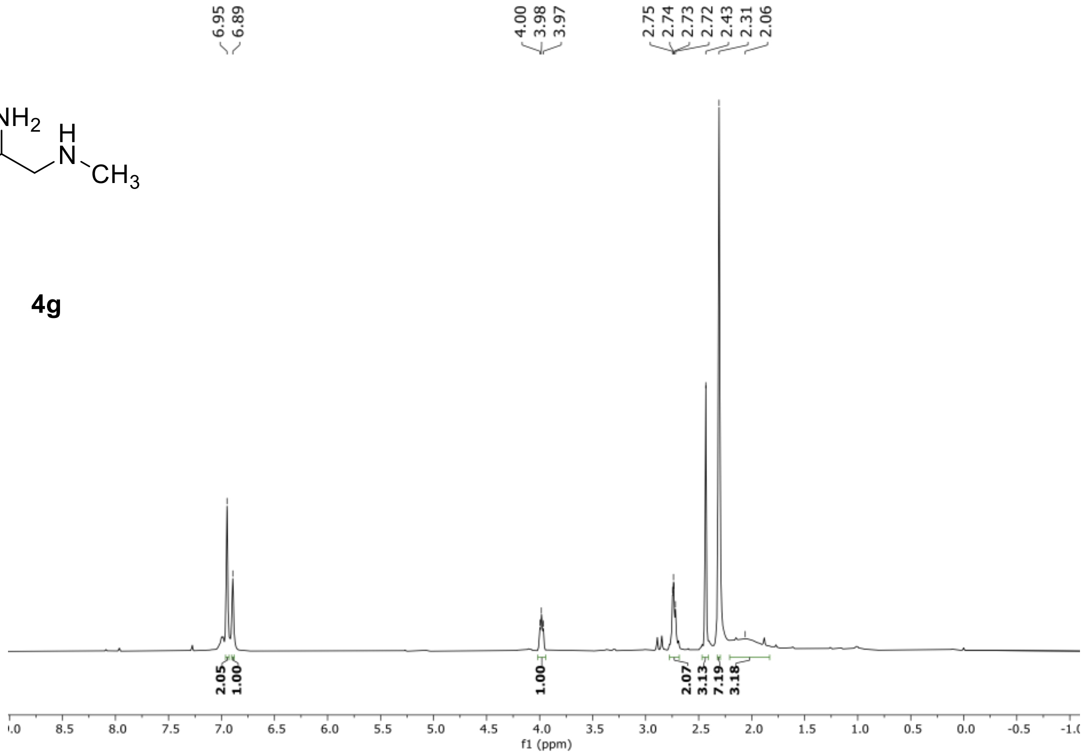

^{13}C NMR spectrum of **4g** (In $CDCl_3$, 101 MHz)

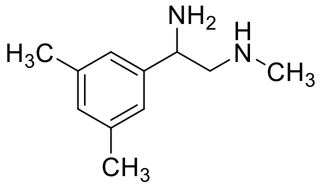

4g

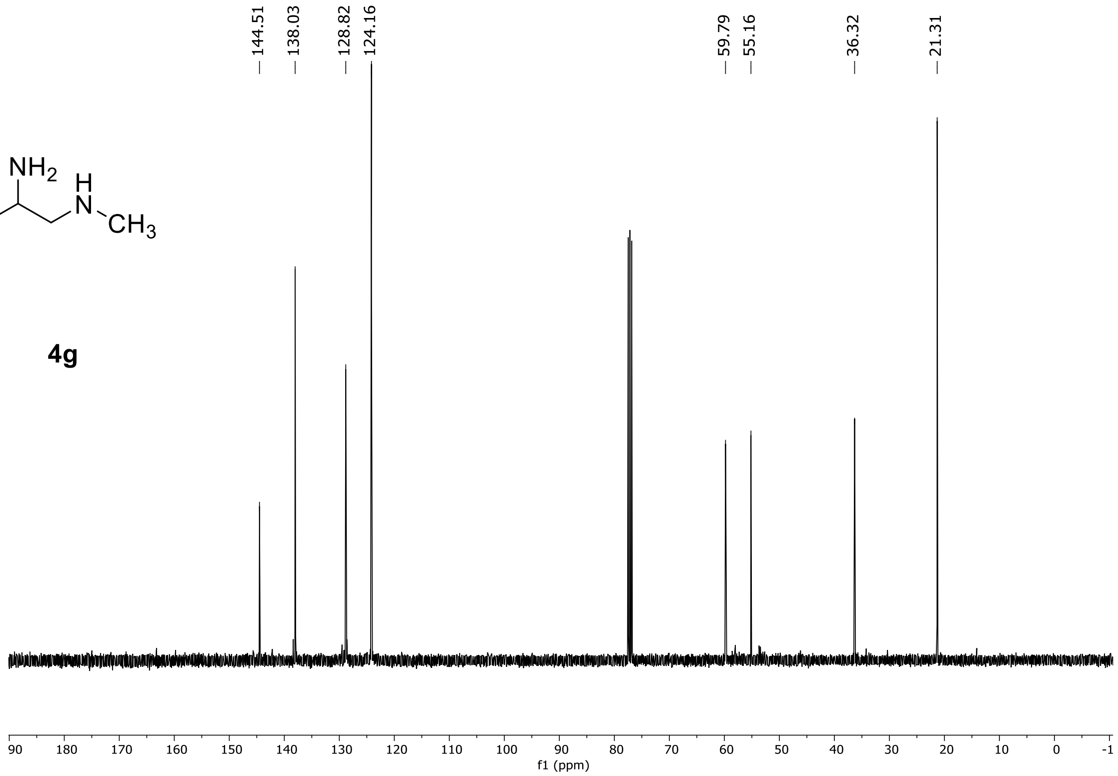

^{1}H NMR spectrum of **4h** (In $CDCl_3$, 400 MHz)

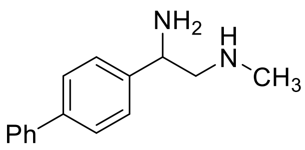

4h

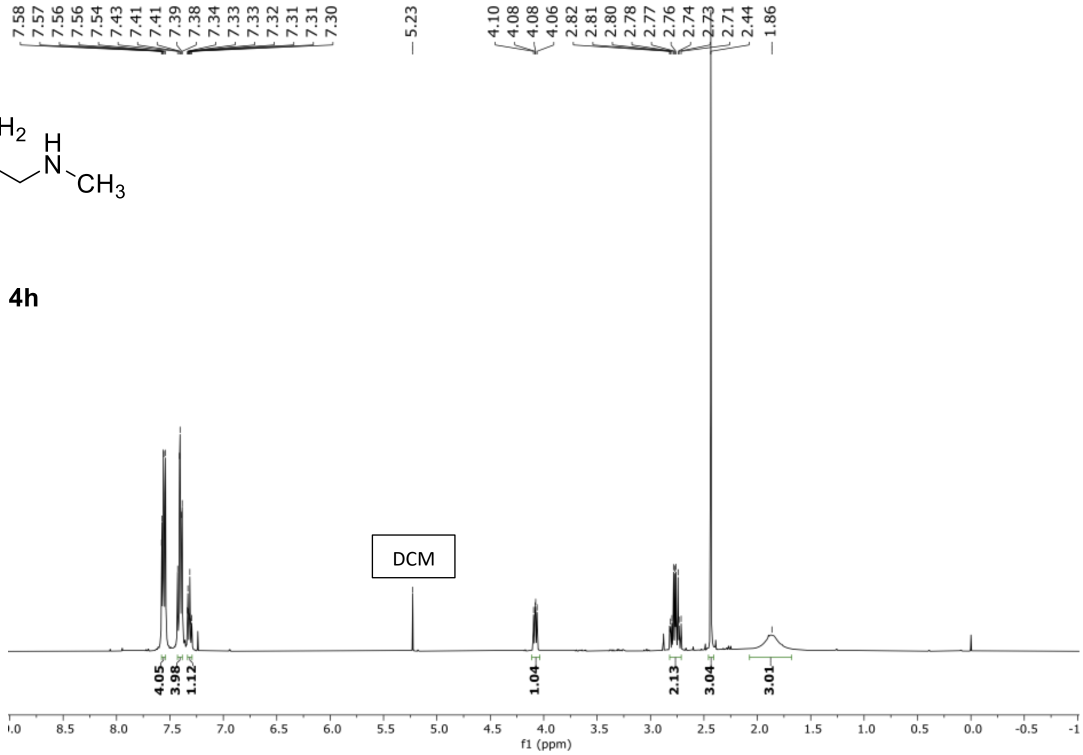

^{13}C NMR spectrum of **4h** (In $CDCl_3$, 101 MHz)

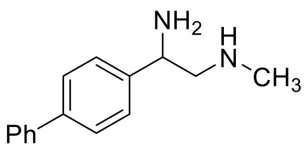

4h

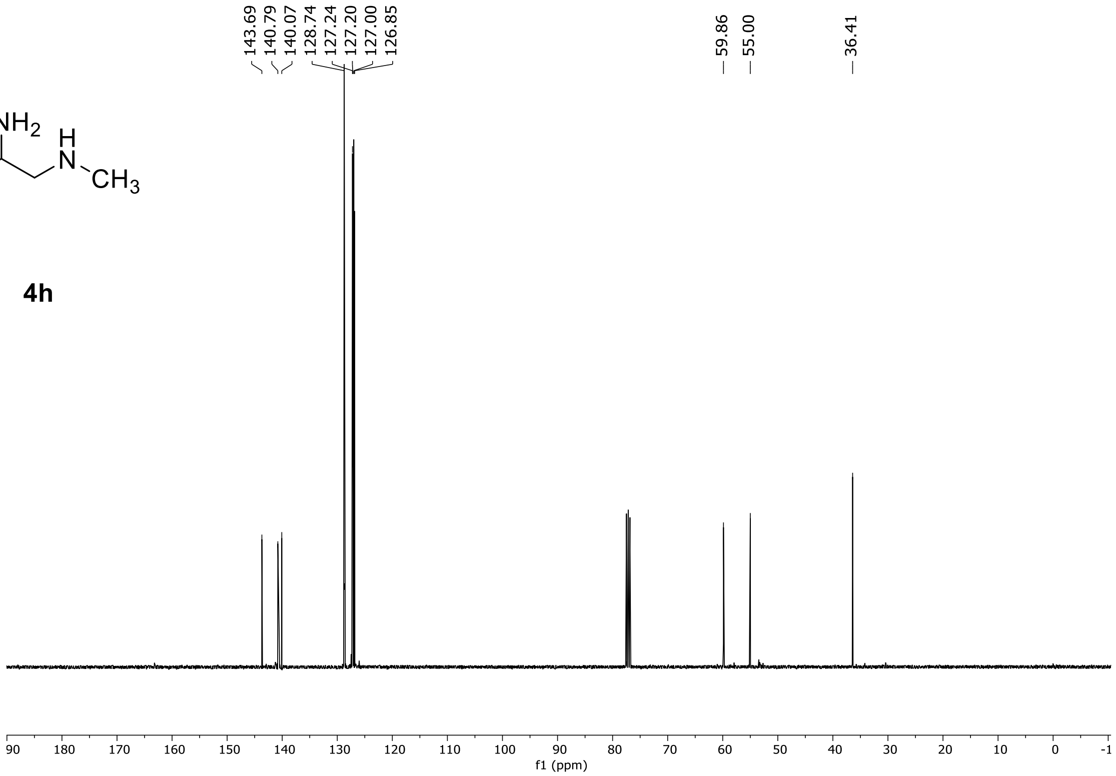

^{1}H NMR spectrum of **4i** (In $CDCl_3$, 400 MHz)

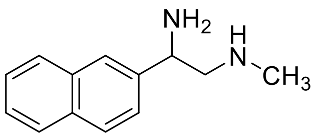

4i

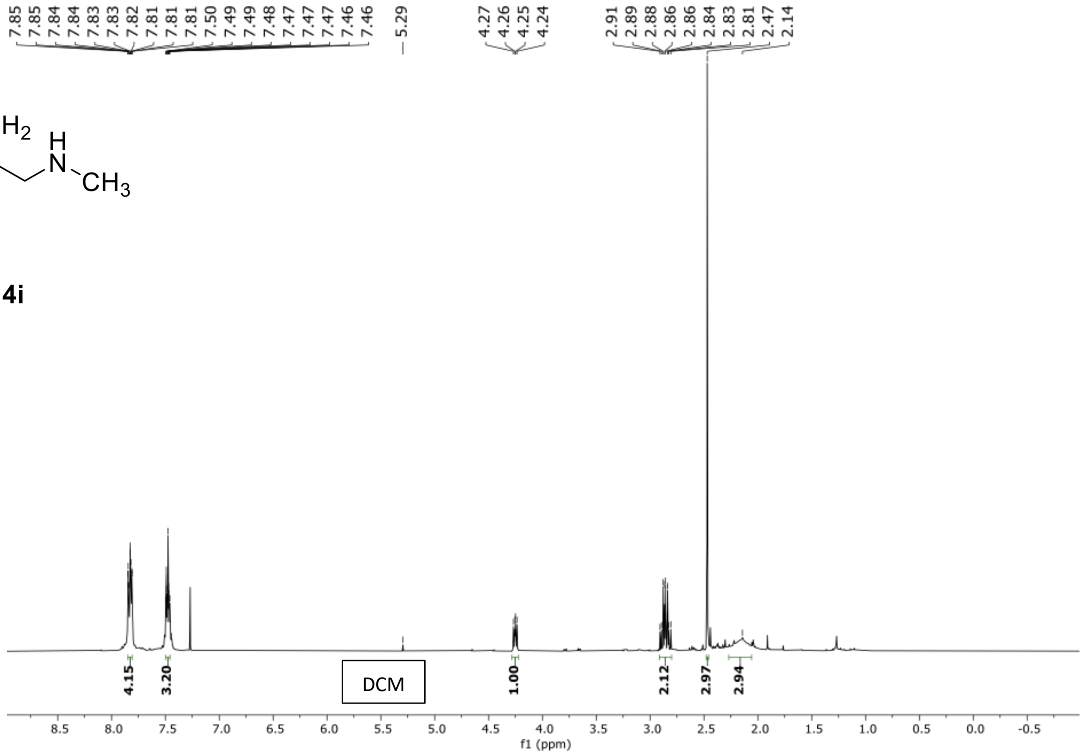

^{13}C NMR spectrum of **4i** (In $CDCl_3$, 101 MHz)

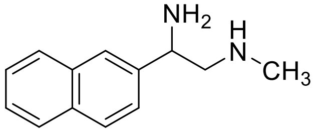

4i

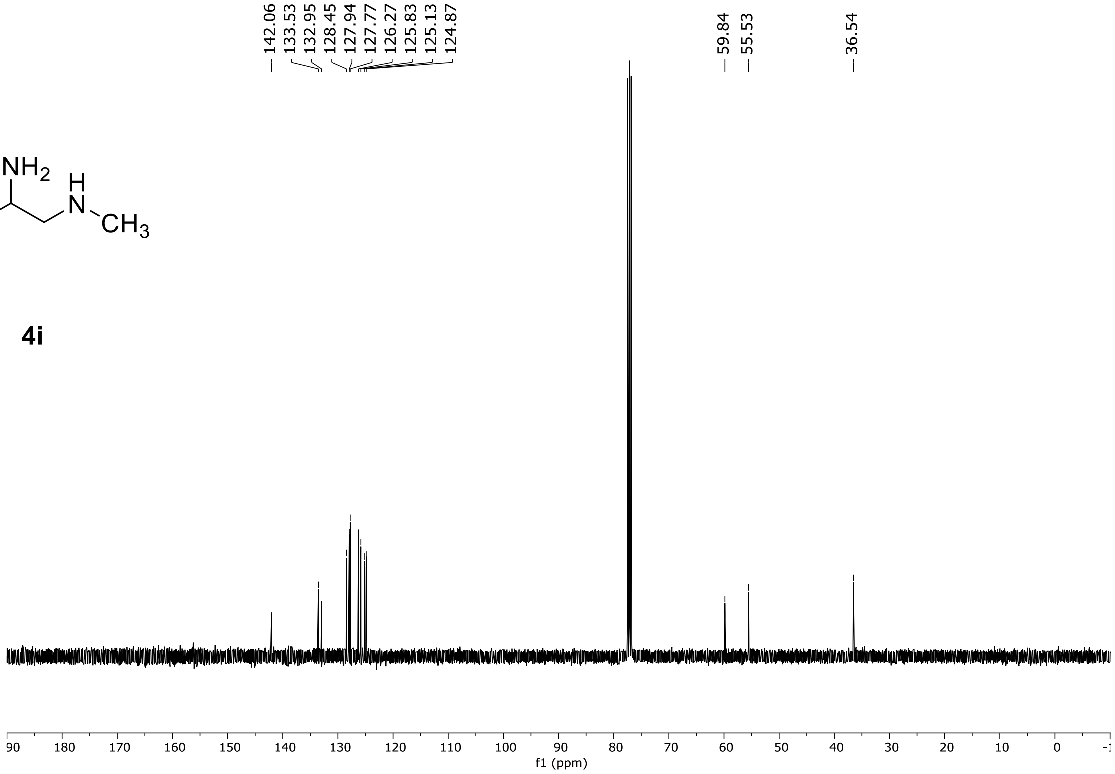

^{1}H NMR spectrum of **4j** (In MeOD, 400 MHz)

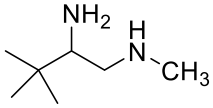

4j

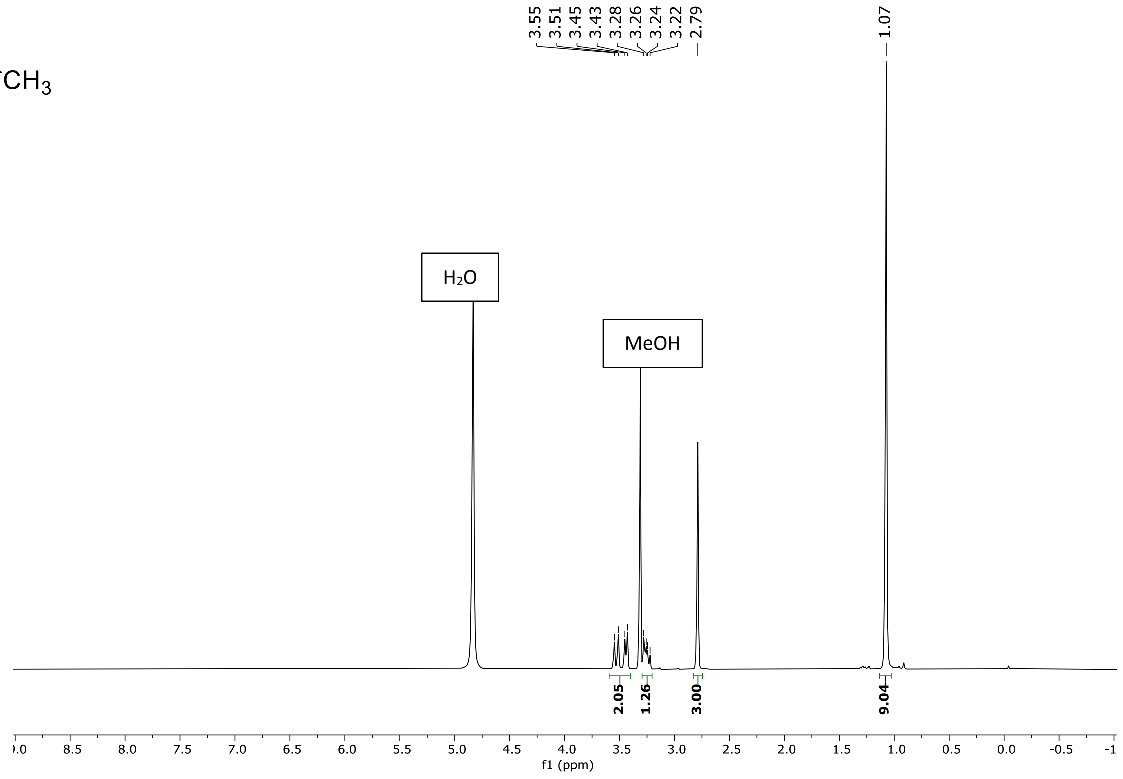

^{13}C NMR spectrum of **4j** (In MeOD, 101 MHz)

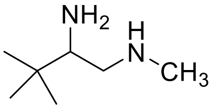

4j

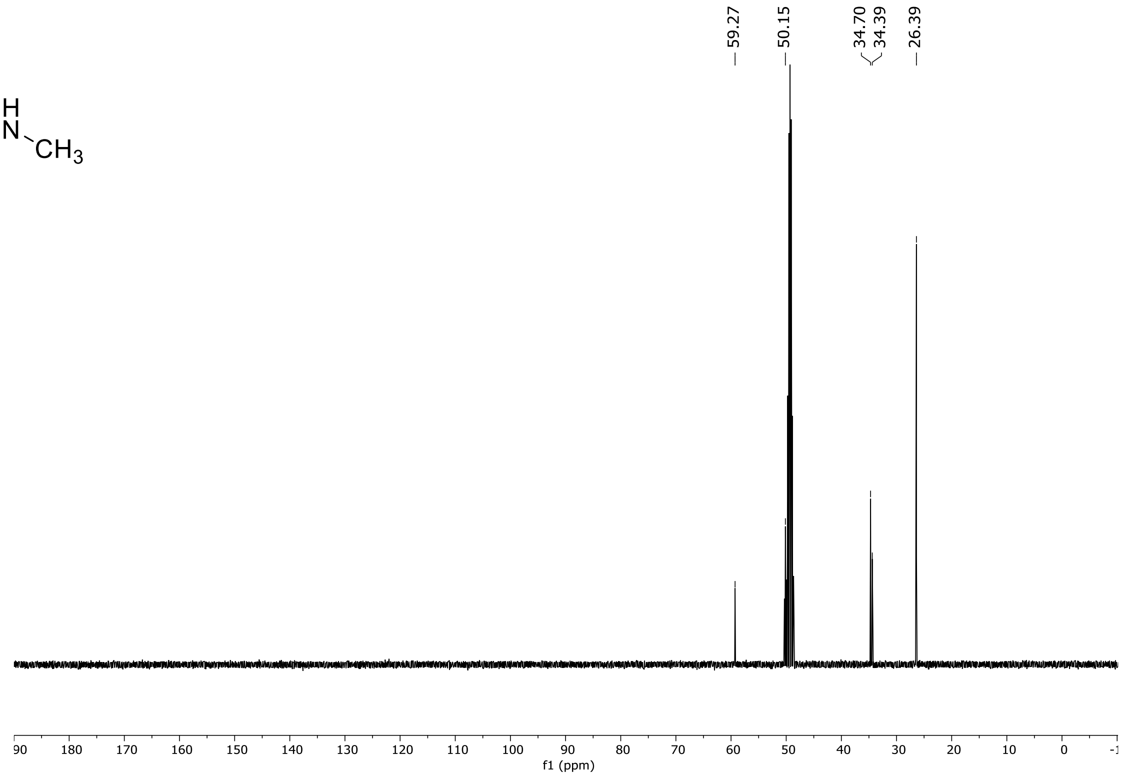

^{1}H NMR spectrum of **4k** (In $CDCl_3$, 400 MHz)

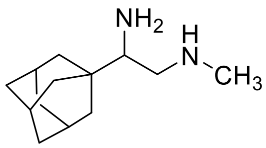

4k

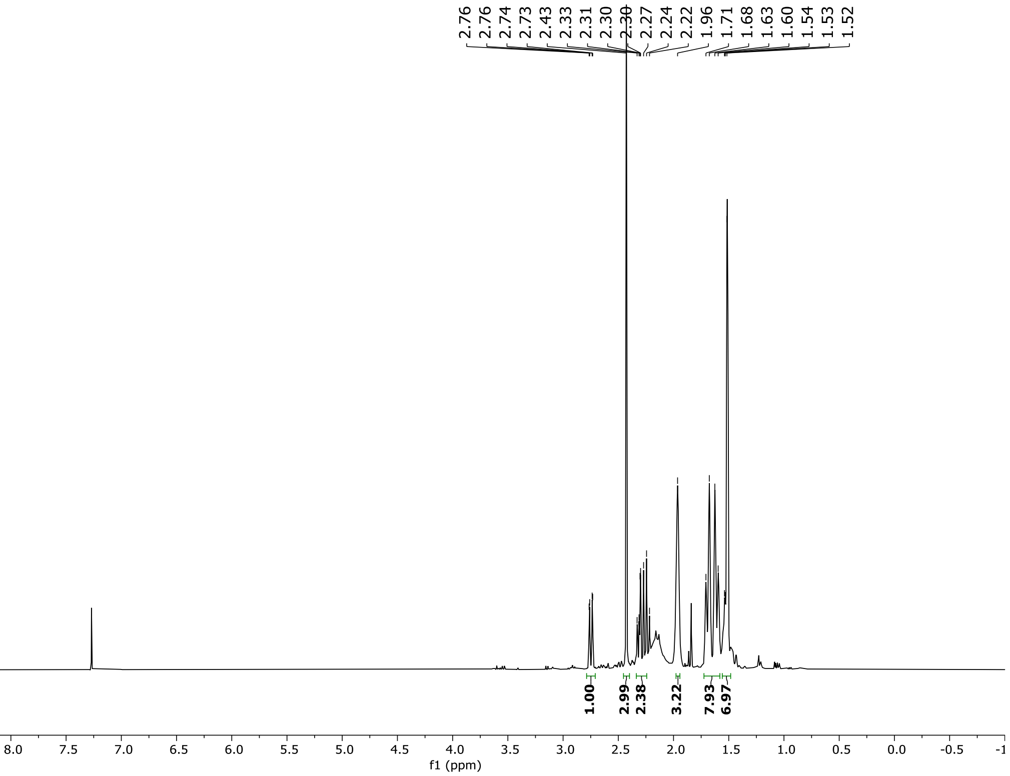

^{13}C NMR spectrum of **4k** (In $CDCl_3$, 101 MHz)

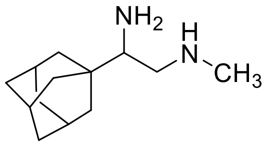

4k

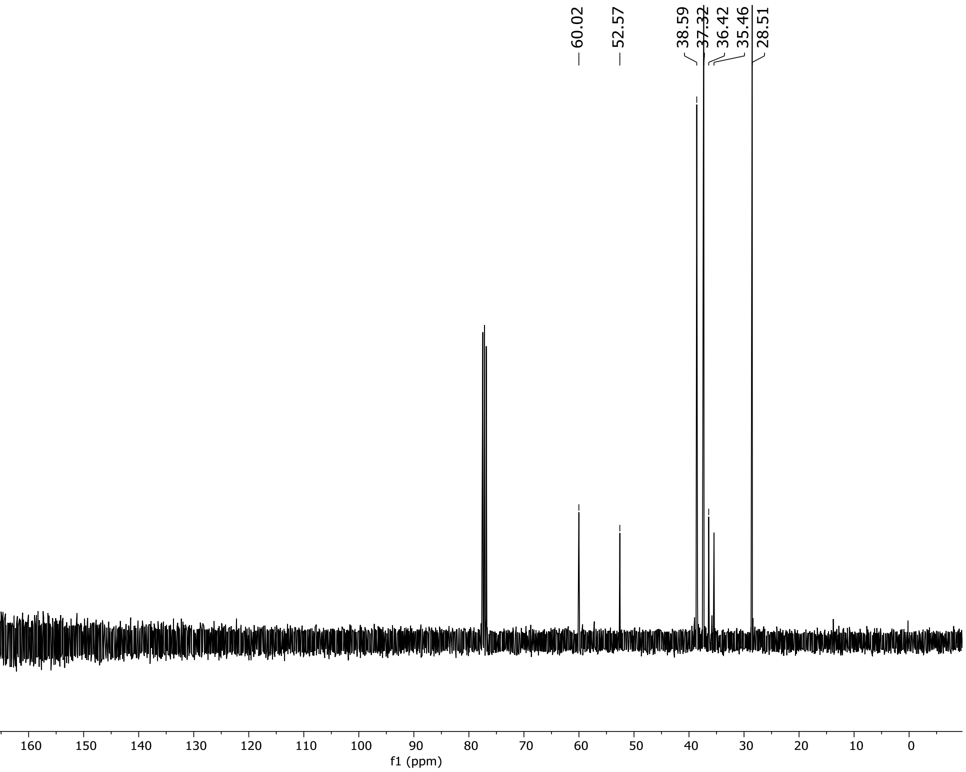

^{1}H NMR spectrum of **4l** (In $CDCl_3$, 400 MHz)

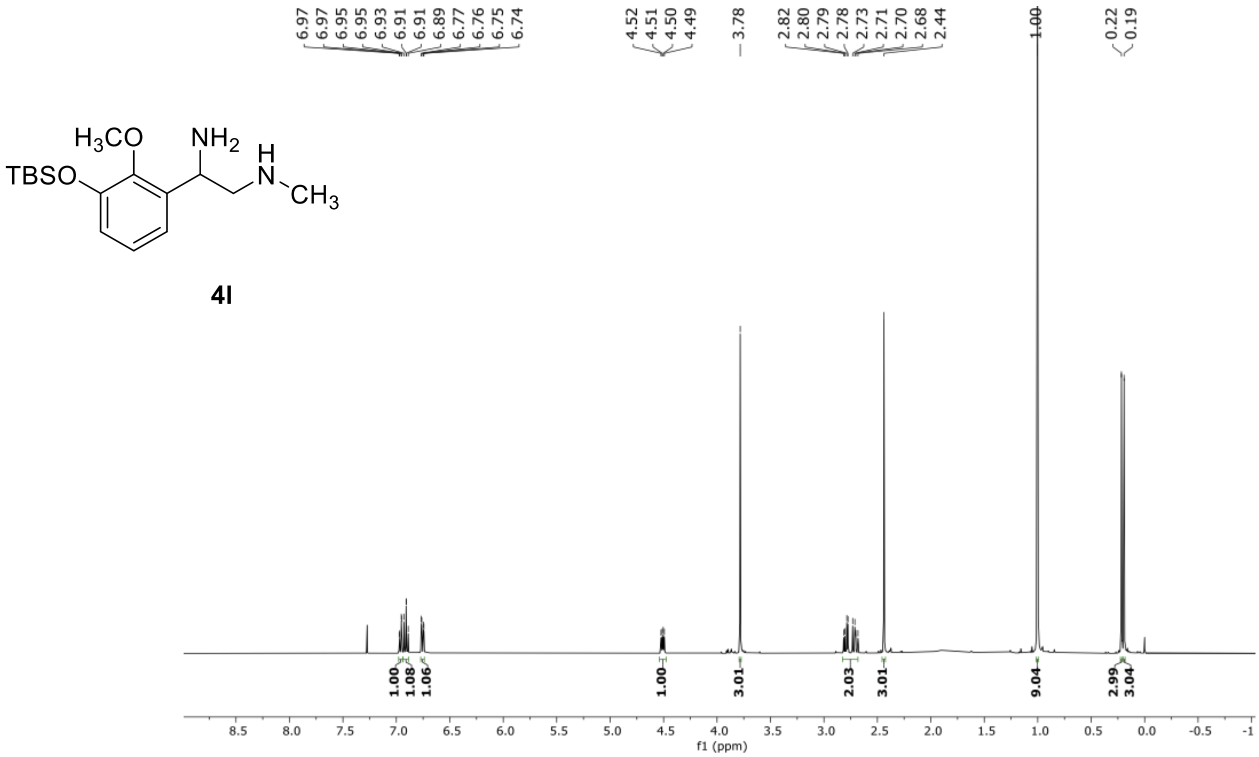

^{13}C NMR spectrum of **4l** (In $CDCl_3$, 101 MHz)

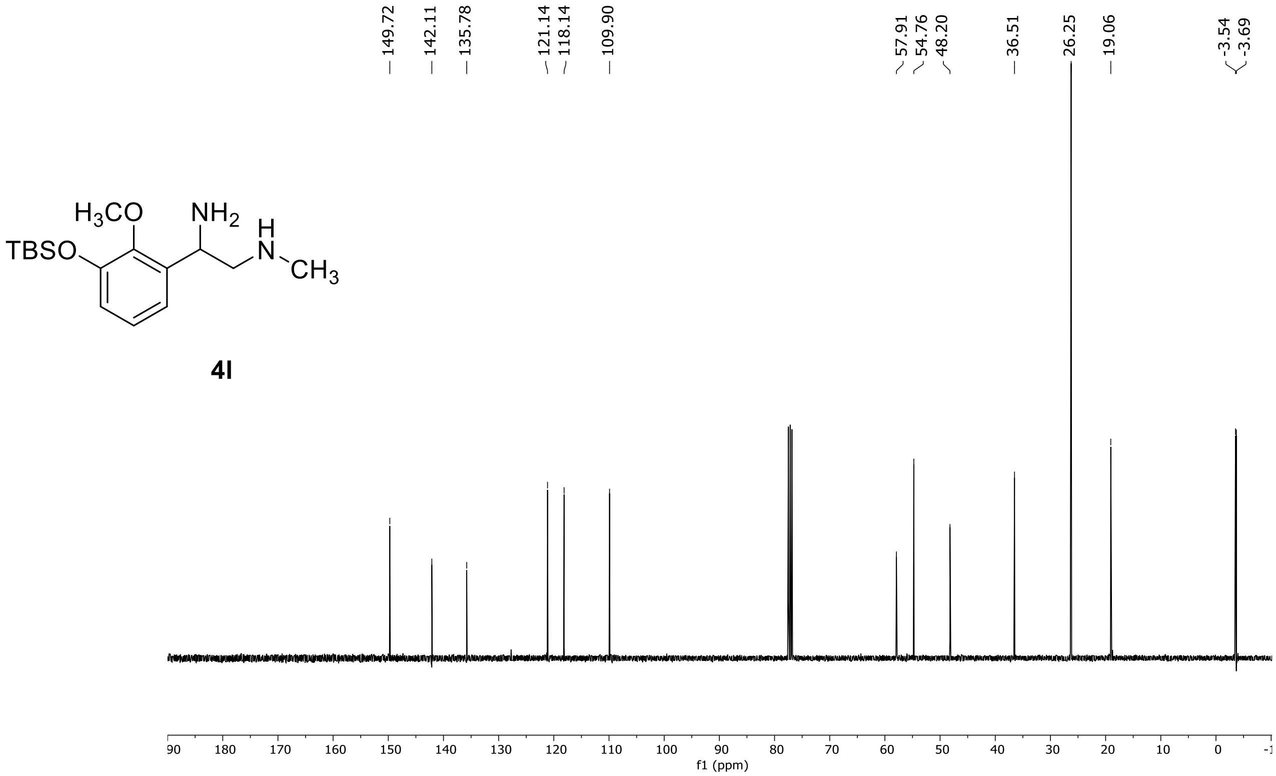

^{1}H NMR spectrum of **4m** (In $CDCl_3$, 400 MHz)

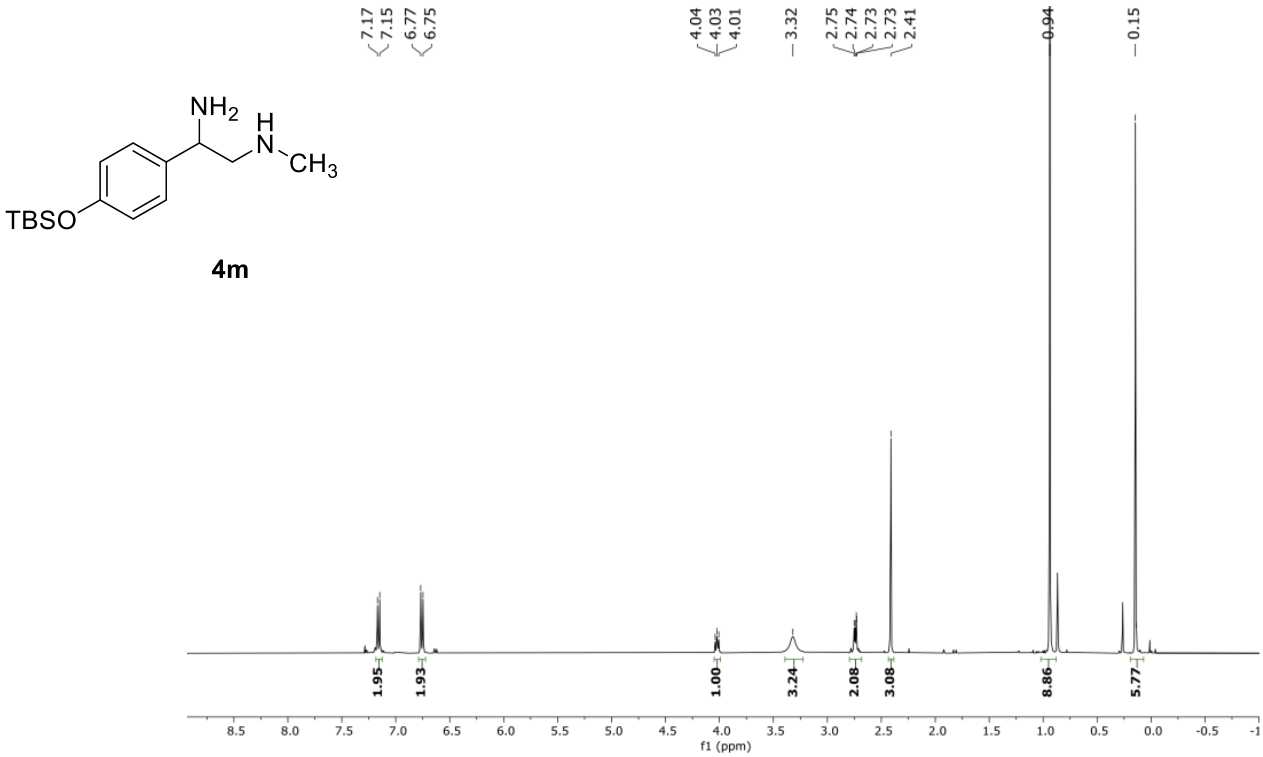

^{13}C NMR spectrum of **4m** (In $CDCl_3$, 126 MHz)

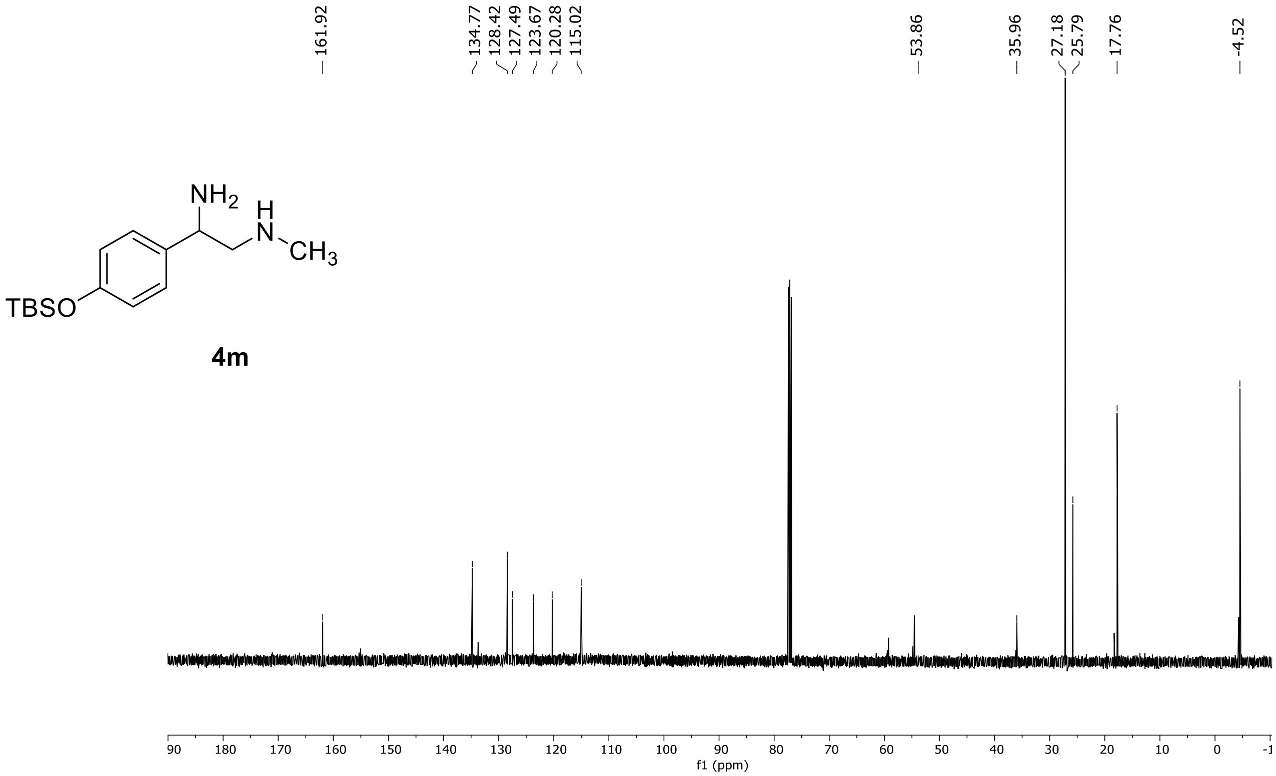

^{1}H NMR spectrum of **4n** (In $CDCl_3$, 400 MHz)

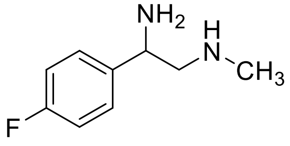

4n

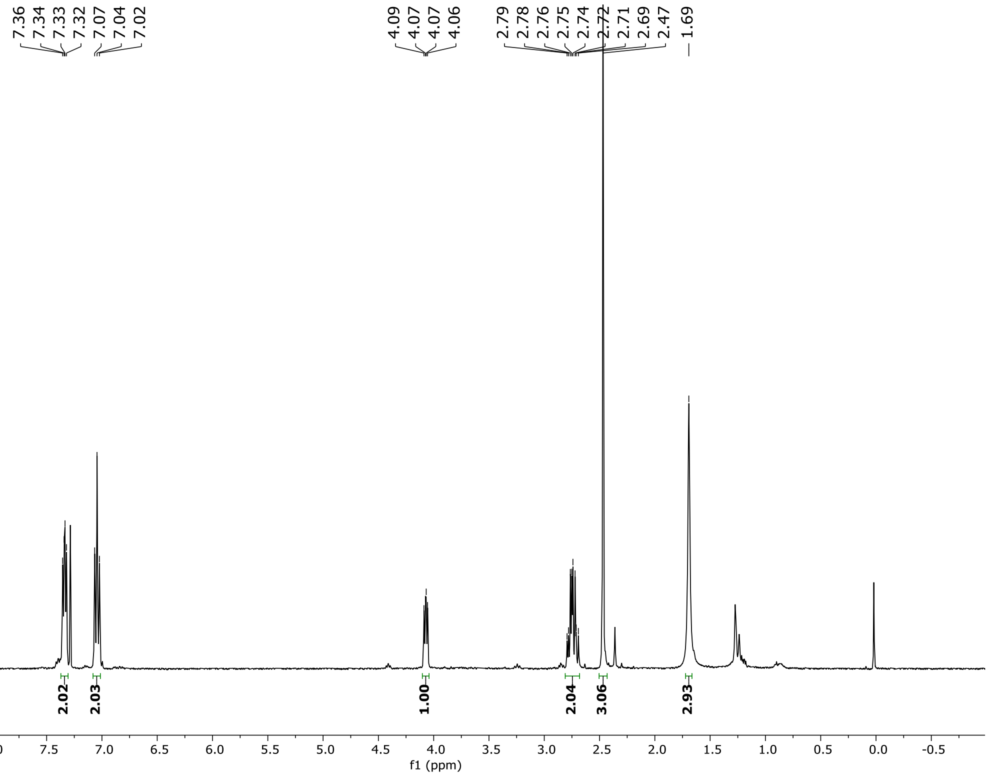

^{13}C NMR spectrum of **4n** (In $CDCl_3$, 101 MHz)

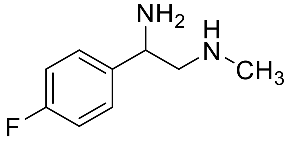

4n

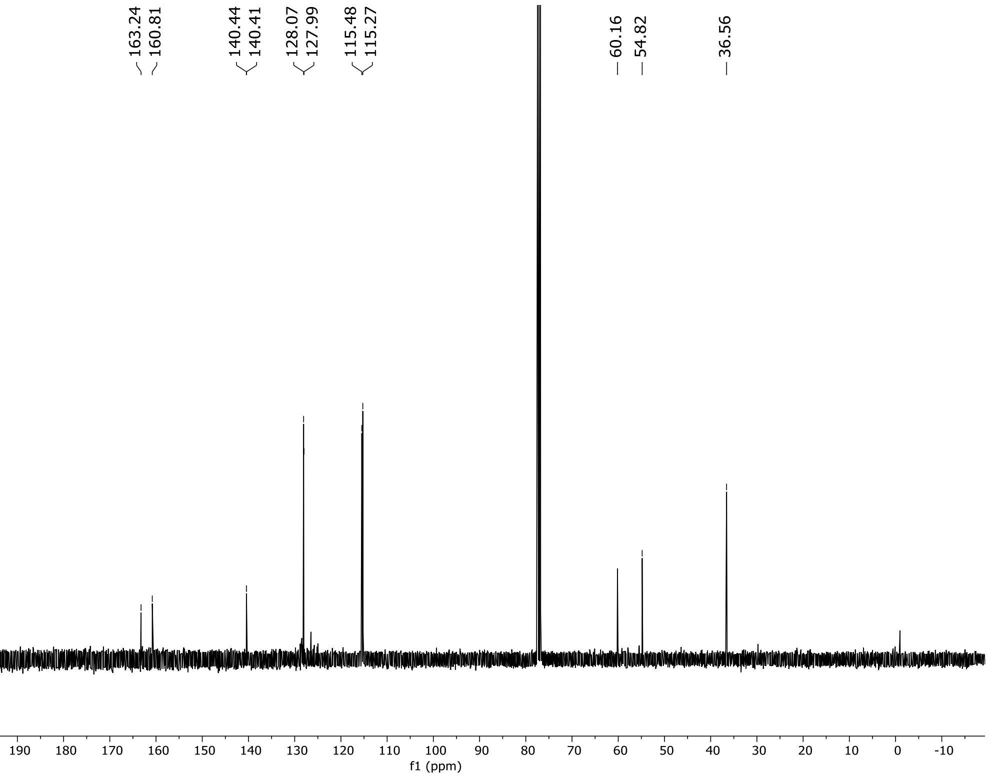

^{1}H NMR spectrum of **4o** (In $CDCl_3$, 400 MHz)

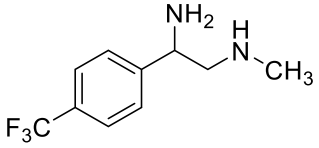

4o

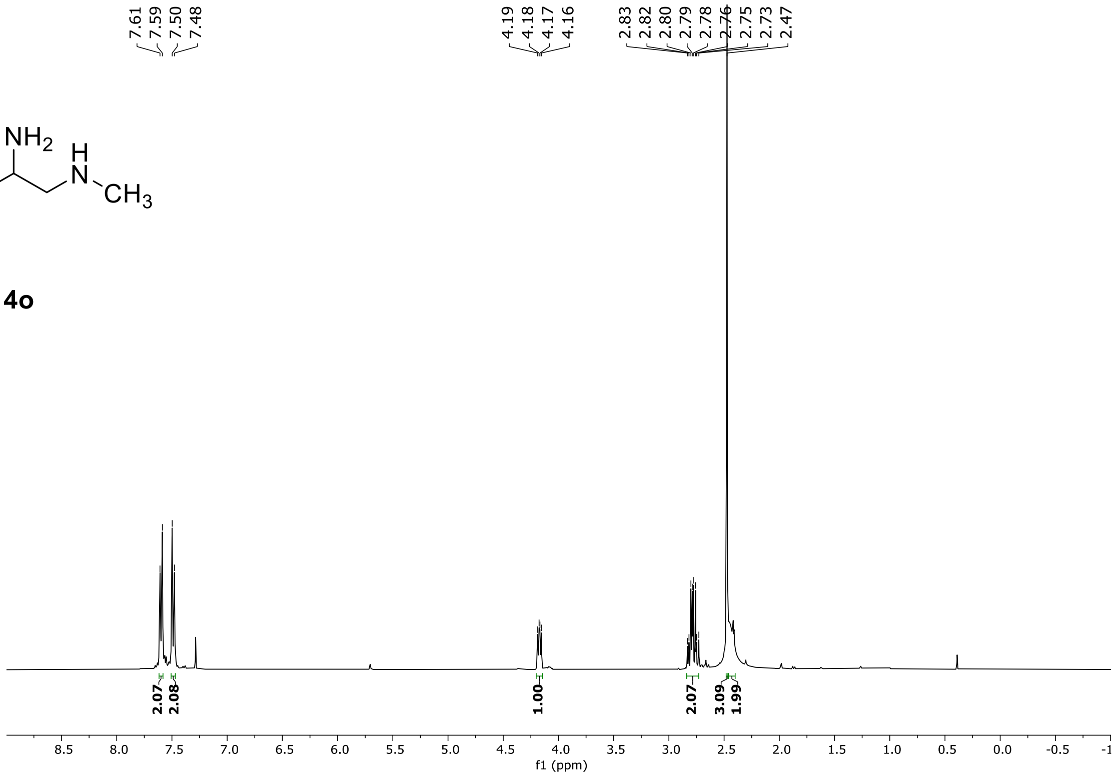

^{13}C NMR spectrum of **4o** (In $CDCl_3$, 101 MHz)

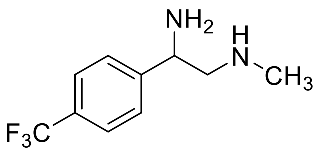

4o

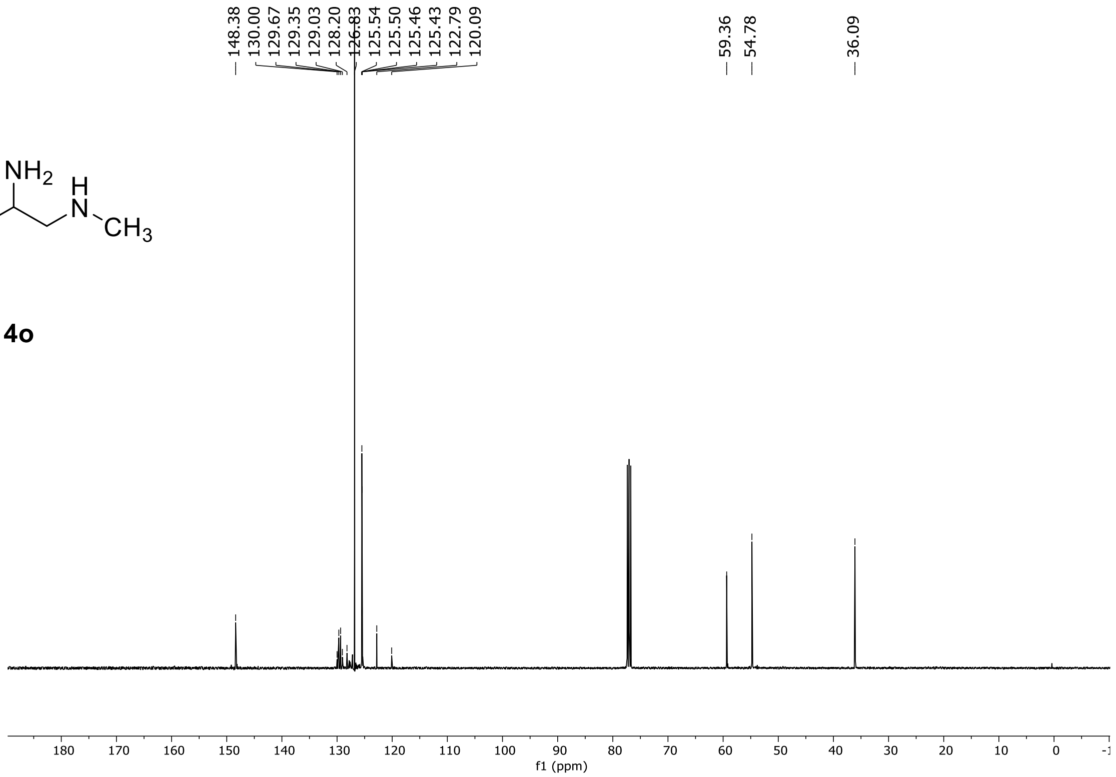

^{1}H NMR spectrum of **4p** (In $CDCl_3$, 400 MHz)

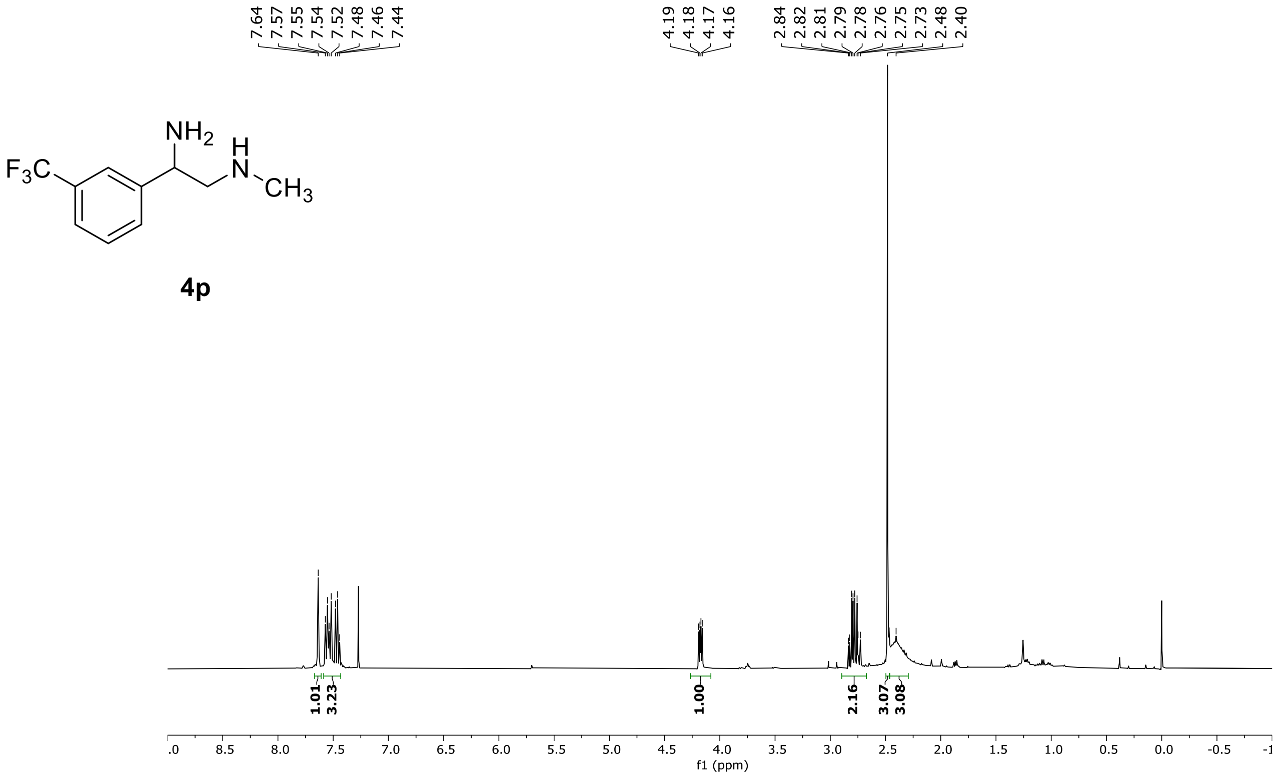

^{13}C NMR spectrum of **4p** (In $CDCl_3$, 101 MHz)

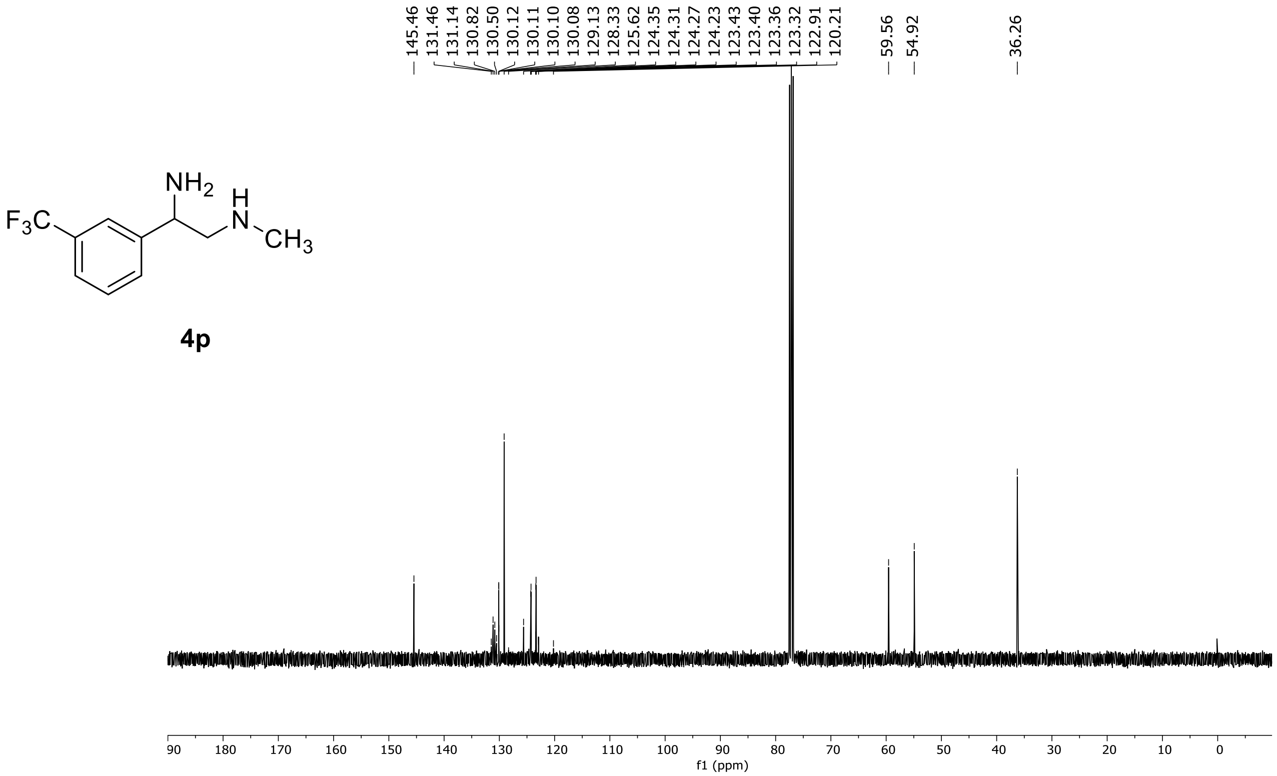

^{1}H NMR spectrum of **4q** (In $CDCl_3$, 400 MHz)

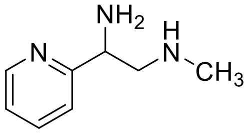

4q

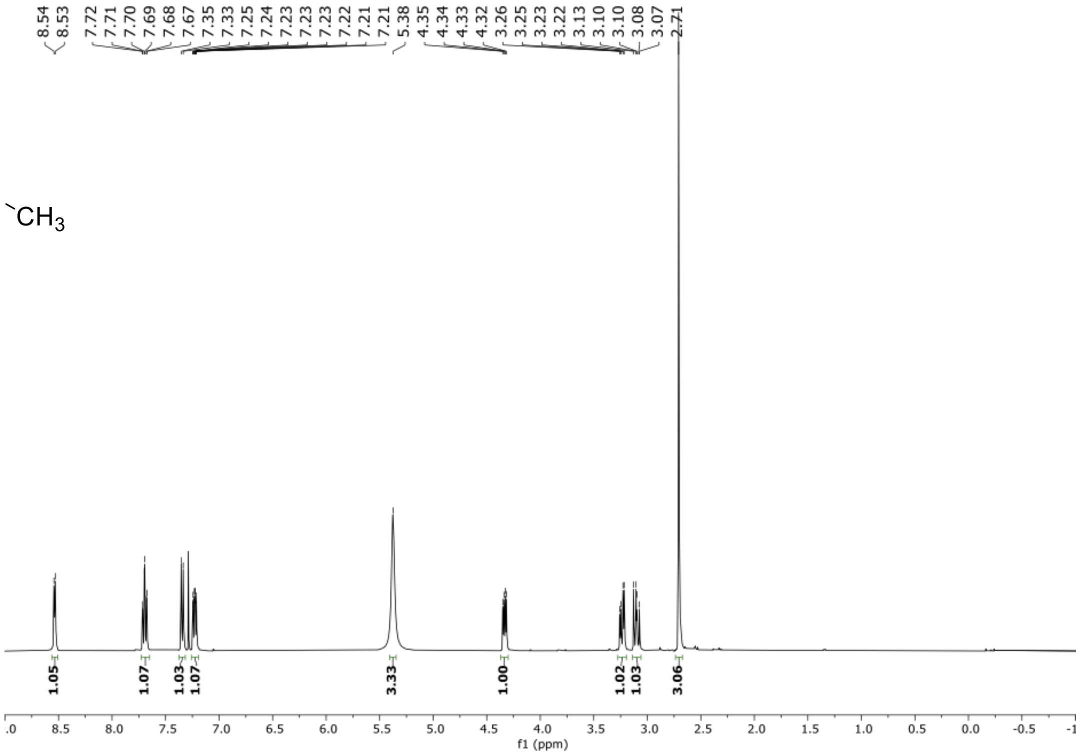

^{13}C NMR spectrum of **4q** (In $CDCl_3$, 101 MHz)

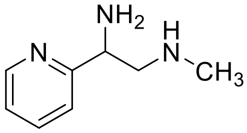

4q

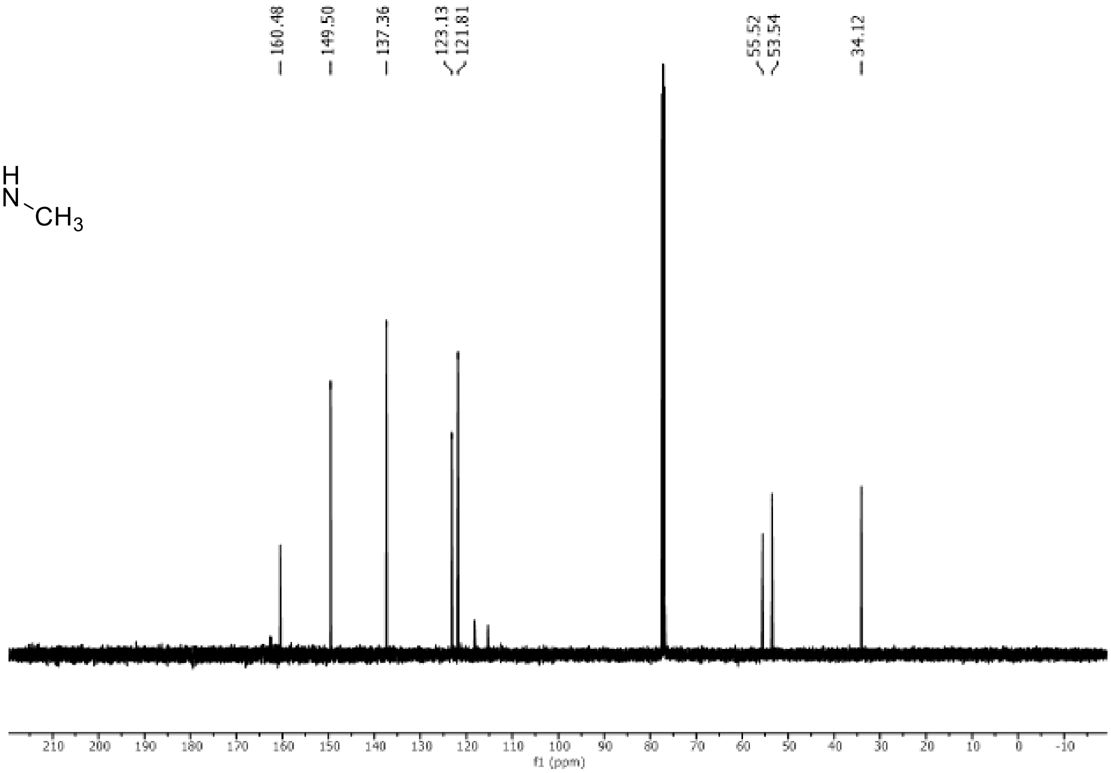

^{1}H NMR spectrum of **4r** (In $CDCl_3$, 400 MHz)

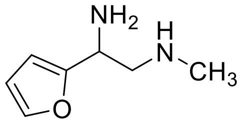

4r

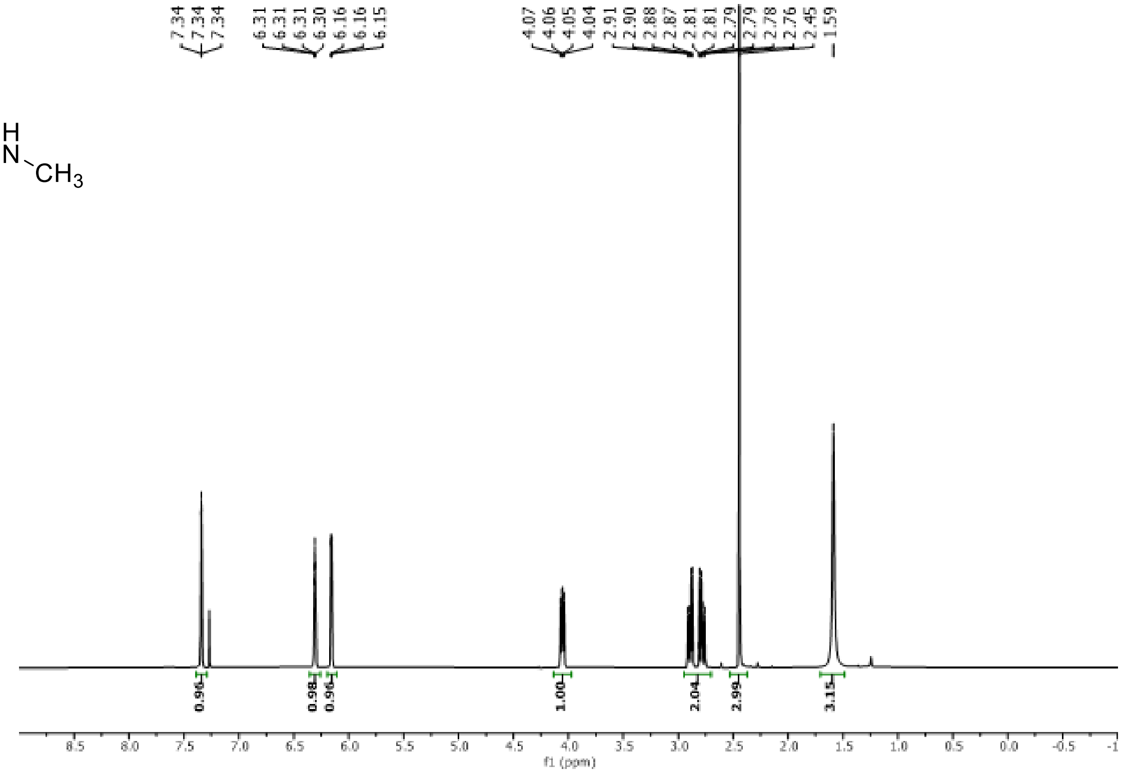

2D COSY spectrum of **4r** (In $CDCl_3$, 400 MHz)

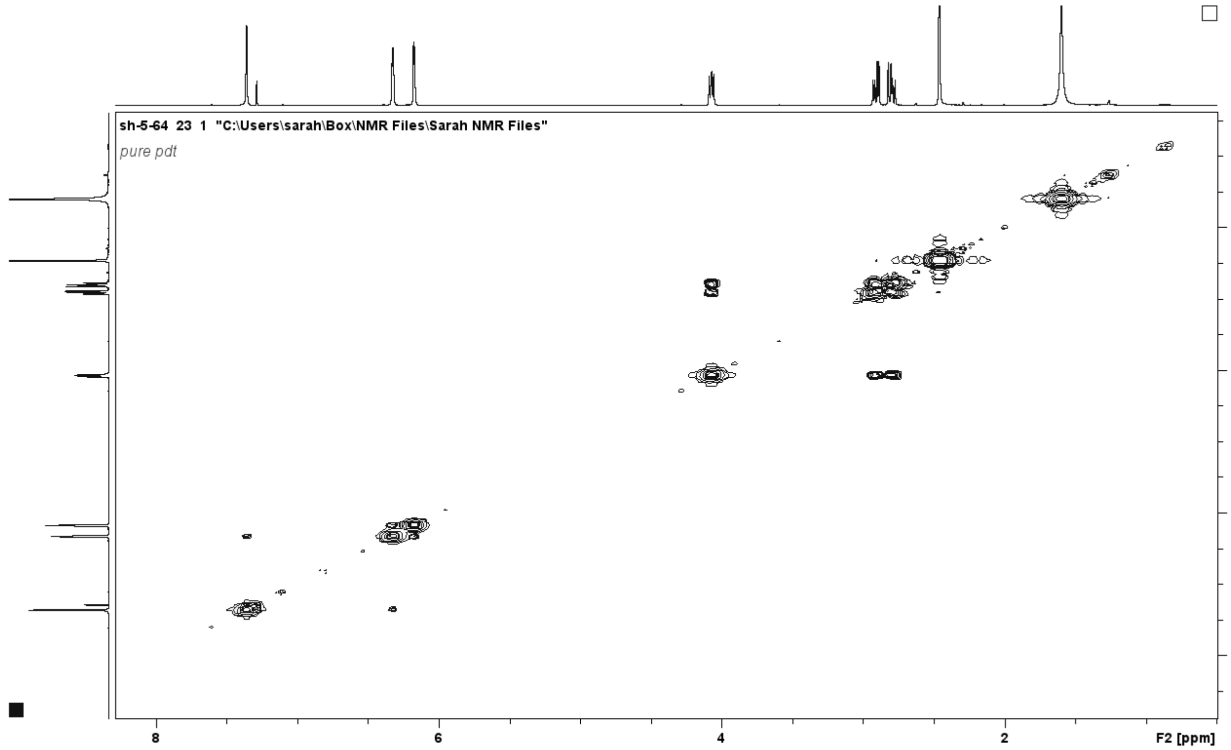

^{13}C NMR spectrum of **4r** (In $CDCl_3$, 101 MHz)

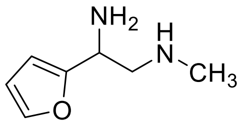

4r

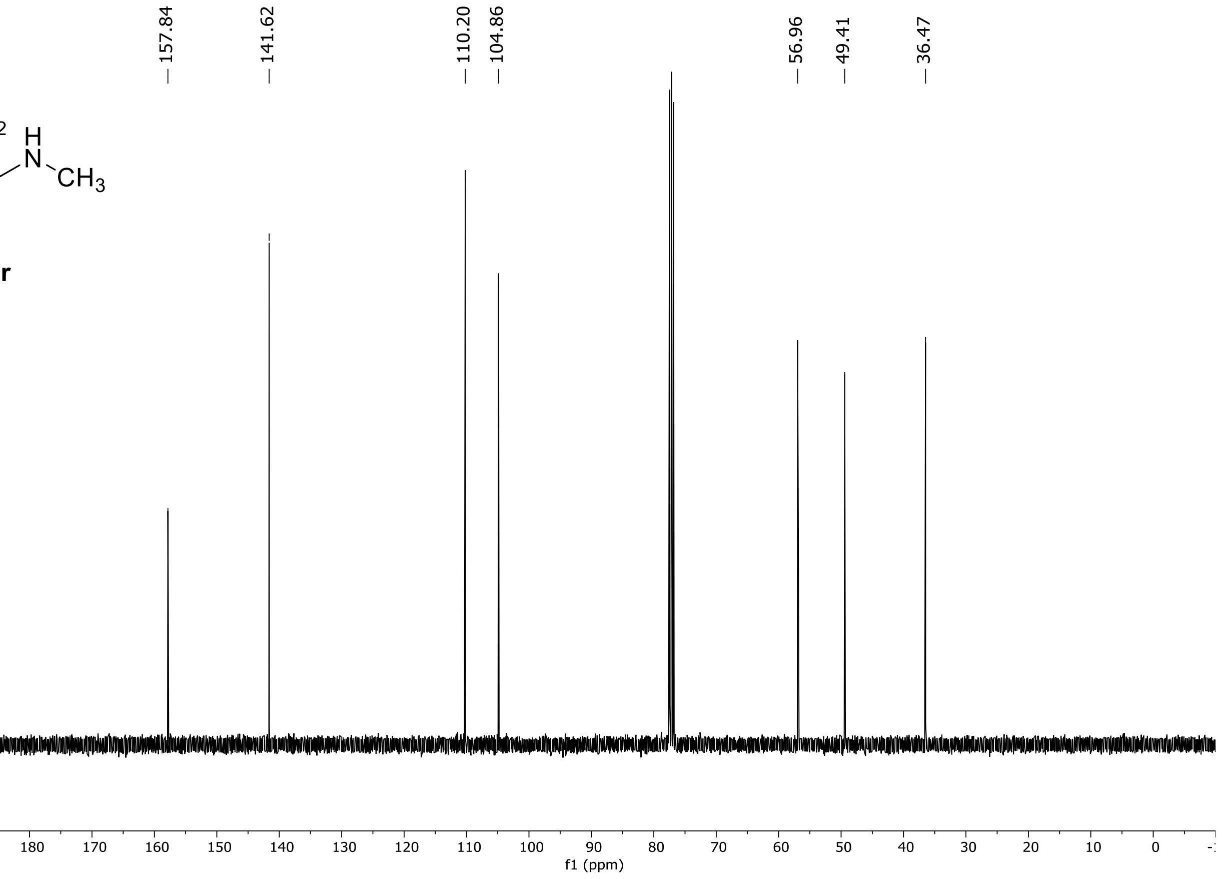

^{1}H NMR spectrum of **4s** (In $CDCl_3$, 400 MHz)

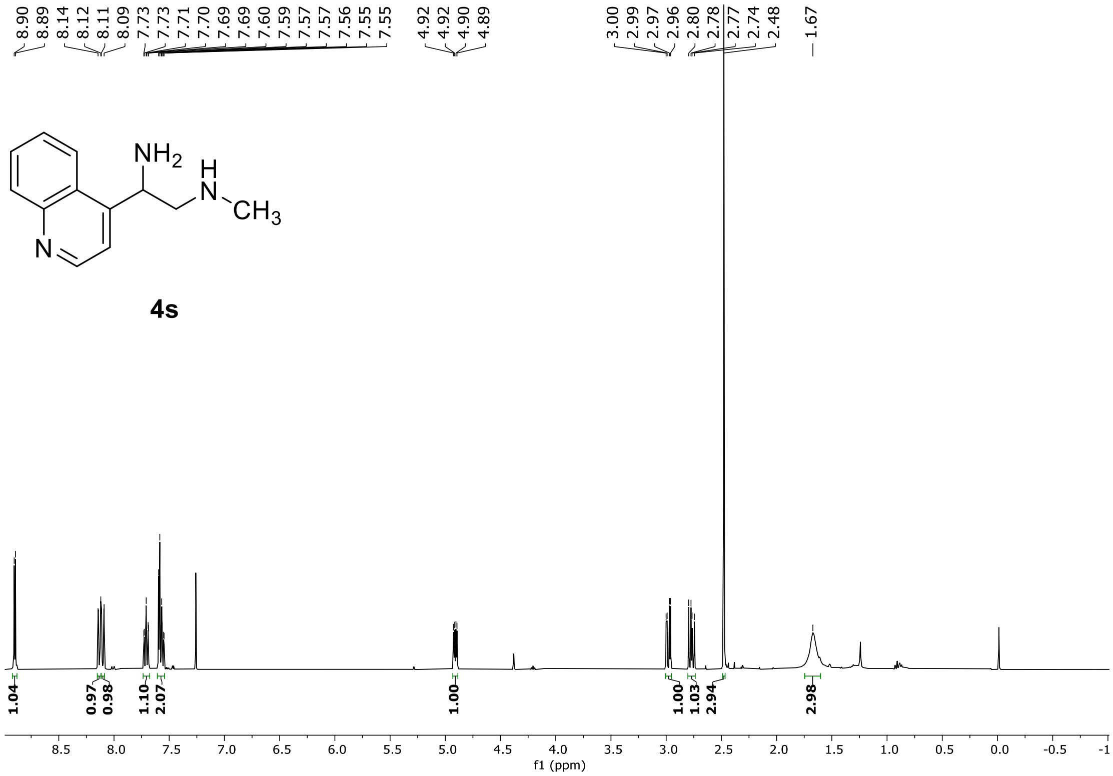

^{13}C NMR spectrum of **4s** (In $CDCl_3$, 101 MHz)

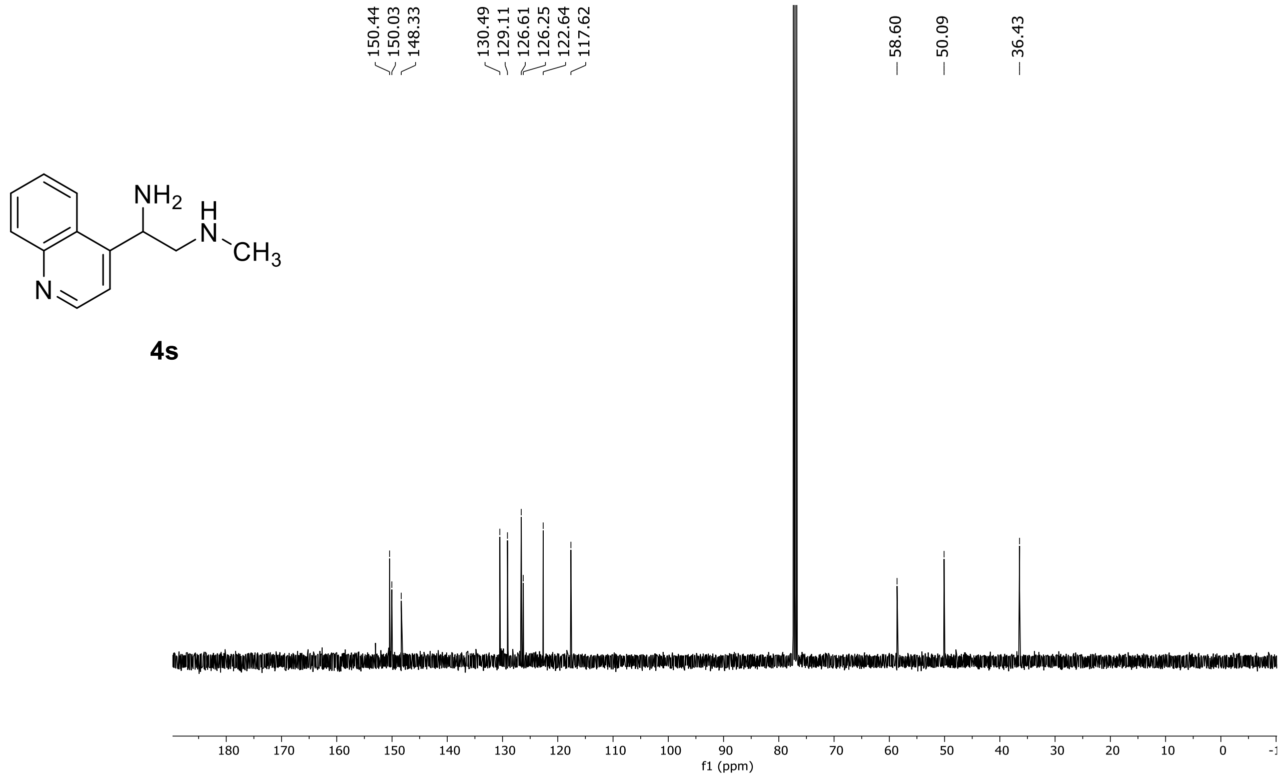

^{1}H NMR spectrum of **4t** (In $CDCl_3$, 400 MHz)

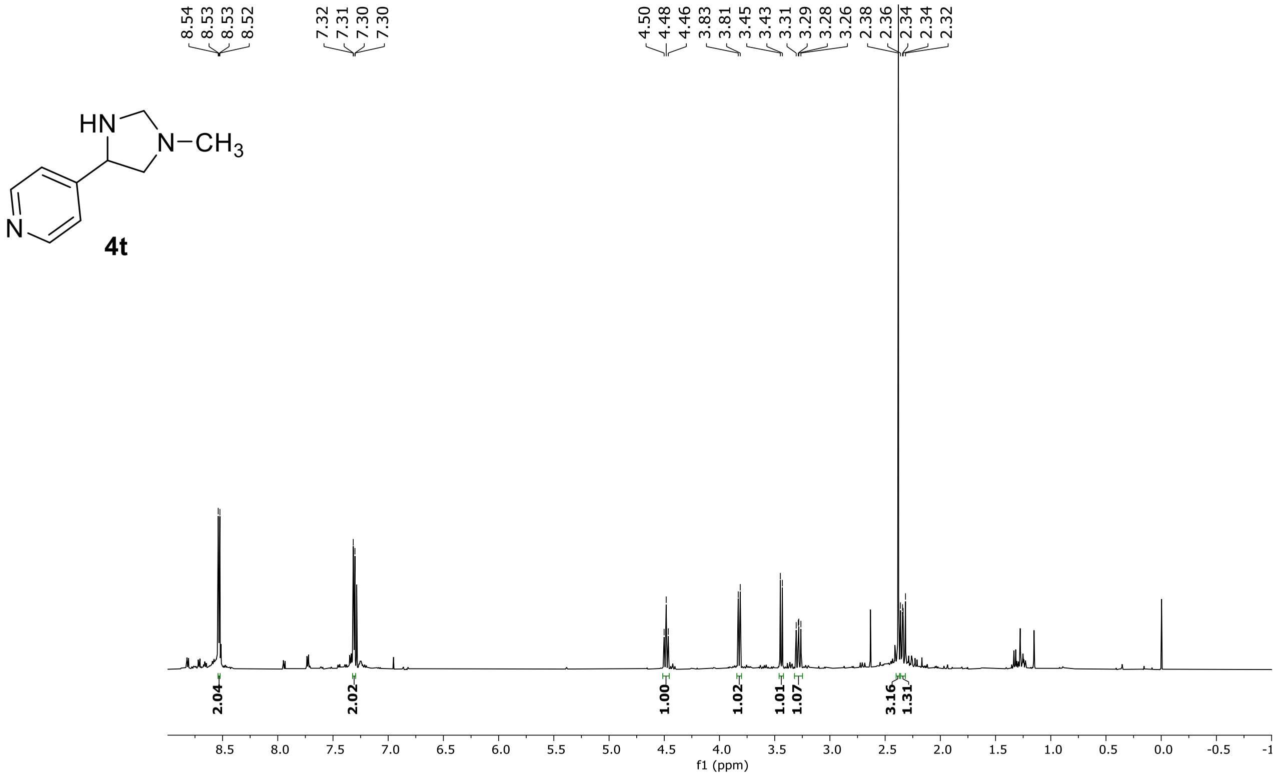

^{13}C NMR spectrum of **4t** (In $CDCl_3$, 101 MHz)

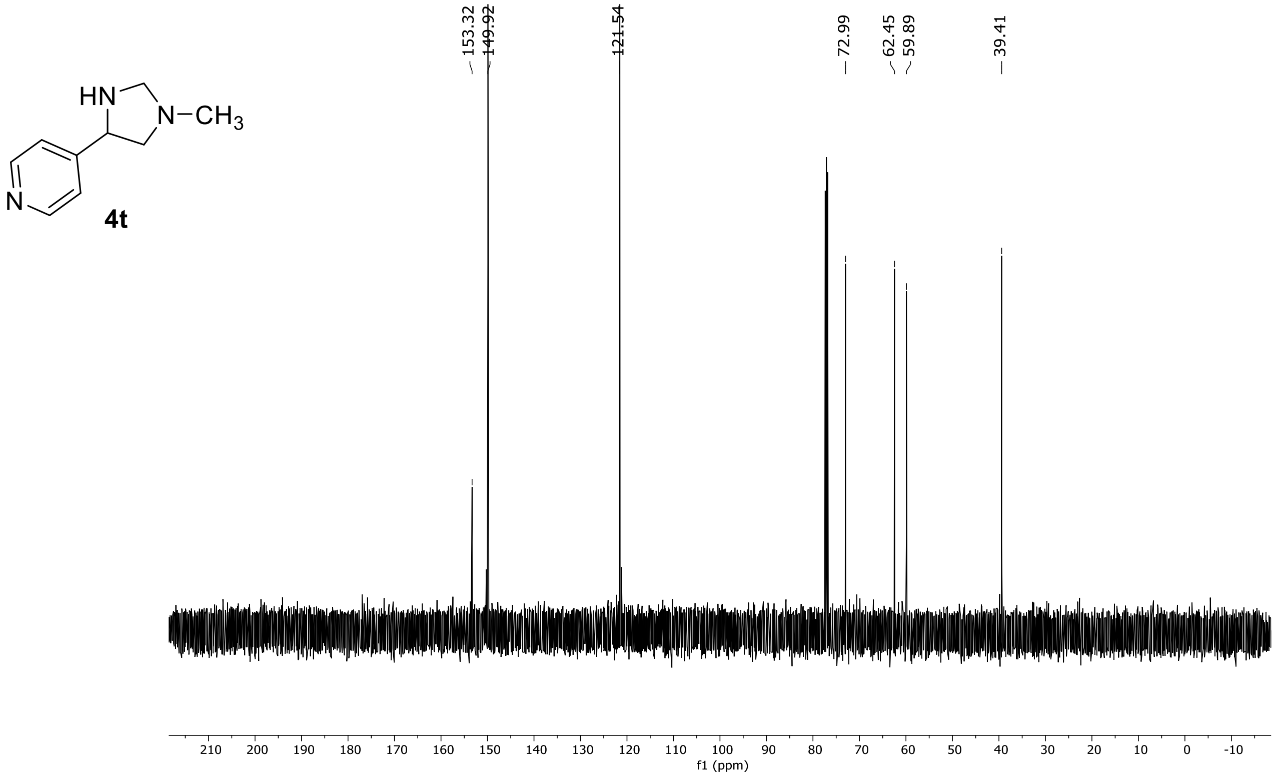

^{1}H NMR spectrum of **4u** (In $CDCl_3$, 400 MHz)

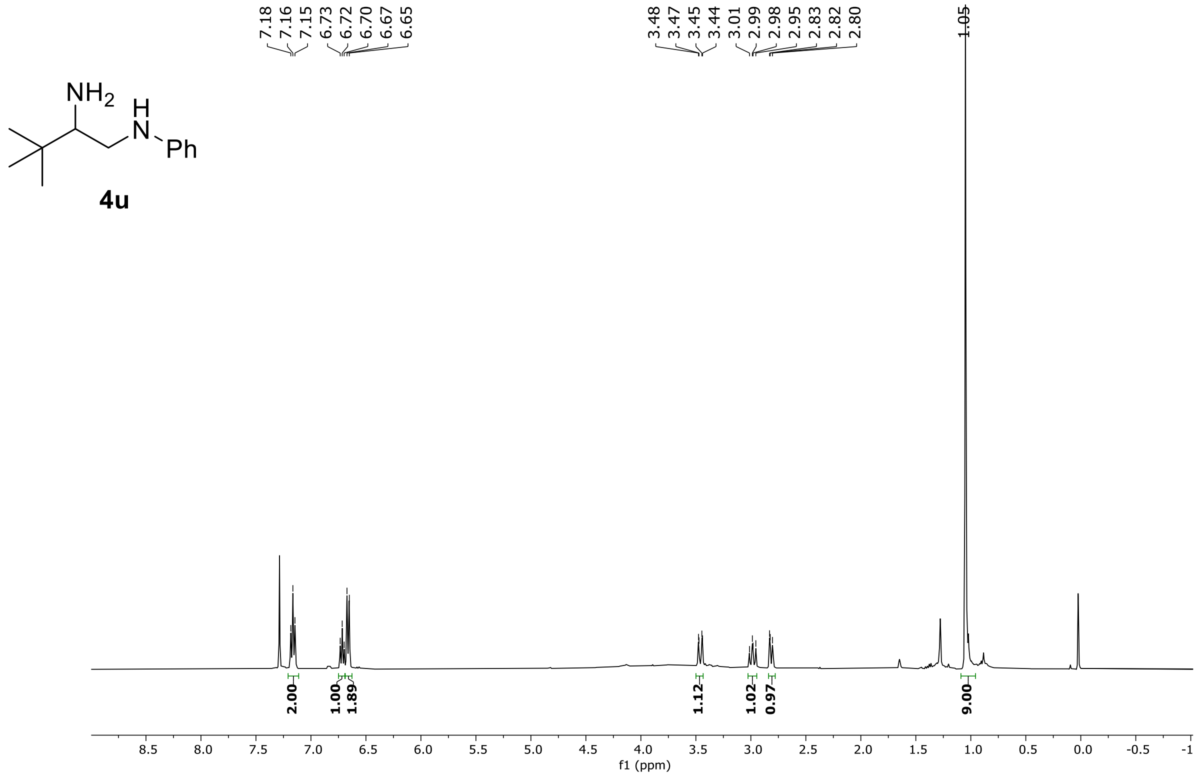

^{13}C NMR spectrum of **4u** (In $CDCl_3$, 101 MHz)

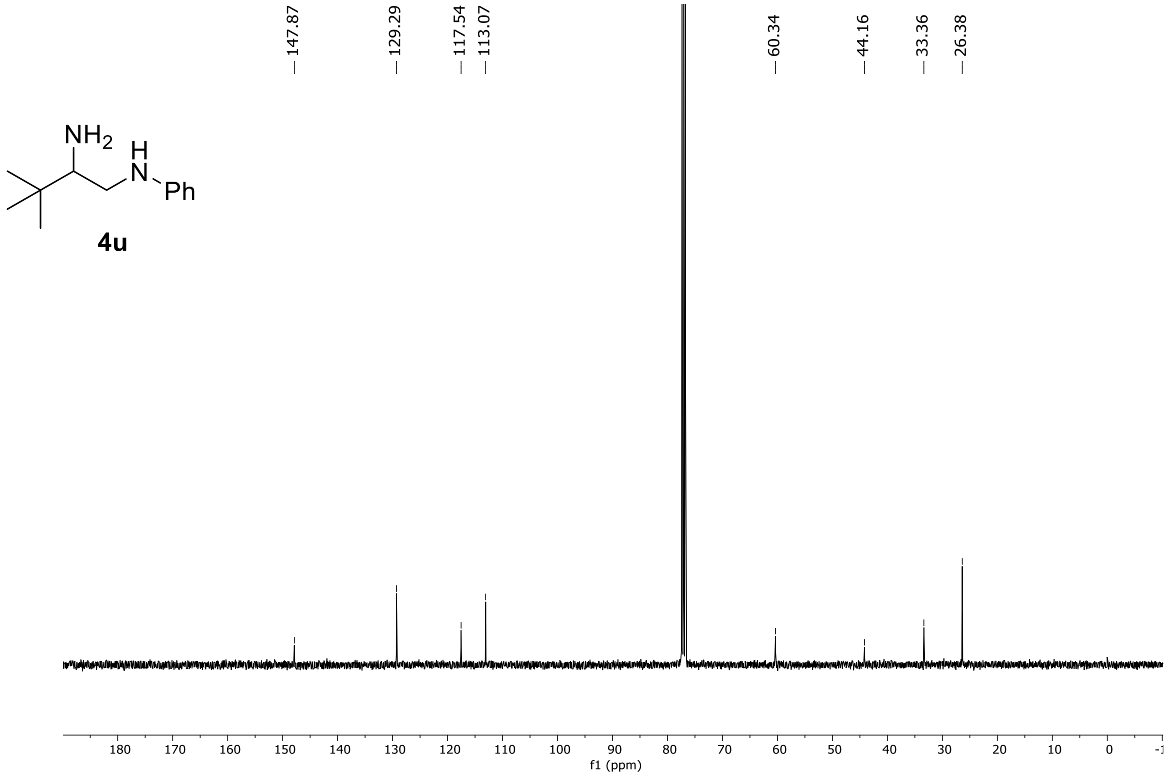

^{1}H NMR spectrum of **4v** (In $CDCl_3$, 400 MHz)

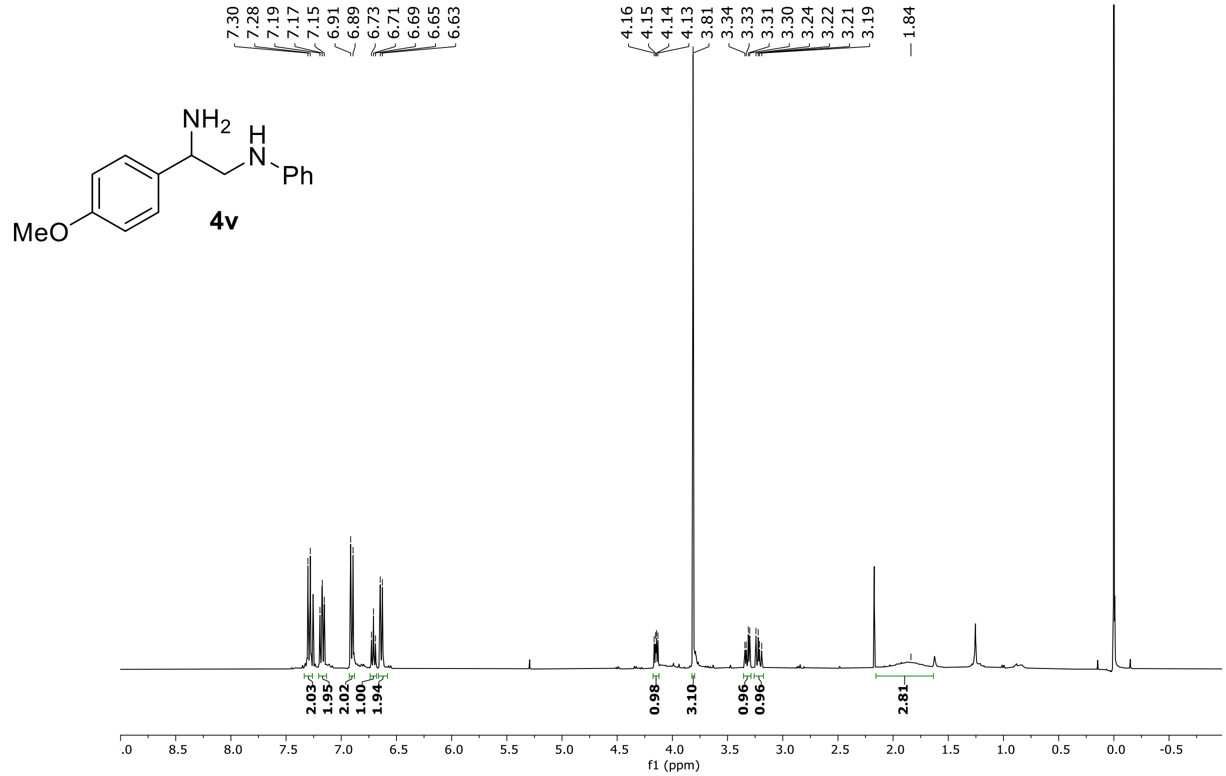

^{13}C NMR spectrum of **4v** (In $CDCl_3$, 101 MHz)

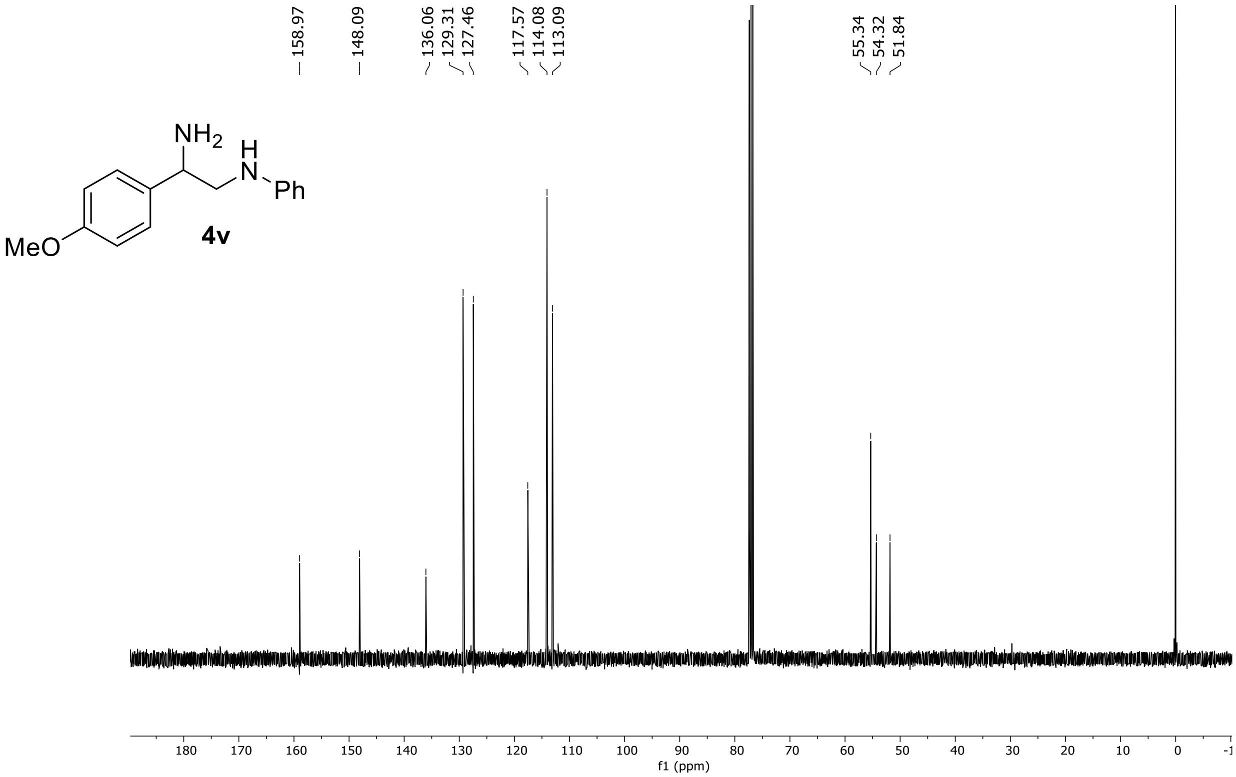

^{1}H NMR spectrum of **5** (In $CDCl_3$, 400 MHz)

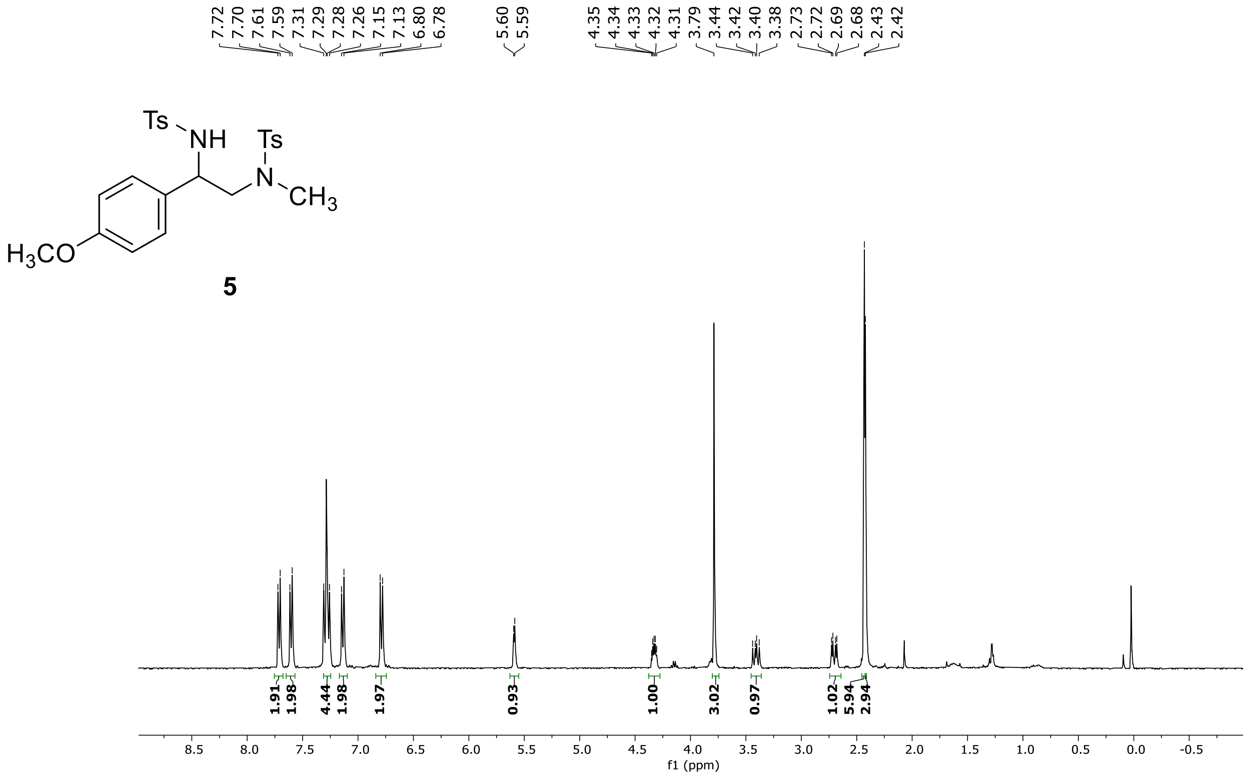

^{13}C NMR spectrum of **5** (In $CDCl_3$, 126 MHz)

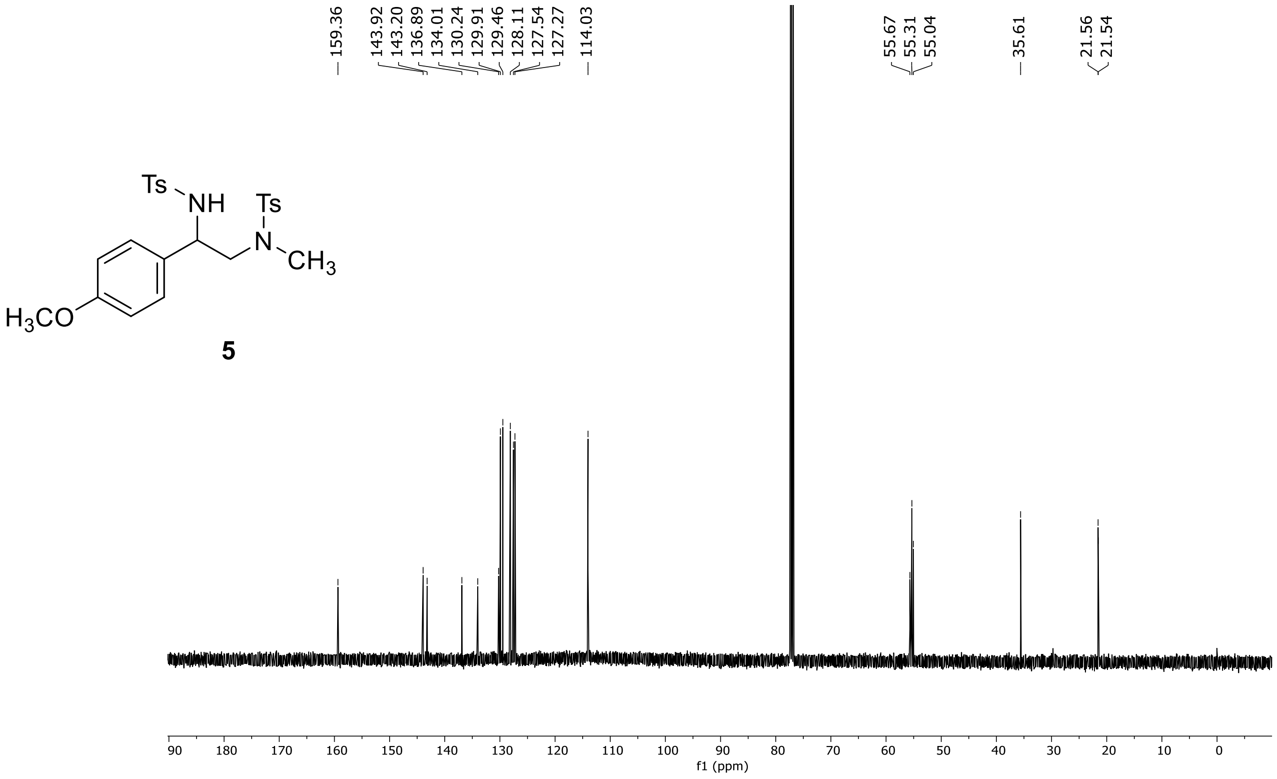

^{1}H NMR spectrum of **6** (In $CDCl_3$, 400 MHz)

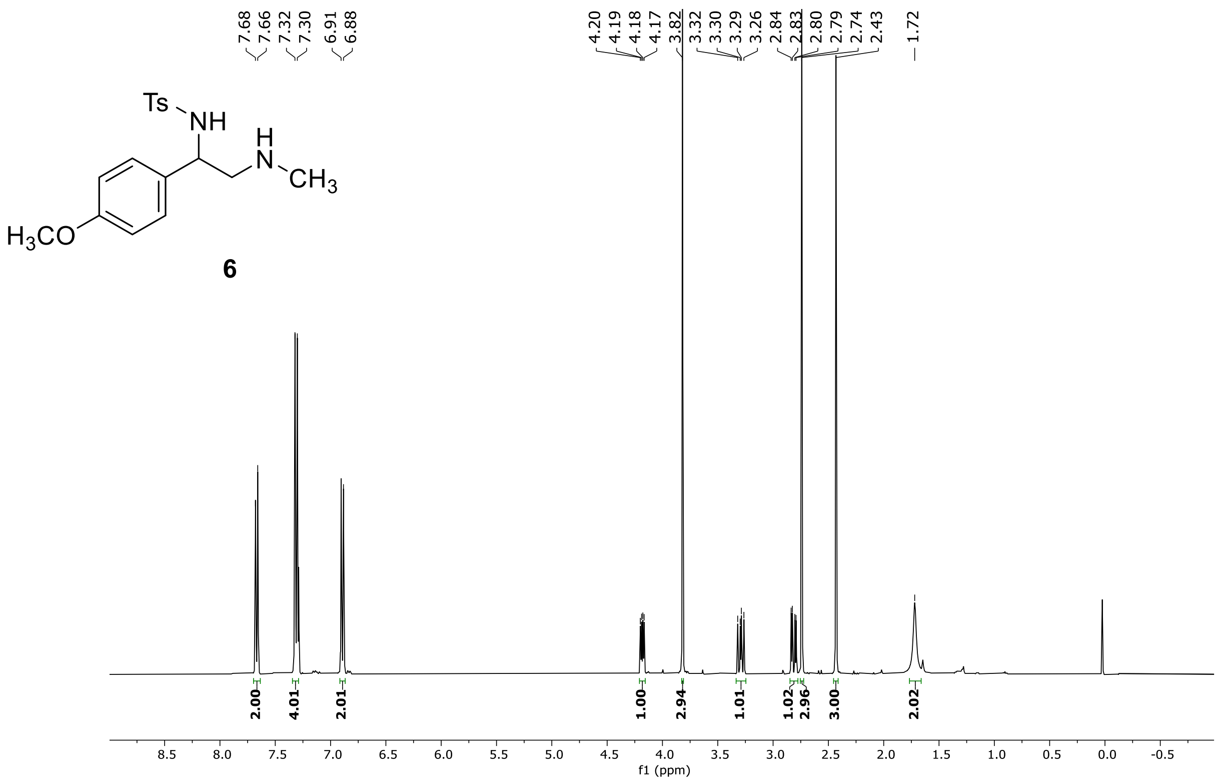

^{13}C NMR spectrum of **6** (In $CDCl_3$, 101 MHz)

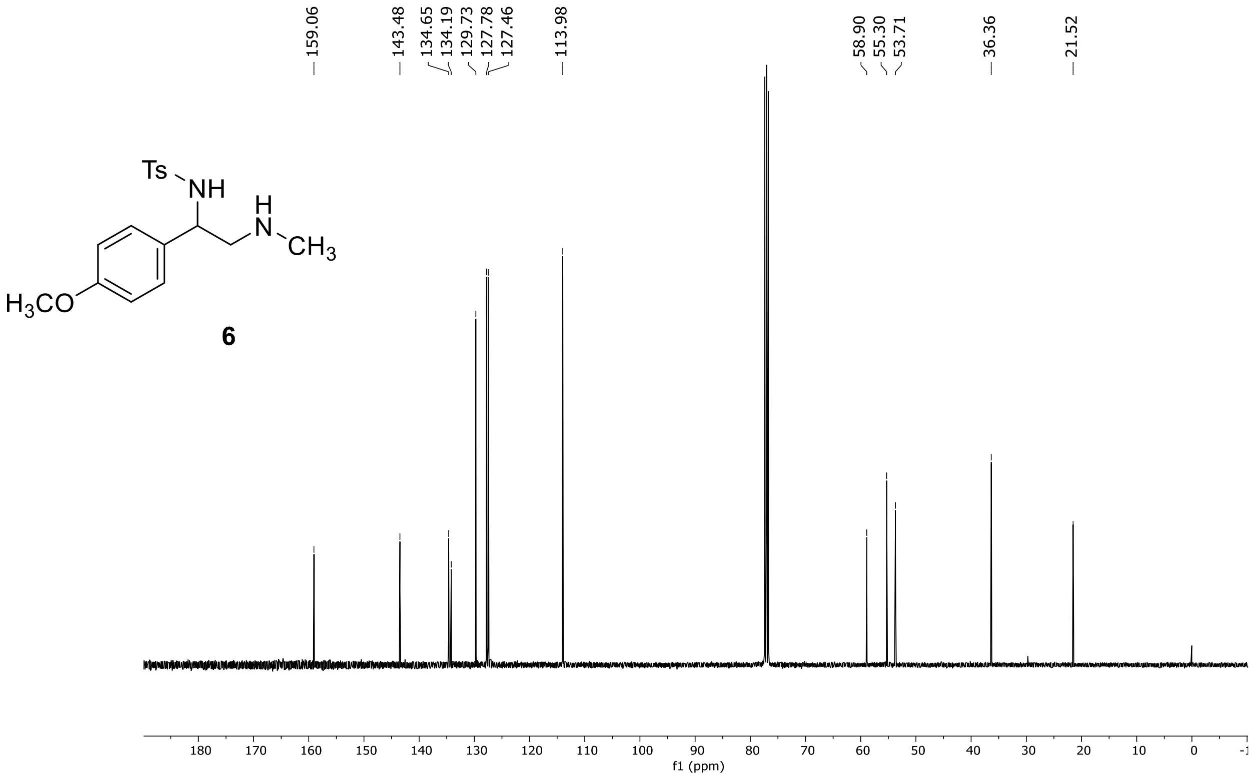

^{1}H NMR spectrum of **7** (In $CDCl_3$, 400 MHz)

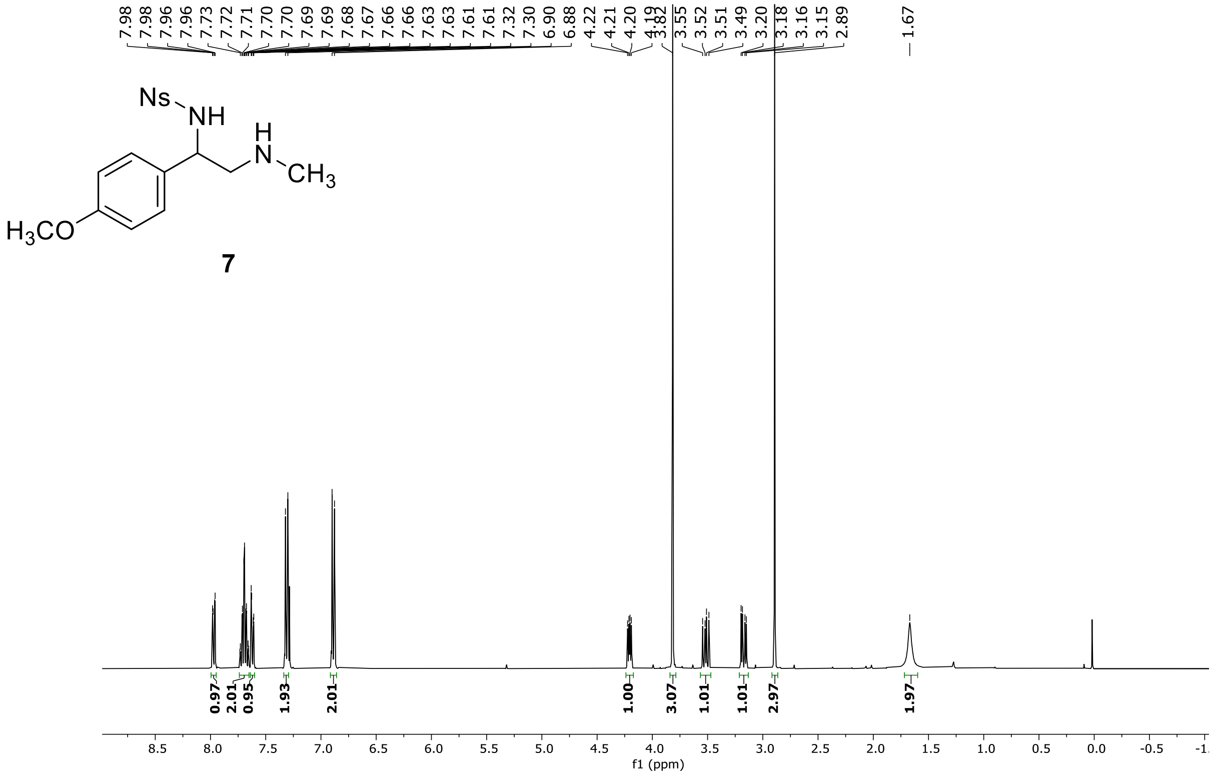

NOESY spectrum of **7** (In $CDCl_3$, 400 MHz)

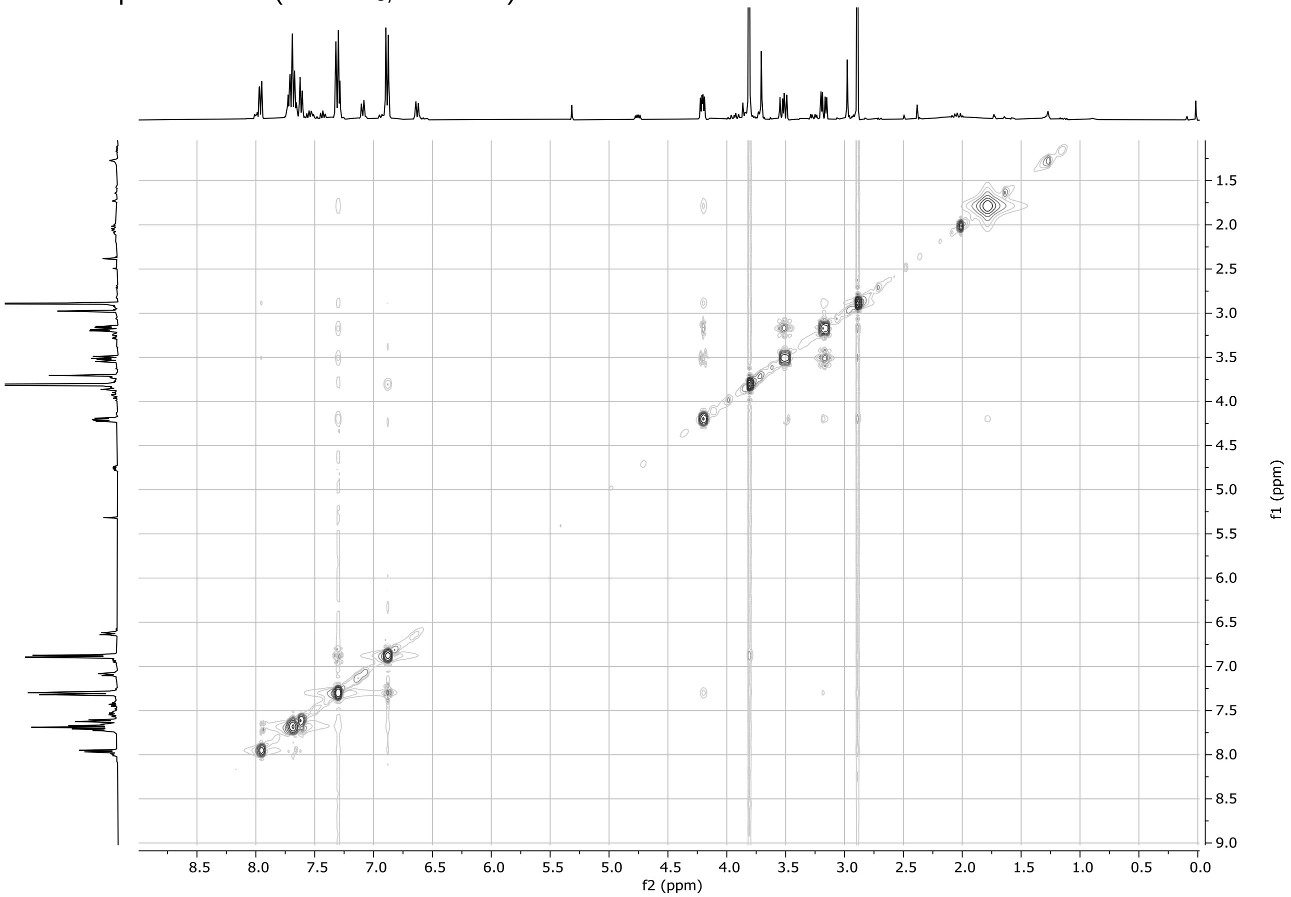

^{13}C NMR spectrum of **7** (In $CDCl_3$, 126 MHz)

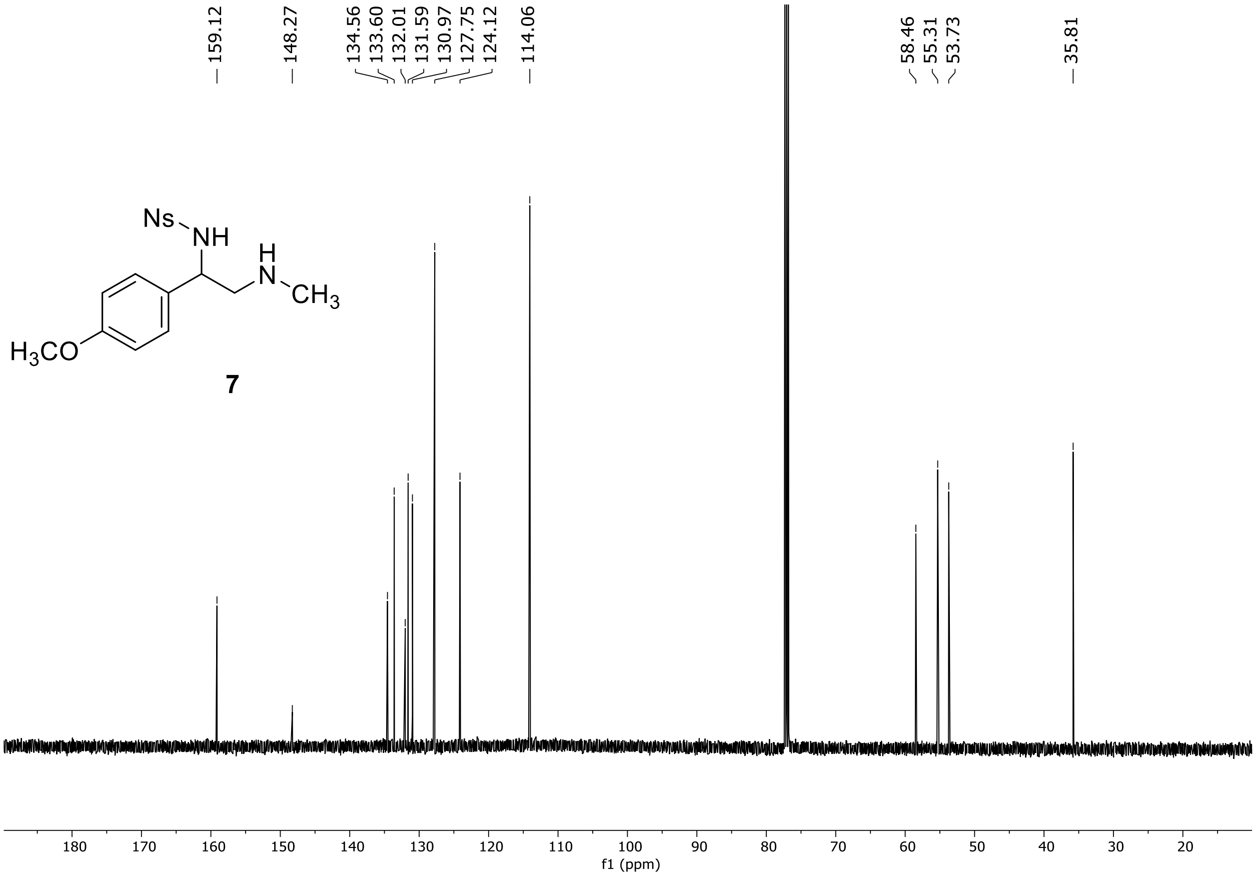

^{1}H NMR spectrum of **8** (In MeOD, 400 MHz)

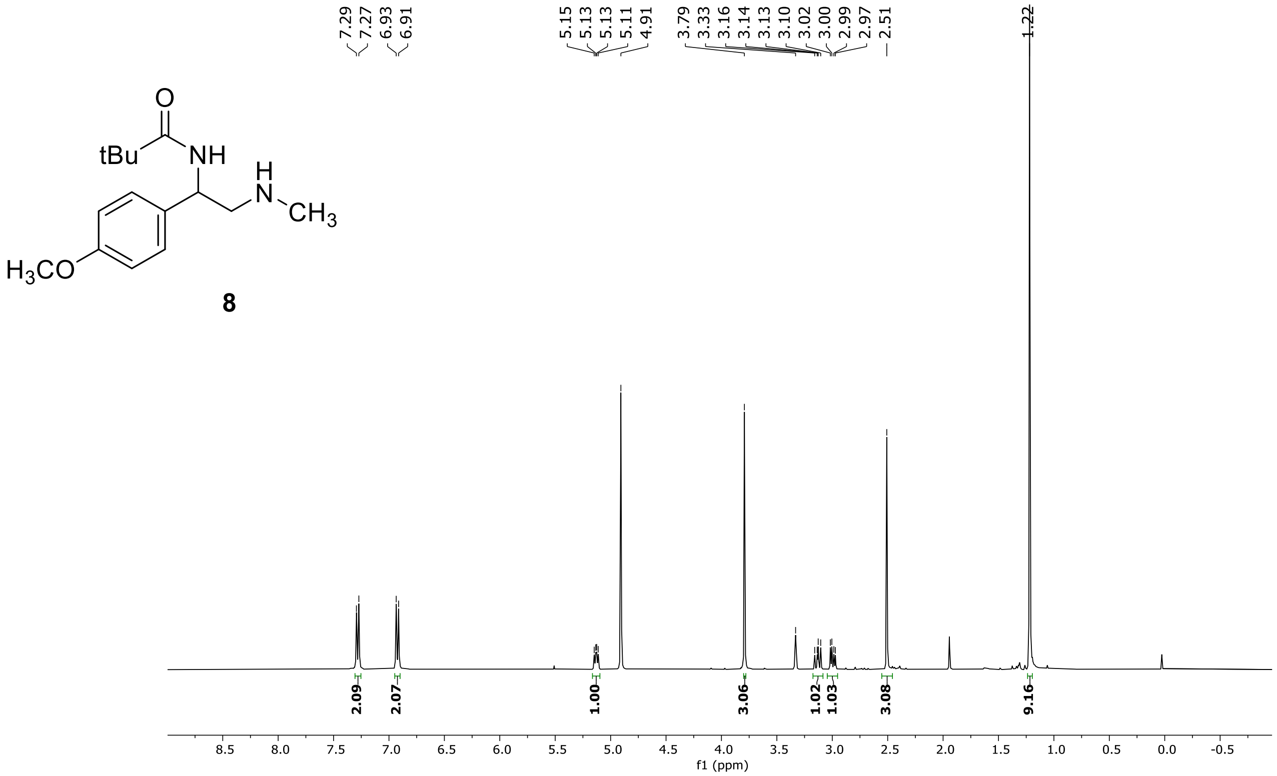

^{13}C NMR spectrum of **8** (In $CDCl_3$, 126 MHz)

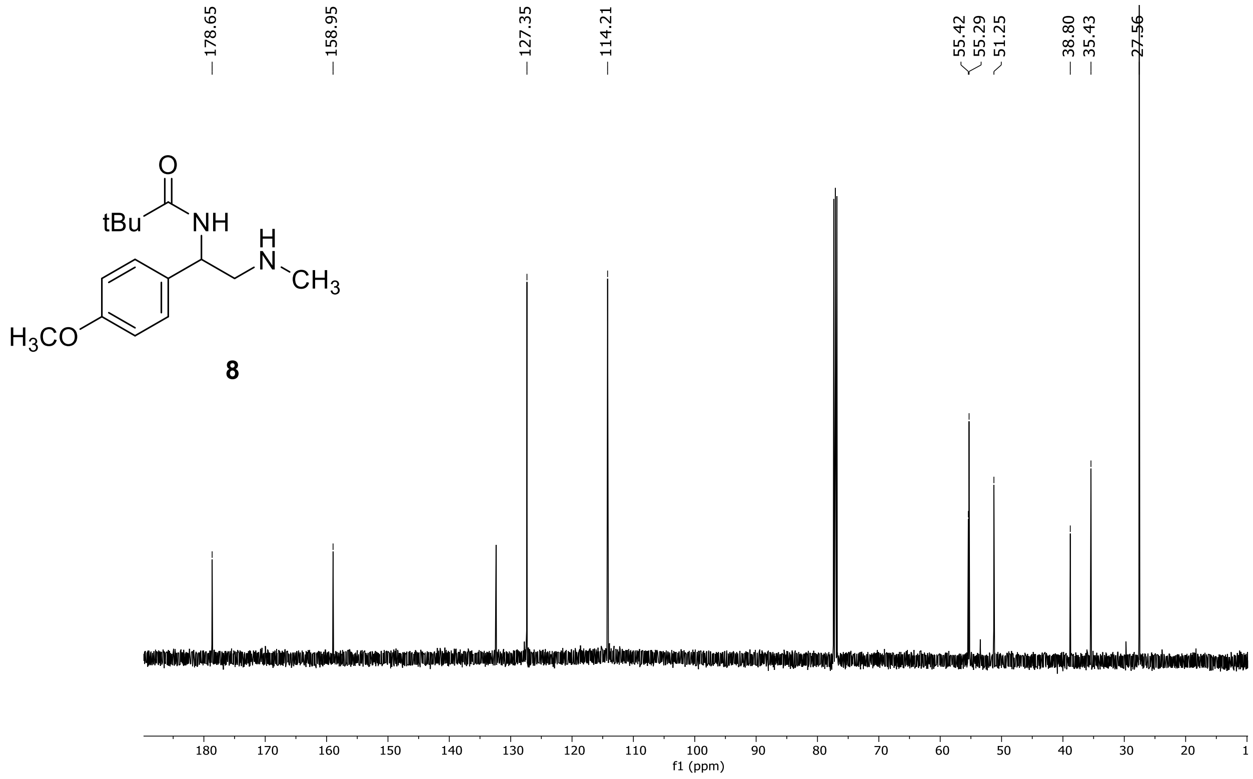

^{1}H NMR spectrum of **9** (In $CDCl_3$, 400 MHz)

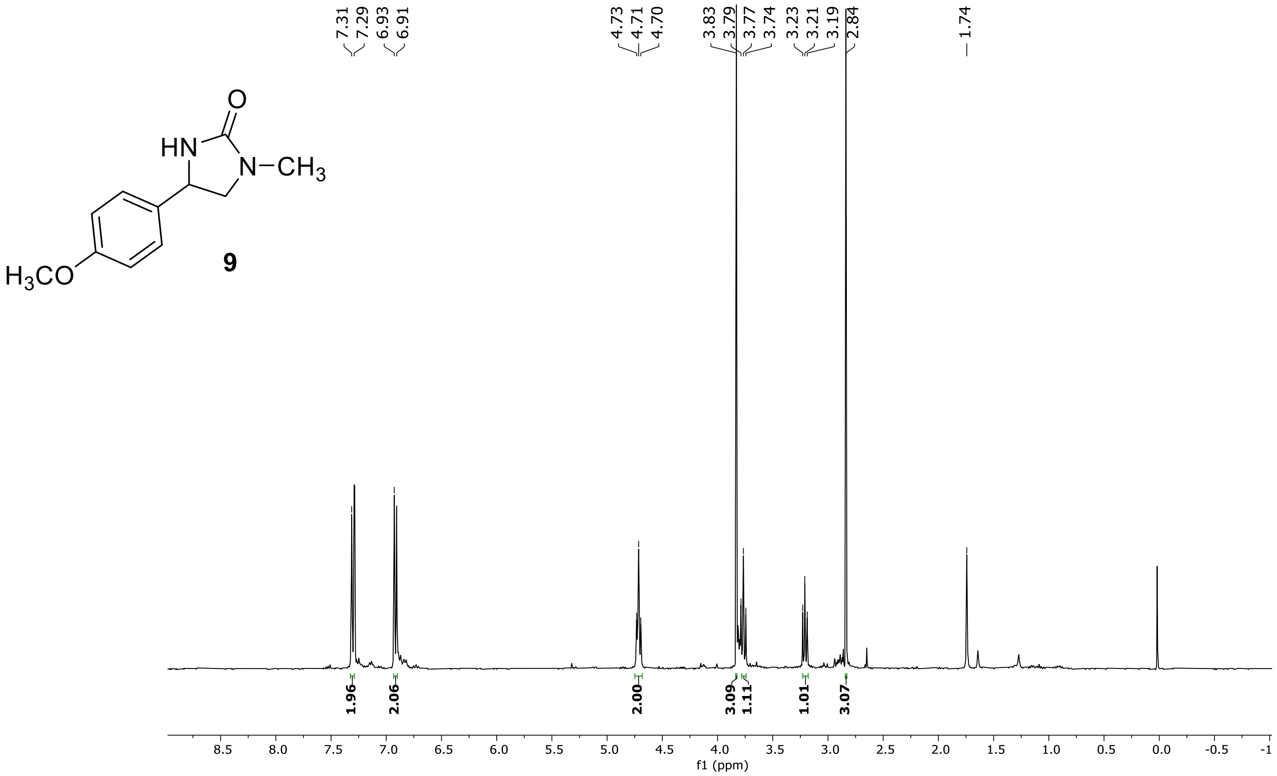

^{13}C NMR spectrum of **9** (In $CDCl_3$, 126 MHz)

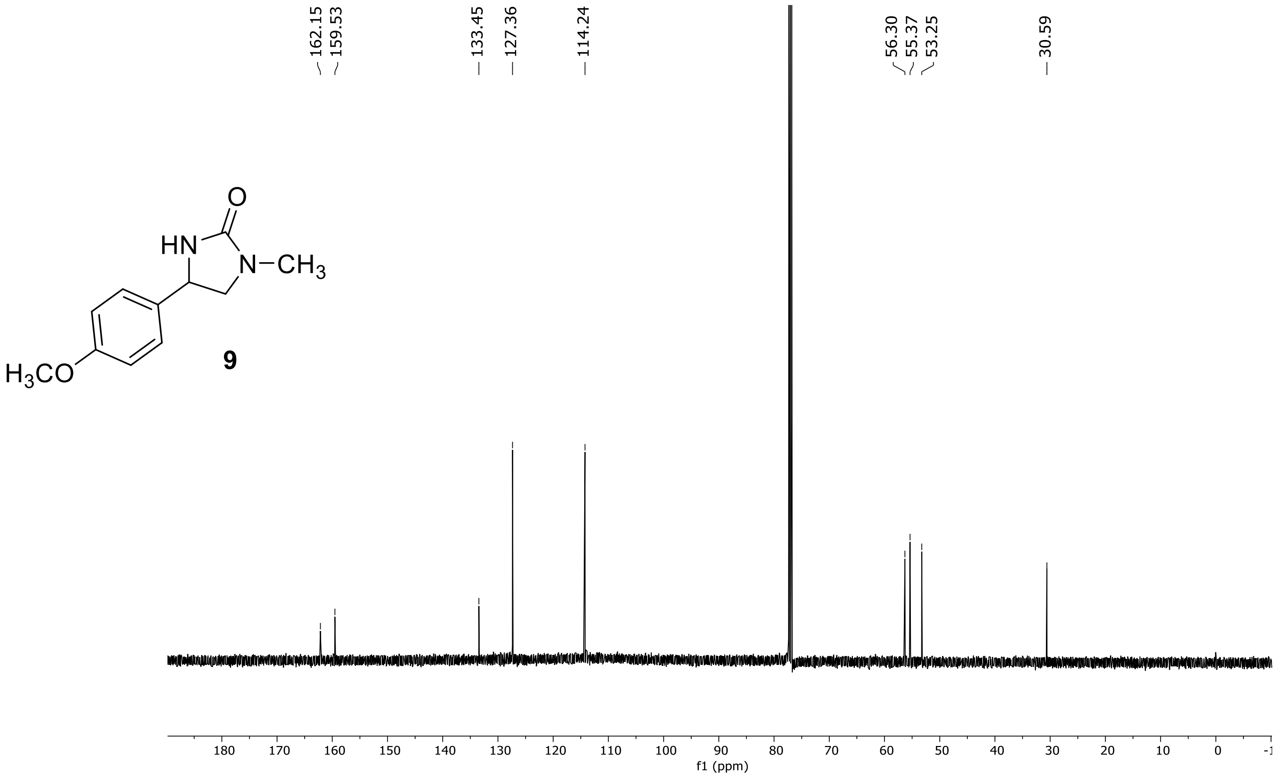

^{1}H NMR spectrum of **10** (In $CDCl_3$, 400 MHz)

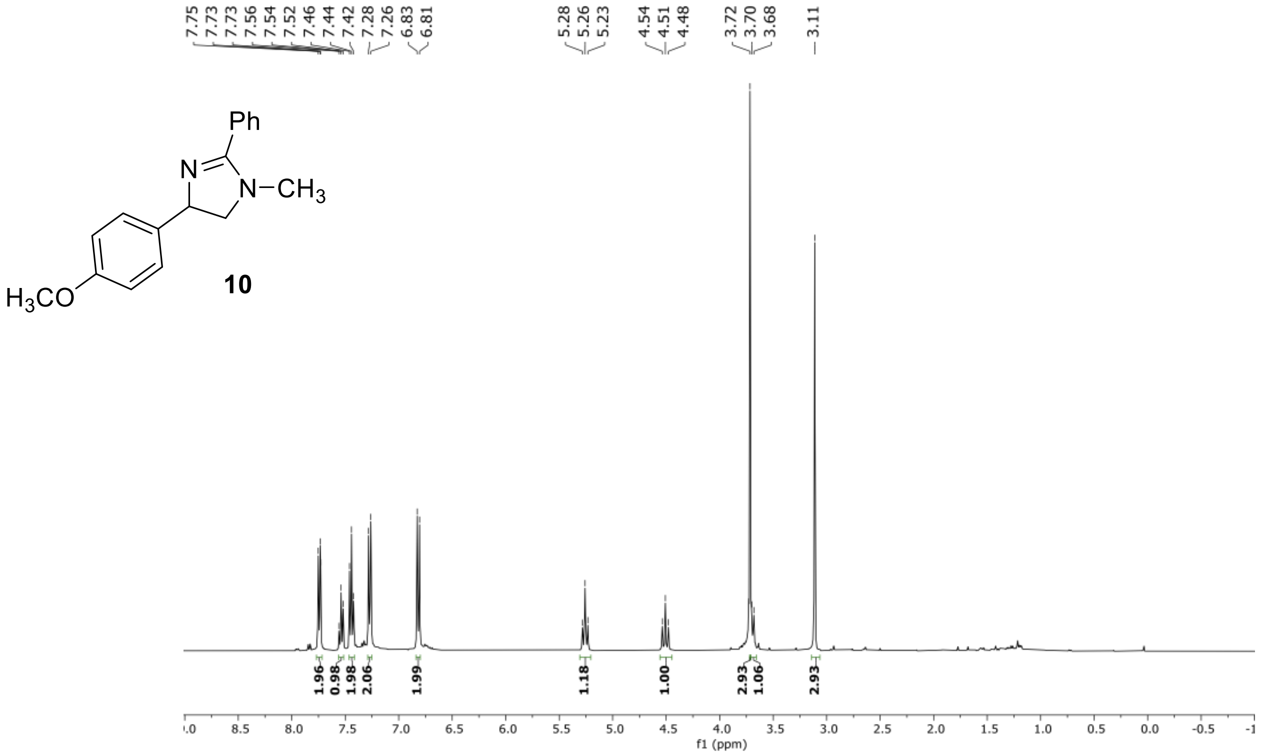

COSY spectrum of **10** (In $CDCl_3$, 400 MHz)

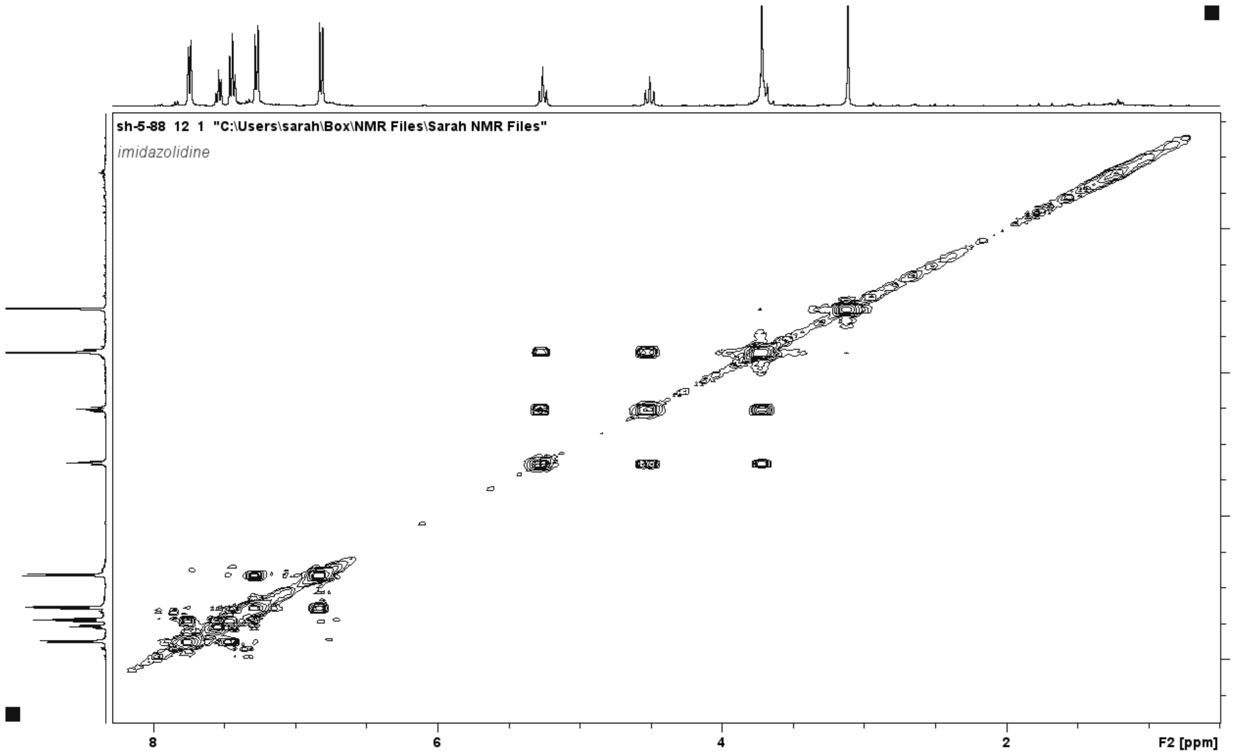

^{13}C NMR spectrum of **10** (In $CDCl_3$, 126 MHz)

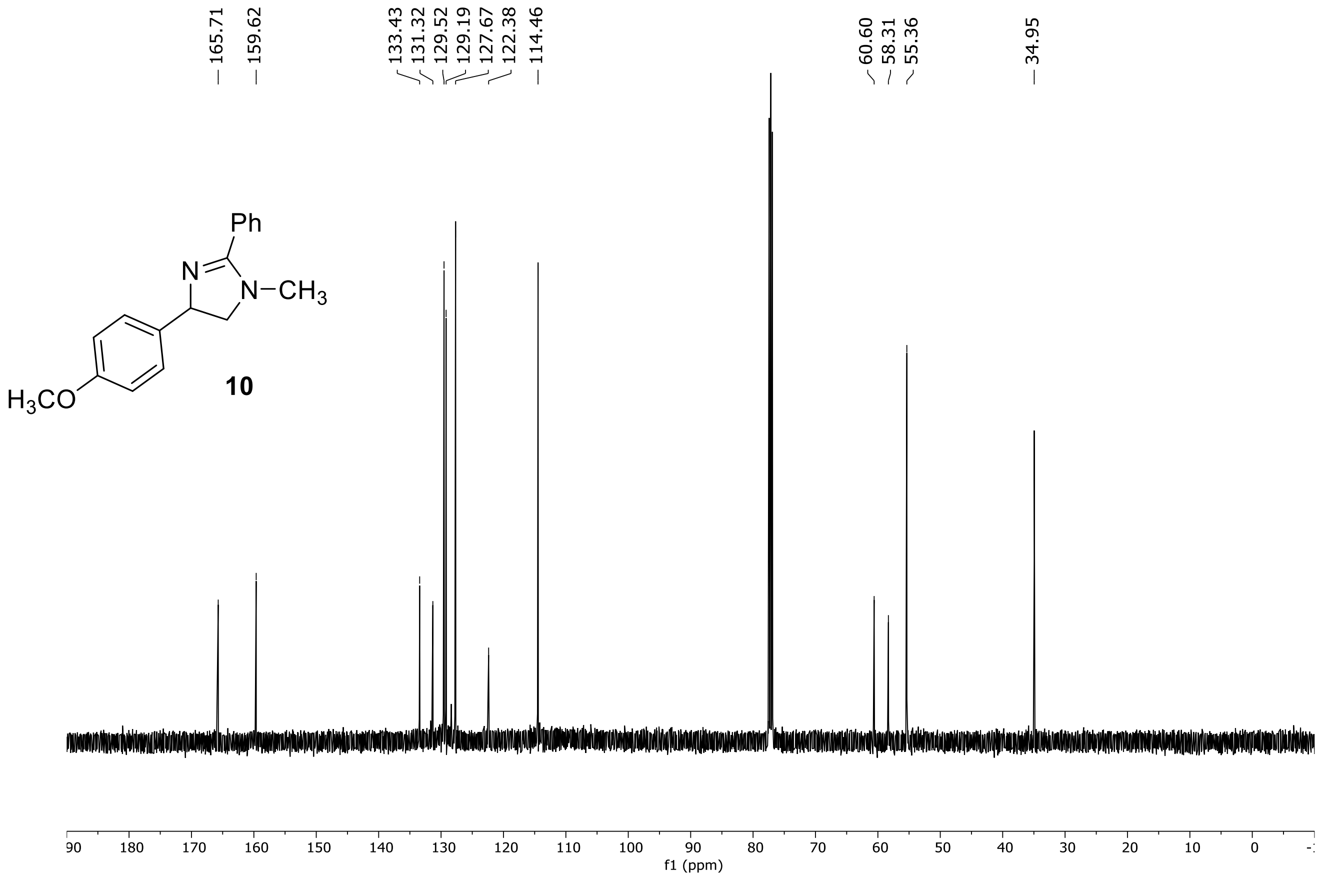

Computational Section General Information

Quantum chemistry methods of meta-hybrid density functional theory (DFT)[14] and second-order Møller-Plesset perturbation theory[15] were carried out at the Center for Computational Sciences (CCS) at Duquesne University using Gaussian 16. The M06-2X functional[16] with Dunning's jul-cc-pVDZ basis set[17] was used primarily to calculate electronic, enthalpic and free energies for both ground and transition structures. M06-2X, developed by Truhlar and co-workers, has been reported to be accurate to within 1.2 kcal/mol for reaction barriers and within 0.37 kcal/mol of non-covalent interaction energies.[16] To verify the accuracy of M06-2X/jul-cc-pDVZ calculations, a subset of activation enthalpies were calculated using expanded basis sets and/or higher levels of theory. Using the M06-2X functional the basis set was increased to a triple zeta (jul-cc-pVTZ) and more diffuse functions (aug-cc-pVDZ). In our previous published paper we verified our theory level, while also using jul-cc-pVDZ, with second-order Møller-Plesset calculations on the first deprotonation transition structure.[18] Vibrational frequency calculations were used to confirm all stationary points as either minima or transition structures and to provide thermodynamic corrections for enthalpies and free energies.

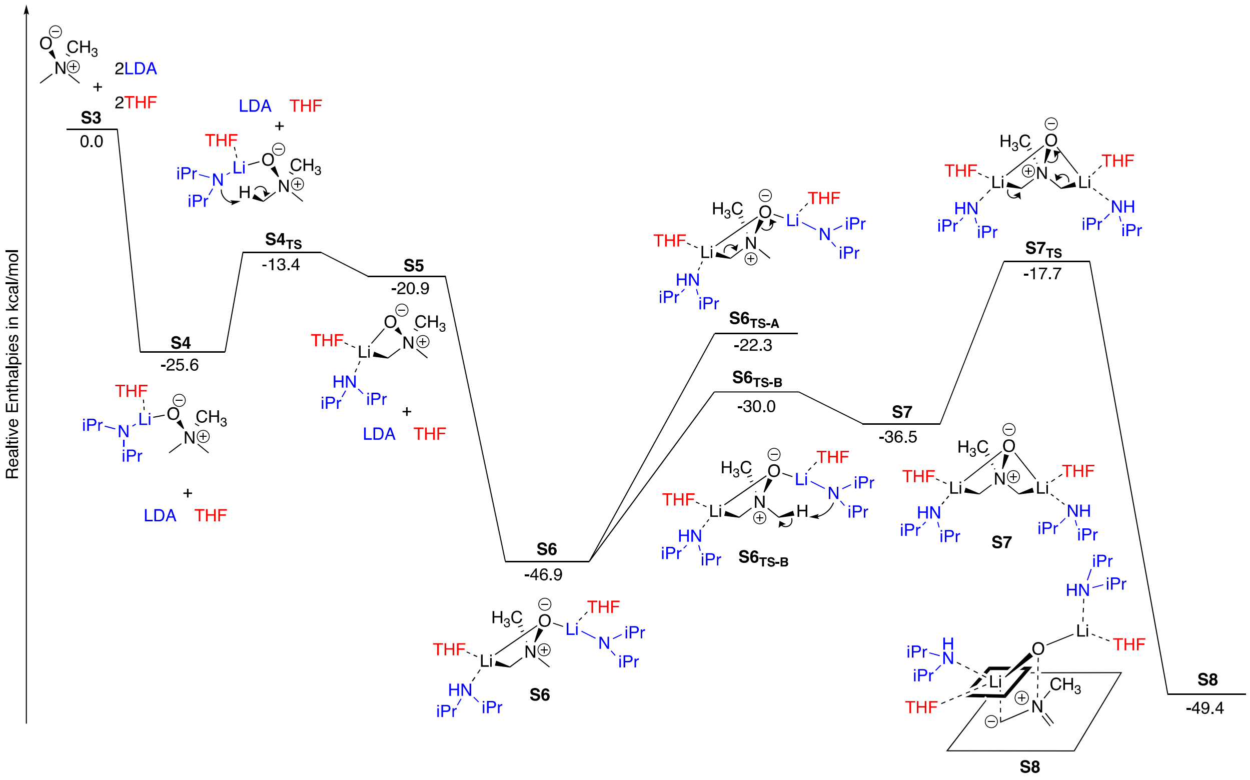

Figure S1. Potential energy surface for trimethylamine *N*-oxide conversion to 1,3-dipole.

Computational Outputs

Ground Structure S3

Input:

#m062x/jul-cc-pvDz opt=(calcfc,noeigen,z-matrix) freq scrf=(pcm,solvent=THF)

Optimized Structure:

Center Number	Atomic Number	Atomic Type	Coordinates (Angstroms) X	Y	Z
1	7	0	0.000000	0.000000	0.000000
2	6	0	0.000000	0.000000	1.488770
3	6	0	1.404307	0.000000	-0.494042
4	6	0	-0.696267	-1.220373	-0.492524
5	8	0	-0.648419	1.115684	-0.459017
6	1	0	0.508955	0.911931	1.811829
7	1	0	-1.044405	0.019344	1.810614
8	1	0	0.512556	-0.891415	1.869480
9	1	0	1.362405	0.008495	-1.586368
10	1	0	1.874181	0.917119	-0.129537
11	1	0	1.936053	-0.886439	-0.128405
12	1	0	-1.723111	-1.178693	-0.119908
13	1	0	-0.691648	-1.175889	-1.584667
14	1	0	-0.184461	-2.121348	-0.133909

Thermochemistry:

Zero-point correction=	0.126133 (Hartree/Particle)
Thermal correction to Energy=	0.132168
Thermal correction to Enthalpy=	0.133113
Thermal correction to Gibbs Free Energy=	0.09765
Sum of electronic and zero-point Energies=	-249.42378
Sum of electronic and thermal Energies=	-249.417745
Sum of electronic and thermal Enthalpies=	-249.416801
Sum of electronic and thermal Free Energies=	-249.452264

Lithium Diisopropylamine

Input:

#m062x/jul-cc-pvDz opt=(calcfc,noeigen,z-matrix) optcyc=50 freq scrf=(pcm,solvent=THF)

Optimized Structure:

Center Number	Atomic Number	Atomic Type	Coordinates (Angstroms) X	Y	Z
1	7	0	0.000000	0.000000	0.000000
2	6	0	0.000000	0.000000	1.447071
3	6	0	1.320503	0.000000	-0.591980
4	1	0	0.458495	-0.927914	1.862649
5	6	0	0.768905	1.171075	2.082136
6	6	0	-1.443593	0.021706	1.949140
7	1	0	1.887016	0.928264	-0.344298
8	6	0	2.215060	-1.170471	-0.149593
9	6	0	1.187867	-0.022790	-2.114634
10	1	0	1.837749	1.138792	1.831358
11	1	0	0.361049	2.126427	1.715305
12	1	0	0.681715	1.150932	3.178982
13	1	0	-1.494270	-0.036811	3.045388
14	1	0	-1.936474	0.955634	1.634161
15	1	0	-2.007571	-0.828262	1.536791
16	1	0	3.180264	-1.150058	-0.677838
17	1	0	2.423430	-1.137607	0.928296
18	1	0	1.714022	-2.126205	-0.371300
19	1	0	2.167370	0.034681	-2.609611
20	1	0	0.698334	-0.956811	-2.434644
21	1	0	0.581331	0.827124	-2.461484
22	3	0	-1.564354	-0.006481	-1.017643

Thermochemistry:

Zero-point correction=	0.193369 (Hartree/Particle)
Thermal correction to Energy=	0.202911
Thermal correction to Enthalpy=	0.203856
Thermal correction to Gibbs Free Energy=	0.160086
Sum of electronic and zero-point Energies=	-299.06429
Sum of electronic and thermal Energies=	-299.054748
Sum of electronic and thermal Enthalpies=	-299.053803
Sum of electronic and thermal Free Energies=	-299.097573

Tetrahydrofuran

Input:

#m062x/jul-cc-pvDz opt=(calcfc,noeigen,z-matrix) optcyc=50 freq scrf=(pcm,solvent=THF)

Optimized Structure:

Center Number	Atomic Number	Atomic Type	Coordinates (Angstroms) X	Y	Z
1	8	0	0.000000	0.000000	0.000000
2	6	0	0.000000	0.000000	1.423444
3	6	0	1.480680	0.000000	1.831594
4	6	0	2.191048	-0.619943	0.604432
5	6	0	1.037814	-0.901083	-0.370609
6	1	0	-0.558030	0.878751	1.763616
7	1	0	-0.505324	-0.910144	1.789899
8	1	0	1.831979	1.022586	2.009101
9	1	0	1.644570	-0.577562	2.748069
10	1	0	2.902471	0.089311	0.166947
11	1	0	2.737597	-1.536064	0.854193
12	1	0	1.286342	-0.719839	-1.421658
13	1	0	0.678591	-1.938757	-0.260513

Thermochemistry:

Zero-point correction=	0.117371 (Hartree/Particle)
Thermal correction to Energy=	0.122252
Thermal correction to Enthalpy=	0.123197
Thermal correction to Gibbs Free Energy=	0.089034
Sum of electronic and zero-point Energies=	-232.255132
Sum of electronic and thermal Energies=	-232.250251
Sum of electronic and thermal Enthalpies=	-232.249307
Sum of electronic and thermal Free Energies=	-232.28347

Ground Structure S4

Input:

#m062x/jul-cc-pvDz opt=(calcfc,noeigen,z-matrix) freq optcyc=50 scrf=(pcm,solvent=THF)

Optimized Structure:

Center Number	Atomic Number	Atomic Type	Coordinates (Angstroms) X	Y	Z
1	7	0	0.000000	0.000000	0.000000
2	6	0	0.000000	0.000000	1.486000

3	6	0	1.401699	0.000000	-0.510019
4	6	0	-0.707702	-1.206463	-0.507697
5	8	0	-0.658956	1.125948	-0.437459
6	1	0	0.525919	-0.886570	1.857131
7	1	0	0.500136	0.916314	1.809511
8	1	0	-1.043140	0.003165	1.813336
9	1	0	1.913514	-0.905140	-0.162701
10	1	0	1.334140	0.039203	-1.603637
11	1	0	1.888655	0.895987	-0.115275
12	1	0	-0.210868	-2.113061	-0.144063
13	1	0	-1.739409	-1.153519	-0.149516
14	1	0	-0.674869	-1.154510	-1.599965
15	3	0	-0.791984	1.430874	-2.303940
16	8	0	-2.648881	2.008044	-2.460407
17	7	0	0.365954	0.835135	-3.696440
18	6	0	1.684009	1.428283	-3.714353
19	6	0	0.224725	-0.307311	-4.568217
20	1	0	2.461000	0.767438	-3.244802
21	6	0	2.235840	1.759903	-5.111120
22	6	0	1.658961	2.705707	-2.872833
23	1	0	0.483045	-0.060262	-5.623437
24	6	0	1.118496	-1.503652	-4.189963
25	6	0	-1.237742	-0.747112	-4.594969
26	1	0	1.508964	2.376386	-5.663097
27	1	0	3.180531	2.318368	-5.031559
28	1	0	2.432727	0.854309	-5.700153
29	1	0	2.660249	3.149557	-2.776273
30	1	0	0.994868	3.446666	-3.344405
31	1	0	1.278545	2.501063	-1.861089
32	1	0	1.007301	-2.331473	-4.906977
33	1	0	2.179229	-1.216273	-4.173506
34	1	0	0.853849	-1.877440	-3.188080
35	1	0	-1.383273	-1.641056	-5.218684
36	1	0	-1.589562	-0.979148	-3.576748
37	1	0	-1.864794	0.060918	-4.992965
38	6	0	-3.626622	1.478395	-1.560827
39	6	0	-4.345878	0.420626	-2.390229
40	6	0	-4.366330	1.047886	-3.799829
41	6	0	-3.304318	2.165044	-3.725378
42	1	0	-3.088947	1.101538	-0.685891
43	1	0	-4.315604	2.285078	-1.260333
44	1	0	-3.761357	-0.508028	-2.388387
45	1	0	-5.348706	0.199336	-2.009095
46	1	0	-4.125327	0.309343	-4.571655
47	1	0	-5.350169	1.469921	-4.032812
48	1	0	-2.532201	2.103508	-4.499523
49	1	0	-3.772305	3.159957	-3.759053

Thermochemistry:

Zero-point correction=	0.440969 (Hartree/Particle)
Thermal correction to Energy=	0.463945
Thermal correction to Enthalpy=	0.46489
Thermal correction to Gibbs Free Energy=	0.389236
Sum of electronic and zero-point Energies=	-780.783657
Sum of electronic and thermal Energies=	-780.76068
Sum of electronic and thermal Enthalpies=	-780.759736
Sum of electronic and thermal Free Energies=	-780.83539

Transition Structure $S4_{TS}$

Input:

#m062x/jul-cc-pvDz opt=(calcfc,noeigen,z-matrix,ts) freq optcyc=50 scrf=(pcm,solvent=THF)

Optimized Structure:

Center Number	Atomic Number	Atomic Type	Coordinates (Angstroms) X	Y	Z
1	7	0	0.000000	0.000000	0.000000
2	6	0	0.000000	0.000000	1.510708
3	6	0	1.376848	0.000000	-0.558086
4	6	0	-0.707613	-1.208437	-0.493689
5	8	0	-0.653997	1.116819	-0.507843
6	1	0	-1.212485	0.618094	2.000790
7	1	0	0.269787	-1.023604	1.817133
8	1	0	0.833490	0.659826	1.799865
9	1	0	1.309204	-0.022503	-1.651349
10	1	0	1.855539	0.925772	-0.229023
11	1	0	1.920781	-0.871604	-0.178241
12	1	0	-1.715582	-1.194066	-0.073511
13	1	0	-0.750292	-1.147355	-1.584748
14	1	0	-0.172350	-2.108490	-0.171336
15	3	0	-2.218454	1.553969	0.366690
16	-1	0	-0.940821	0.479607	2.847560
17	7	0	-2.282874	1.314788	2.319716
18	6	0	-1.879411	2.480704	3.103273
19	6	0	-3.226085	0.437019	2.999495
20	1	0	-2.755778	3.133328	3.292654
21	6	0	-1.276334	2.135883	4.472854
22	6	0	-0.879149	3.313455	2.299978
23	1	0	-2.791715	0.030440	3.937554
24	6	0	-4.534904	1.135317	3.379842
25	6	0	-3.516620	-0.765704	2.104150
26	1	0	-0.431035	1.442783	4.347041
27	1	0	-0.908990	3.043724	4.972220
28	1	0	-2.011835	1.664069	5.137078

29	1	0	-0.662306	4.259909	2.813939
30	1	0	0.063611	2.767660	2.165459
31	1	0	-1.271475	3.548829	1.301619
32	1	0	-5.238572	0.419386	3.828307
33	1	0	-4.373393	1.943341	4.105143
34	1	0	-5.002375	1.567465	2.481551
35	1	0	-4.238280	-1.444438	2.578708
36	1	0	-3.940518	-0.432755	1.143269
37	1	0	-2.595971	-1.327778	1.904657
38	8	0	-3.094990	2.979450	-0.569058
39	6	0	-2.435957	3.541144	-1.710305
40	6	0	-1.804397	4.822205	-1.179991
41	6	0	-2.839927	5.314584	-0.149493
42	6	0	-3.677861	4.060291	0.176187
43	1	0	-1.717436	2.796250	-2.067874
44	1	0	-3.182570	3.752209	-2.492431
45	1	0	-0.849525	4.585916	-0.694960
46	1	0	-1.617610	5.553693	-1.973228
47	1	0	-2.358741	5.722520	0.746012
48	1	0	-3.476338	6.097721	-0.575736
49	1	0	-3.650061	3.770688	1.233571
50	1	0	-4.724264	4.182573	-0.136062

Thermochemistry:

Zero-point correction=	0.436674 (Hartree/Particle)
Thermal correction to Energy=	0.459885
Thermal correction to Enthalpy=	0.460829
Thermal correction to Gibbs Free Energy=	0.384031
Sum of electronic and zero-point Energies=	-780.764477
Sum of electronic and thermal Energies=	-780.741266
Sum of electronic and thermal Enthalpies=	-780.740322
Sum of electronic and thermal Free Energies=	-780.81712

Ground Structure S5

Input:

#m062x/jul-cc-pvDz opt=(calcfc,noeigen,z-matrix) freq optcyc=50 SCF=XQC scrf=(pcm,solvent=THF,read)

Optimized Structure:

Center Number	Atomic Number	Atomic Type	Coordinates (Angstroms)		
			X	Y	Z
1	7	0	0.000000	0.000000	0.000000
2	6	0	0.000000	0.000000	1.518924
3	6	0	1.398771	0.000000	-0.491766
4	6	0	-0.720521	-1.152025	-0.596598

5	8	0	-0.619899	1.166818	-0.454029
6	1	0	-2.064810	2.958030	2.813834
7	1	0	-1.016675	-0.344967	1.783237
8	1	0	0.689352	-0.808145	1.818745
9	1	0	1.373813	0.119193	-1.579726
10	1	0	1.902703	0.848310	-0.021438
11	1	0	1.887673	-0.940892	-0.213401
12	1	0	-1.755537	-1.096239	-0.249618
13	1	0	-0.684244	-1.066859	-1.689144
14	1	0	-0.252772	-2.083992	-0.259056
15	3	0	-0.246336	2.080308	1.168970
16	-1	0	-1.890768	2.708699	3.660914
17	7	0	-1.595397	3.430099	2.039698
18	6	0	-0.812099	4.545573	2.610529
19	6	0	-2.625626	3.792068	1.048089
20	1	0	-0.106030	4.868739	1.831686
21	6	0	-1.649168	5.747824	3.046814
22	6	0	-0.011113	3.994756	3.784919
23	1	0	-3.283587	4.581686	1.449136
24	6	0	-1.964495	4.304129	-0.226091
25	6	0	-3.478908	2.560825	0.767337
26	1	0	-2.441448	5.432932	3.742203
27	1	0	-1.009650	6.475003	3.563948
28	1	0	-2.116763	6.258720	2.196542
29	1	0	0.711812	4.737895	4.143588
30	1	0	-0.685042	3.746167	4.618695
31	1	0	0.530223	3.082816	3.502706
32	1	0	-2.733707	4.583393	-0.957695
33	1	0	-1.343323	5.189692	-0.037010
34	1	0	-1.338697	3.513144	-0.663513
35	1	0	-4.276457	2.810294	0.055824
36	1	0	-2.853302	1.764858	0.341483
37	1	0	-3.950633	2.195539	1.690674
38	8	0	1.356163	3.160414	0.766779
39	6	0	1.562315	3.617180	-0.574232
40	6	0	3.069947	3.459912	-0.848712
41	6	0	3.682282	3.177396	0.540943
42	6	0	2.534092	3.479691	1.502785
43	1	0	0.923023	3.000992	-1.215050
44	1	0	1.252172	4.671977	-0.643873
45	1	0	3.263263	2.634109	-1.542136
46	1	0	3.479777	4.372515	-1.294356
47	1	0	3.973683	2.124329	0.630792
48	1	0	4.564178	3.792098	0.749369
49	1	0	2.541817	2.864878	2.408561
50	1	0	2.513347	4.546308	1.785152

Thermochemistry:

Zero-point correction=	0.442683 (Hartree/Particle)
Thermal correction to Energy=	0.466264
Thermal correction to Enthalpy=	0.467208
Thermal correction to Gibbs Free Energy=	0.389854
Sum of electronic and zero-point Energies=	-780.776752
Sum of electronic and thermal Energies=	-780.753171
Sum of electronic and thermal Enthalpies=	-780.752227
Sum of electronic and thermal Free Energies=	-780.829581

Ground Structure S6

Input:

#m062x/jul-cc-pvDz opt=(calcfc,noeigen,z-matrix) freq optcyc=50 scrf=(pcm,solvent=THF,read)

Optimized Structure:

Center Number	Atomic Number	Atomic Type	Coordinates (Angstroms) X	Y	Z
1	7	0	0.157521	-0.128925	2.323818
2	6	0	-1.254362	-0.155415	2.874067
3	6	0	0.725960	1.233925	2.487950
4	6	0	1.064226	-1.120089	2.956262
5	8	0	0.114529	-0.435507	0.954856
6	1	0	-1.431395	-1.215806	3.131507
7	1	0	-1.209049	0.393347	3.828534
8	1	0	1.664236	1.290285	1.920731
9	1	0	-0.007961	1.935812	2.081341
10	1	0	0.881570	1.426111	3.555164
11	1	0	0.623680	-2.107693	2.795753
12	1	0	2.048678	-1.061280	2.477565
13	1	0	1.134204	-0.905306	4.028108
14	3	0	1.598624	-0.010019	-0.156116
15	3	0	-1.773880	-0.078656	0.826758
16	7	0	-3.236487	-1.468069	0.227743
17	6	0	-2.586307	-2.612625	-0.435225
18	6	0	-4.327270	-0.798612	-0.509173
19	1	0	-3.340430	-3.309996	-0.835979
20	6	0	-1.715228	-2.122092	-1.586063
21	6	0	-1.765050	-3.358262	0.609646
22	1	0	-3.859042	-0.188340	-1.294377
23	6	0	-5.324122	-1.749717	-1.171186
24	6	0	-5.041951	0.132626	0.464523
25	1	0	-0.906883	-1.479151	-1.206016
26	1	0	-1.258157	-2.975991	-2.102941
27	1	0	-2.303069	-1.561348	-2.324851
28	1	0	-1.237140	-4.202673	0.148392

29	1	0	-1.030217	-2.675654	1.056424
30	1	0	-2.414485	-3.752950	1.403429
31	1	0	-6.158652	-1.173858	-1.591917
32	1	0	-4.870809	-2.325050	-1.987113
33	1	0	-5.731851	-2.454548	-0.431607
34	1	0	-5.769155	0.761314	-0.064851
35	1	0	-5.588934	-0.457520	1.215245
36	1	0	-4.328155	0.780506	0.987924
37	8	0	1.888616	-1.648604	-1.147918
38	7	0	2.937343	1.356829	-0.210605
39	6	0	2.458244	2.715004	-0.263017
40	6	0	4.284998	1.252222	0.301471
41	1	0	2.417820	3.163933	0.768443
42	6	0	3.297698	3.705728	-1.091421
43	6	0	1.028441	2.721718	-0.802233
44	1	0	4.526496	2.132169	0.942257
45	6	0	4.411192	0.010064	1.191713
46	6	0	5.385190	1.177948	-0.779255
47	1	0	3.362481	3.361092	-2.135863
48	1	0	2.838941	4.706922	-1.079452
49	1	0	4.317147	3.799176	-0.695193
50	1	0	0.598998	3.735362	-0.797235
51	1	0	1.030174	2.356526	-1.842028
52	1	0	0.371355	2.068694	-0.208770
53	1	0	5.455113	-0.211983	1.460698
54	1	0	3.836020	0.138484	2.118124
55	1	0	4.004698	-0.858103	0.648761
56	1	0	6.392961	1.227539	-0.335384
57	1	0	5.301813	0.223251	-1.321955
58	1	0	5.284759	1.988905	-1.509189
59	6	0	1.654575	-2.931330	-0.566927
60	6	0	3.049209	-3.454584	-0.231708
61	6	0	3.931245	-2.850911	-1.346782
62	6	0	3.035369	-1.777619	-1.997241
63	1	0	1.000127	-2.777583	0.295457
64	1	0	1.151163	-3.578166	-1.304999
65	1	0	3.358467	-3.082312	0.752747
66	1	0	3.085152	-4.548963	-0.209466
67	1	0	4.848119	-2.412171	-0.937027
68	1	0	4.222422	-3.608057	-2.082740
69	1	0	3.494263	-0.785471	-2.056516
70	1	0	2.700978	-2.091985	-2.997163
71	8	0	-2.134552	1.600788	-0.125349
72	6	0	-2.495293	2.781945	0.596362
73	6	0	-3.658765	3.407521	-0.194443
74	6	0	-3.577771	2.724128	-1.579878
75	6	0	-2.266953	1.940091	-1.505508
76	1	0	-2.745802	2.479093	1.618700
77	1	0	-1.625644	3.457600	0.623541
78	1	0	-4.619869	3.204121	0.289669

79	1	0	-3.540729	4.493721	-0.265200
80	1	0	-4.422985	2.040982	-1.725182
81	1	0	-3.573637	3.442361	-2.406235
82	1	0	-2.247965	1.012436	-2.087678
83	1	0	-1.414185	2.567997	-1.804566
84	1	0	-3.626906	-1.801996	1.110036

Thermochemistry:

Zero-point correction=	0.757492 (Hartree/Particle)
Thermal correction to Energy=	0.798624
Thermal correction to Enthalpy=	0.799568
Thermal correction to Gibbs Free Energy=	0.683725
Sum of electronic and zero-point Energies=	-1312.137906
Sum of electronic and thermal Energies=	-1312.096775
Sum of electronic and thermal Enthalpies=	-1312.095831
Sum of electronic and thermal Free Energies=	-1312.211674

Transition Structure S6$_{TS-A}$

Input:

#m062x/jul-cc-pvDz opt=(calcfc,noeigen,z-matrix,ts) freq optcyc=50 SCF=XQC scrf=(pcm,solvent=THF,read)

Optimized Structure:

Center Number	Atomic Number	Atomic Type	Coordinates (Angstroms) X	Y	Z
1	7	0	-0.166861	-0.095772	2.307173
2	6	0	1.233569	-0.057233	2.892462
3	6	0	-0.805575	-1.461786	2.363580
4	6	0	-1.072932	0.888293	2.943206
5	8	0	-0.051608	0.307338	0.952575
6	1	0	3.395069	0.825165	-0.975254
7	1	0	1.378344	0.983730	3.242711
8	1	0	1.190373	-0.687311	3.794413
9	1	0	-1.798317	-1.653852	1.209060
10	1	0	0.034349	-2.157518	2.204973
11	1	0	-1.125008	-1.588155	3.413767
12	1	0	-0.629962	1.881036	2.816839
13	1	0	-2.053487	0.843567	2.454765
14	1	0	-1.165543	0.646964	4.007546
15	3	0	-1.497396	-0.031282	-0.111674
16	3	0	1.769087	-0.159554	0.836575
17	7	0	2.622315	1.258488	-0.465205
18	6	0	3.158913	2.434449	0.249110
19	6	0	1.544563	1.543058	-1.432757

20	1	0	3.656310	3.120389	-0.459405
21	6	0	2.032837	3.188797	0.949565
22	6	0	4.197026	1.949694	1.254185
23	1	0	0.684810	1.873812	-0.838462
24	6	0	1.897771	2.614718	-2.462927
25	6	0	1.148744	0.243106	-2.120238
26	1	0	1.411293	2.482281	1.515950
27	1	0	2.459543	3.929134	1.637934
28	1	0	1.391741	3.722812	0.236795
29	1	0	4.695365	2.803586	1.730285
30	1	0	3.709145	1.343940	2.031163
31	1	0	4.967377	1.339634	0.761258
32	1	0	1.058992	2.754062	-3.157967
33	1	0	2.109590	3.584065	-1.995647
34	1	0	2.779242	2.312002	-3.047969
35	1	0	0.310687	0.419298	-2.805725
36	1	0	1.989192	-0.160661	-2.705767
37	1	0	0.841960	-0.511162	-1.383716
38	8	0	-1.875622	1.573639	-1.103201
39	7	0	-2.500343	-1.702360	0.174400
40	6	0	-2.071050	-2.777920	-0.722234
41	6	0	-3.894719	-1.810843	0.585712
42	1	0	-2.770325	-3.632821	-0.638373
43	6	0	-2.048116	-2.335624	-2.192256
44	6	0	-0.678995	-3.287090	-0.335389
45	1	0	-4.059694	-2.789235	1.089510
46	6	0	-4.207531	-0.714196	1.600517
47	6	0	-4.895635	-1.740399	-0.570370
48	1	0	-1.276258	-1.562284	-2.333949
49	1	0	-1.805157	-3.178192	-2.857034
50	1	0	-3.011090	-1.913771	-2.502829
51	1	0	-0.304975	-3.993888	-1.089413
52	1	0	0.025888	-2.445558	-0.258762
53	1	0	-0.694023	-3.785494	0.640977
54	1	0	-5.257030	-0.765705	1.920264
55	1	0	-3.563662	-0.803048	2.482644
56	1	0	-4.035324	0.273571	1.142506
57	1	0	-5.923783	-1.809391	-0.186564
58	1	0	-4.788379	-0.784127	-1.105399
59	1	0	-4.750284	-2.557035	-1.288321
60	6	0	-1.755814	2.782058	-0.341577
61	6	0	-3.186056	3.097442	0.102689
62	6	0	-4.063803	2.458146	-1.000744
63	6	0	-3.052769	1.731381	-1.901381
64	1	0	-1.052307	2.580465	0.473208
65	1	0	-1.355320	3.572588	-0.997378
66	1	0	-3.388567	2.635229	1.076338
67	1	0	-3.350965	4.175425	0.199725
68	1	0	-4.783073	1.752016	-0.570599
69	1	0	-4.626325	3.206967	-1.567834

70	1	0	-3.370281	0.734509	-2.225471
71	1	0	-2.799570	2.336518	-2.785183
72	8	0	2.673957	-1.851751	0.172610
73	6	0	3.966021	-1.868097	0.789485
74	6	0	4.970186	-1.727490	-0.364745
75	6	0	4.166550	-2.176230	-1.608025
76	6	0	2.823062	-2.619087	-1.021567
77	1	0	3.997981	-1.057219	1.524933
78	1	0	4.094682	-2.827872	1.313255
79	1	0	5.308523	-0.689929	-0.467785
80	1	0	5.855395	-2.349249	-0.195212
81	1	0	4.023188	-1.340467	-2.303646
82	1	0	4.655402	-2.988179	-2.156403
83	1	0	1.961762	-2.427048	-1.668267
84	1	0	2.840360	-3.688255	-0.755444

Thermochemistry:

Zero-point correction=	0.753488 (Hartree/Particle)
Thermal correction to Energy=	0.793724
Thermal correction to Enthalpy=	0.794668
Thermal correction to Gibbs Free Energy=	0.681997
Sum of electronic and zero-point Energies=	-1312.110146
Sum of electronic and thermal Energies=	-1312.06991
Sum of electronic and thermal Enthalpies=	-1312.068966
Sum of electronic and thermal Free Energies=	-1312.181637

Transition Structure S6$_{TS-B}$

Input:

```
----------------------------------------------------------------------
 #m062x/jul-cc-pvDz opt=(calcfc,noeigen,z-matrix,ts) freq optcyc=50 scrf=(pcm,solvent=THF,read)
 ----------------------------------------------------------------------
```

Optimized Structure:

Center Number	Atomic Number	Atomic Type	Coordinates (Angstroms) X	Y	Z
1	7	0	-0.153072	0.076949	2.542346
2	6	0	-1.396153	0.170671	2.891555
3	6	0	0.620342	1.300233	2.282224
4	6	0	0.629791	-1.081572	2.984759
5	8	0	0.190213	-0.663118	0.429679
6	1	0	-1.913162	-0.712429	3.257481
7	1	0	-1.923595	1.108165	2.734731
8	1	0	1.474506	1.054888	1.642511
9	1	0	-0.026330	2.016000	1.766271
10	1	0	0.929069	1.707181	3.257151
11	1	0	-0.001426	-1.971122	2.933656

12	1	0	1.471820	-1.188665	2.306177
13	1	0	0.944030	-0.890890	4.022412
14	3	0	1.701355	-0.158223	-0.373928
15	3	0	-1.453172	-0.165421	0.227177
16	7	0	-3.239897	-1.341401	0.411102
17	6	0	-2.826814	-2.647073	-0.147697
18	6	0	-4.390317	-0.679331	-0.241597
19	1	0	-3.693984	-3.325773	-0.207573
20	6	0	-2.254728	-2.459828	-1.548684
21	6	0	-1.793575	-3.257833	0.793038
22	1	0	-4.012229	-0.211072	-1.162998
23	6	0	-5.537017	-1.618791	-0.615462
24	6	0	-4.892049	0.420383	0.687302
25	1	0	-1.354161	-1.828904	-1.504516
26	1	0	-1.967875	-3.434441	-1.964830
27	1	0	-2.984920	-2.006305	-2.232056
28	1	0	-1.480304	-4.242056	0.419749
29	1	0	-0.922848	-2.583526	0.852672
30	1	0	-2.225518	-3.401128	1.795478
31	1	0	-6.382584	-1.031668	-0.996530
32	1	0	-5.250721	-2.337661	-1.391983
33	1	0	-5.878790	-2.178297	0.267704
34	1	0	-5.632558	1.048980	0.176756
35	1	0	-5.377464	-0.023548	1.569735
36	1	0	-4.064214	1.054973	1.022380
37	8	0	2.219537	-1.618188	-1.668187
38	7	0	3.044559	1.253094	-0.109804
39	6	0	2.534560	2.593774	-0.214636
40	6	0	4.244515	1.164138	0.685456
41	1	0	2.295013	3.010547	0.806333
42	6	0	3.470005	3.643349	-0.846787
43	6	0	1.218974	2.568378	-0.994661
44	1	0	4.286152	2.002877	1.423284
45	6	0	4.236969	-0.139238	1.495353
46	6	0	5.569155	1.215810	-0.110442
47	1	0	3.756966	3.329288	-1.863271
48	1	0	2.969264	4.622899	-0.909029
49	1	0	4.385105	3.772502	-0.253987
50	1	0	0.760495	3.568317	-1.048379
51	1	0	1.409920	2.224021	-2.024482
52	1	0	0.506002	1.871391	-0.528606
53	1	0	5.194555	-0.321222	2.006997
54	1	0	3.437646	-0.124831	2.247786
55	1	0	4.048656	-0.981354	0.810560
56	1	0	6.442365	1.283049	0.559531
57	1	0	5.673018	0.297581	-0.709411
58	1	0	5.590561	2.067238	-0.799213
59	6	0	1.633773	-2.846132	-1.223409
60	6	0	2.708161	-3.451033	-0.329353
61	6	0	4.005500	-3.086452	-1.072618

62	6	0	3.609691	-1.856181	-1.918330
63	1	0	0.716105	-2.565308	-0.693719
64	1	0	1.425935	-3.493317	-2.093777
65	1	0	2.670665	-2.960013	0.652808
66	1	0	2.587693	-4.530893	-0.185994
67	1	0	4.830297	-2.862635	-0.386052
68	1	0	4.324593	-3.910955	-1.721123
69	1	0	4.138545	-0.938901	-1.637527
70	1	0	3.757791	-2.046026	-2.991891
71	8	0	-2.062872	1.629208	-0.316037
72	6	0	-2.318913	2.921158	0.235699
73	6	0	-3.414107	3.543536	-0.650965
74	6	0	-3.427256	2.647480	-1.910441
75	6	0	-2.179800	1.784563	-1.733991
76	1	0	-2.623594	2.792353	1.280856
77	1	0	-1.391071	3.512366	0.202463
78	1	0	-4.386037	3.531421	-0.146484
79	1	0	-3.171850	4.583814	-0.891139
80	1	0	-4.323597	2.015912	-1.925899
81	1	0	-3.397010	3.223692	-2.840668
82	1	0	-2.244272	0.784440	-2.178361
83	1	0	-1.280118	2.289771	-2.117300
84	1	0	-3.501496	-1.503564	1.384559

Thermochemistry:

Zero-point correction=	0.756359 (Hartree/Particle)
Thermal correction to Energy=	0.797575
Thermal correction to Enthalpy=	0.798519
Thermal correction to Gibbs Free Energy=	0.683009
Sum of electronic and zero-point Energies=	-1312.098836
Sum of electronic and thermal Energies=	-1312.05762
Sum of electronic and thermal Enthalpies=	-1312.056675
Sum of electronic and thermal Free Energies=	-1312.172186

Ground Structure S7

Input:

#m062x/jul-cc-pvDz opt=(calcfc,noeigen,z-matrix) freq optcyc=50 scrf=(pcm,solvent=THF,read)

Optimized Structure:

Center Number	Atomic Number	Atomic Type	Coordinates (Angstroms) X	Y	Z
1	7	0	0.249460	-0.465981	2.469711
2	6	0	-1.101378	0.105600	2.869737
3	6	0	1.505726	0.248567	2.936831

4	6	0	0.313596	-1.869908	2.924484
5	8	0	0.283076	-0.499153	1.045423
6	1	0	-1.203600	-0.134025	3.943225
7	1	0	-0.939568	1.199055	2.829155
8	1	0	1.207960	1.311157	2.982512
9	1	0	1.646699	-0.060270	3.986596
10	1	0	-0.567639	-2.394086	2.544996
11	1	0	1.230423	-2.313779	2.524146
12	1	0	0.324816	-1.891459	4.019606
13	3	0	2.068188	0.120808	0.920427
14	3	0	-1.584946	-0.338257	0.858138
15	7	0	-2.765632	-1.929661	0.071805
16	6	0	-2.011169	-2.761412	-0.884148
17	6	0	-4.032718	-1.359621	-0.427992
18	1	0	-2.671641	-3.512117	-1.349389
19	6	0	-1.427827	-1.881284	-1.983060
20	6	0	-0.914363	-3.501641	-0.130549
21	1	0	-3.761876	-0.575609	-1.147964
22	6	0	-4.947205	-2.363799	-1.130667
23	6	0	-4.754588	-0.705913	0.744531
24	1	0	-0.796057	-1.098775	-1.535857
25	1	0	-0.805769	-2.482888	-2.658290
26	1	0	-2.213585	-1.406684	-2.585229
27	1	0	-0.352317	-4.144519	-0.820447
28	1	0	-0.228603	-2.780588	0.331399
29	1	0	-1.347241	-4.140150	0.653112
30	1	0	-5.910098	-1.888671	-1.359498
31	1	0	-4.522316	-2.726592	-2.074090
32	1	0	-5.138738	-3.229065	-0.478868
33	1	0	-5.634898	-0.152462	0.392997
34	1	0	-5.099285	-1.475206	1.451843
35	1	0	-4.090985	-0.019491	1.284485
36	8	0	1.853776	1.790994	-0.093402
37	7	0	3.766630	-0.768074	-0.006782
38	6	0	3.536402	-1.566471	-1.226518
39	6	0	4.980433	0.076066	-0.016844
40	1	0	3.359435	-0.852850	-2.043803
41	6	0	4.716157	-2.459404	-1.609935
42	6	0	2.264126	-2.379074	-1.030982
43	1	0	5.881187	-0.557095	-0.102615
44	6	0	4.966001	1.047995	-1.191199
45	6	0	5.058356	0.831269	1.304181
46	1	0	4.963587	-3.138851	-0.780433
47	1	0	4.457982	-3.069422	-2.485239
48	1	0	5.611537	-1.876677	-1.858949
49	1	0	2.019107	-2.938121	-1.943633
50	1	0	2.394347	-3.106075	-0.213447
51	1	0	1.419987	-1.729877	-0.767356
52	1	0	5.831851	1.717815	-1.114329
53	1	0	5.029936	0.534557	-2.158542

54	1	0	4.052286	1.656676	-1.163423
55	1	0	5.999798	1.392539	1.362522
56	1	0	4.218278	1.534359	1.390958
57	1	0	5.012397	0.141887	2.157531
58	6	0	1.249336	1.491741	-1.352121
59	6	0	0.922477	2.867771	-1.931721
60	6	0	0.599980	3.702628	-0.669318
61	6	0	0.989115	2.770708	0.493255
62	1	0	1.963040	0.904773	-1.940877
63	1	0	0.335897	0.900581	-1.169781
64	1	0	1.802038	3.272314	-2.445739
65	1	0	0.097320	2.830183	-2.651397
66	1	0	1.185579	4.627547	-0.650607
67	1	0	-0.459438	3.978958	-0.612077
68	1	0	1.543345	3.262118	1.298900
69	1	0	0.112330	2.248603	0.907606
70	8	0	-2.235297	1.341647	-0.034078
71	6	0	-2.798016	2.349854	0.815250
72	6	0	-4.102500	2.794609	0.130352
73	6	0	-3.977855	2.248928	-1.310314
74	6	0	-2.533904	1.748803	-1.367901
75	1	0	-2.928037	1.903450	1.804913
76	1	0	-2.084790	3.185931	0.892595
77	1	0	-4.976725	2.373144	0.637436
78	1	0	-4.197632	3.885354	0.147559
79	1	0	-4.676334	1.419711	-1.473013
80	1	0	-4.172949	3.011737	-2.071229
81	1	0	-2.368102	0.893594	-2.032039
82	1	0	-1.852847	2.563943	-1.658603
83	1	0	-2.990261	-2.510522	0.881363
84	1	0	3.873683	-1.429686	0.766802

Thermochemistry:

Zero-point correction=	0.758916 (Hartree/Particle)
Thermal correction to Energy=	0.799804
Thermal correction to Enthalpy=	0.800748
Thermal correction to Gibbs Free Energy=	0.687204
Sum of electronic and zero-point Energies=	-1312.121151
Sum of electronic and thermal Energies=	-1312.080263
Sum of electronic and thermal Enthalpies=	-1312.079319
Sum of electronic and thermal Free Energies=	-1312.192863

Transition Structure S7$_{TS}$

Input:

Center Number	Atomic Number	Atomic Type	Coordinates (Angstroms) X	Y	Z
1	7	0	0.291324	-0.973473	2.385659
2	6	0	-0.929967	-0.216765	2.670801
3	6	0	1.483928	-0.543895	2.857489
4	6	0	0.095604	-2.429318	2.442601
5	8	0	0.334743	-1.005365	0.457158
6	1	0	-1.524348	-0.839297	3.359164
7	1	0	-0.627466	0.696535	3.202409
8	1	0	1.591936	0.516532	3.065120
9	1	0	2.355517	-1.164479	2.654781
10	1	0	-0.798860	-2.679802	1.869856
11	1	0	0.964421	-2.924548	2.003218
12	1	0	-0.018112	-2.709270	3.499874
13	3	0	1.900283	-0.182039	0.583156
14	3	0	-1.339980	-0.382817	0.585135
15	7	0	-2.955581	-1.779634	0.060929
16	6	0	-2.590316	-2.428629	-1.208967
17	6	0	-4.217023	-1.015357	0.082630
18	1	0	-3.440586	-3.007012	-1.610868
19	6	0	-2.197084	-1.368369	-2.229911
20	6	0	-1.434173	-3.386020	-0.942704
21	1	0	-4.002855	-0.047792	-0.391432
22	6	0	-5.383472	-1.665023	-0.662191
23	6	0	-4.583590	-0.768726	1.542624
24	1	0	-1.307059	-0.831406	-1.869978
25	1	0	-1.957575	-1.838924	-3.192470
26	1	0	-3.008177	-0.646263	-2.397629
27	1	0	-1.116753	-3.862799	-1.879845
28	1	0	-0.597593	-2.820541	-0.502465
29	1	0	-1.746950	-4.181856	-0.250042
30	1	0	-6.294635	-1.073063	-0.501830
31	1	0	-5.207442	-1.722444	-1.743399
32	1	0	-5.565120	-2.682153	-0.284749
33	1	0	-5.392523	-0.031182	1.622469
34	1	0	-4.934342	-1.704823	2.003905
35	1	0	-3.707980	-0.415403	2.102221
36	8	0	1.933370	1.752859	0.260263
37	7	0	3.753504	-0.931717	-0.130801
38	6	0	3.663592	-1.369371	-1.542029
39	6	0	4.963387	-0.164131	0.229888
40	1	0	3.452393	-0.469095	-2.138509
41	6	0	4.942559	-2.018762	-2.071463
42	6	0	2.476831	-2.318046	-1.672855
43	1	0	5.865282	-0.777607	0.059322
44	6	0	5.083743	1.106077	-0.603844
45	6	0	4.895815	0.174380	1.714240

46	1	0	5.225051	-2.871218	-1.435476
47	1	0	4.772382	-2.395250	-3.088497
48	1	0	5.787256	-1.320033	-2.108127
49	1	0	2.283494	-2.538232	-2.730733
50	1	0	2.704784	-3.271039	-1.168418
51	1	0	1.578055	-1.892469	-1.201438
52	1	0	5.985623	1.652191	-0.298937
53	1	0	5.171370	0.893107	-1.676418
54	1	0	4.212965	1.751708	-0.433963
55	1	0	5.813288	0.687823	2.028536
56	1	0	4.037834	0.832455	1.914126
57	1	0	4.788916	-0.733846	2.323172
58	6	0	1.534820	1.946420	-1.099958
59	6	0	1.280132	3.447805	-1.190152
60	6	0	0.702356	3.779703	0.202766
61	6	0	1.012682	2.526627	1.045929
62	1	0	2.346065	1.584384	-1.741706
63	1	0	0.620958	1.361995	-1.291886
64	1	0	2.228995	3.973608	-1.349291
65	1	0	0.603642	3.707815	-2.011954
66	1	0	1.170304	4.675525	0.623603
67	1	0	-0.378178	3.956608	0.161031
68	1	0	1.507740	2.750001	1.996992
69	1	0	0.111295	1.922234	1.228038
70	8	0	-1.972799	1.431995	-0.226535
71	6	0	-2.783929	2.191384	0.676041
72	6	0	-4.017754	2.634734	-0.134288
73	6	0	-3.610501	2.381795	-1.603921
74	6	0	-2.119364	2.060286	-1.497787
75	1	0	-3.000576	1.550809	1.535049
76	1	0	-2.208045	3.063290	1.025681
77	1	0	-4.904309	2.051354	0.138750
78	1	0	-4.243633	3.690383	0.049514
79	1	0	-4.154240	1.524325	-2.019317
80	1	0	-3.796610	3.245847	-2.250454
81	1	0	-1.745565	1.375597	-2.264892
82	1	0	-1.521474	2.986567	-1.517190
83	1	0	-3.018576	-2.504104	0.776372
84	1	0	3.762202	-1.783102	0.436613

Thermochemistry:

Zero-point correction=	0.757180 (Hartree/Particle)
Thermal correction to Energy=	0.798094
Thermal correction to Enthalpy=	0.799038
Thermal correction to Gibbs Free Energy=	0.684748
Sum of electronic and zero-point Energies=	-1312.091145
Sum of electronic and thermal Energies=	-1312.050232
Sum of electronic and thermal Enthalpies=	-1312.049288

Sum of electronic and thermal Free Energies= -1312.163577

Ground Structure S8

Input:

#m062x/jul-cc-pvDz opt=(calcfc,noeigen) freq optcyc=50 scrf=(pcm,solvent=THF,read)

Optimized Structure:

Center Number	Atomic Number	Atomic Type	Coordinates (Angstroms) X	Y	Z
1	7	0	0.273505	-0.878888	2.739632
2	6	0	-1.050443	-0.680686	2.813816
3	6	0	1.231976	-0.023028	3.061096
4	6	0	0.697133	-2.118566	2.062679
5	8	0	0.339099	-0.715294	-0.529131
6	1	0	-1.694332	-1.539080	2.671501
7	1	0	-1.405908	0.231366	3.283647
8	1	0	0.959433	0.942271	3.475772
9	1	0	2.259327	-0.298401	2.866628
10	1	0	0.668635	-1.856496	0.971355
11	1	0	1.698001	-2.385919	2.415918
12	1	0	-0.009250	-2.914673	2.319051
13	3	0	1.846431	0.041838	-0.174715
14	3	0	-1.253430	-0.512398	0.144965
15	7	0	-2.881267	-1.959824	-0.101194
16	6	0	-2.586816	-2.718318	-1.333506
17	6	0	-4.212216	-1.325014	-0.026892
18	1	0	-3.386863	-3.454020	-1.527909
19	6	0	-2.497517	-1.767313	-2.520992
20	6	0	-1.273229	-3.465023	-1.130175
21	1	0	-4.169390	-0.414775	-0.644033
22	6	0	-5.365535	-2.198724	-0.522279
23	6	0	-4.449266	-0.903661	1.417628
24	1	0	-1.683513	-1.048536	-2.347521
25	1	0	-2.275673	-2.334962	-3.434297
26	1	0	-3.440465	-1.226845	-2.683556
27	1	0	-1.039336	-4.062177	-2.022119
28	1	0	-0.475095	-2.725202	-0.945425
29	1	0	-1.352461	-4.154500	-0.275304
30	1	0	-6.320342	-1.691432	-0.330986
31	1	0	-5.302710	-2.402167	-1.597872
32	1	0	-5.375619	-3.160121	0.012578
33	1	0	-5.351850	-0.285231	1.499718
34	1	0	-4.589034	-1.791963	2.053368
35	1	0	-3.590387	-0.338512	1.794495
36	8	0	1.925105	2.069613	-0.385202
37	7	0	3.765446	-0.836784	0.135154

38	6	0	3.935742	-1.839176	-0.943229
39	6	0	4.884203	0.106757	0.320249
40	1	0	3.799691	-1.302592	-1.895000
41	6	0	5.307813	-2.514137	-0.951764
42	6	0	2.817522	-2.868049	-0.816780
43	1	0	5.820452	-0.443531	0.518355
44	6	0	5.081528	0.956195	-0.929320
45	6	0	4.591717	0.980061	1.534214
46	1	0	5.506921	-2.984310	0.023318
47	1	0	5.325939	-3.299695	-1.718152
48	1	0	6.122816	-1.813295	-1.170637
49	1	0	2.861412	-3.573297	-1.657216
50	1	0	2.946375	-3.446285	0.113100
51	1	0	1.838382	-2.361265	-0.801920
52	1	0	5.894509	1.674031	-0.760829
53	1	0	5.347513	0.347781	-1.802349
54	1	0	4.163808	1.516228	-1.149452
55	1	0	5.420125	1.679146	1.704958
56	1	0	3.670462	1.556861	1.374767
57	1	0	4.476682	0.367747	2.439408
58	6	0	1.461680	2.290675	-1.725243
59	6	0	0.858786	3.700503	-1.711371
60	6	0	0.473862	3.917939	-0.228609
61	6	0	0.895391	2.609750	0.453661
62	1	0	2.319477	2.179026	-2.397856
63	1	0	0.711274	1.517622	-1.948397
64	1	0	1.596152	4.443758	-2.033381
65	1	0	-0.003484	3.770937	-2.385043
66	1	0	1.017729	4.770403	0.192488
67	1	0	-0.597948	4.108250	-0.098717
68	1	0	1.319702	2.743798	1.454764
69	1	0	0.067537	1.883312	0.490890
70	8	0	-2.291156	1.240423	0.161430
71	6	0	-2.711345	2.183794	1.146892
72	6	0	-3.858917	2.927331	0.480646
73	6	0	-3.339699	3.074136	-0.956034
74	6	0	-2.442807	1.836765	-1.138834
75	1	0	-2.994712	1.630402	2.047330
76	1	0	-1.881293	2.865454	1.397323
77	1	0	-4.762141	2.303743	0.506937
78	1	0	-4.079048	3.887900	0.958263
79	1	0	-4.145779	3.107632	-1.695847
80	1	0	-2.751944	3.994839	-1.057848
81	1	0	-2.889952	1.079192	-1.793576
82	1	0	-1.449793	2.091640	-1.533954
83	1	0	-2.816093	-2.617426	0.677564
84	1	0	3.657508	-1.357685	1.007896

Thermochemistry:

Zero-point correction=	0.757290 (Hartree/Particle)
Thermal correction to Energy=	0.799567
Thermal correction to Enthalpy=	0.800511
Thermal correction to Gibbs Free Energy=	0.682637
Sum of electronic and zero-point Energies=	-1312.143125
Sum of electronic and thermal Energies=	-1312.100849
Sum of electronic and thermal Enthalpies=	-1312.099905
Sum of electronic and thermal Free Energies=	-1312.217779

References

(1) Love, B. E.; Jones, E. G. The Use of Salicylaldehyde Phenylhydrazone as an Indicator for the Titration of Organometallic Reagents. *J. Org. Chem.* **1999**, *64* (10), 3755-3756. DOI: 10.1021/jo982433e.
(2) Neal, M. J.; Hejnosz, S. L.; Rohde, J. J.; Evanseck, J. D.; Montgomery, T. D. Multi-Ion Bridged Pathway of N-Oxides to 1,3-Dipole Dilithium Oxide Complexes. *J. Org. Chem.* **2021**, *86* (17), 11502-11518. DOI: 10.1021/acs.joc.1c01047.
(3) Algera, R. F.; Gupta, L.; Hoepker, A. C.; Liang, J.; Ma, Y.; Singh, K. J.; Collum, D. B. Lithium Diisopropylamide: Nonequilibrium Kinetics and Lessons Learned about Rate Limitation. *J. Org. Chem.* **2017**, *82* (9), 4513-4532. DOI: 10.1021/acs.joc.6b03083 From NLM Medline.
(4) Mack, K. A.; Collum, D. B. Case for Lithium Tetramethylpiperidide-Mediated Ortholithiations: Reactivity and Mechanisms. *J. Am. Chem. Soc.* **2018**, *140* (14), 4877-4883. DOI: 10.1021/jacs.8b00590.
(5) Collum, D. B.; McNeil, A. J.; Ramirez, A. Lithium diisopropylamide: solution kinetics and implications for organic synthesis. *Angew Chem Int Ed Engl* **2007**, *46* (17), 3002-3017. DOI: 10.1002/anie.200603038 From NLM Medline.
(6) Gennari, C.; Vulpetti, A.; Pain, G. Highly enantio- and diastereoselective boron aldol reactions of α-heterosubstituted thioacetates with aldehydes and silyl imines. *Tetrahedron* **1997**, *53* (16), 5909-5924. DOI: https://doi.org/10.1016/S0040-4020(97)00251-2.
(7) Georg, G. I.; Harriman, G. C. B.; Hepperle, M.; Clowers, J. S.; Vander Velde, D. G.; Himes, R. H. Synthesis, Conformational Analysis, and Biological Evaluation of Heteroaromatic Taxanes. *J. Org. Chem.* **1996**, *61* (8), 2664-2676. DOI: 10.1021/jo951961c.
(8) Borlinghaus, N.; Gergel, S.; Nestl, B. M. Biocatalytic Access to Piperazines from Diamines and Dicarbonyls. *ACS Catalysis* **2018**, *8* (4), 3727-3732. DOI: 10.1021/acscatal.8b00291.
(9) Chen, J.; Brown, D. P.; Wang, Y. J.; Chen, Z. S. New phenstatin-fatty acid conjugates: synthesis and evaluation. *Bioorg Med Chem Lett* **2013**, *23* (18), 5119-5122. DOI: 10.1016/j.bmcl.2013.07.025 From NLM Medline.
(10) Xu, L.-W.; Li, L.; Bai, X.-F.; Song, T.; Deng, W.-H.; Wei, Y.-L.; Xia, C.-G. (EtO)3SiH-Promoted Palladium-Catalyzed Isomerization of Olefins: Convenient Synthesis of Internal Alkenes from Terminal Alkenes. *Synlett* **2013**, *25* (03), 417-422. DOI: 10.1055/s-0033-1340290.
(11) Kwong, C. K.-W.; Fu, M. Y.; Law, H. C.-H.; Toy, P. H. Isomerization of Electron-Poor Alkynes to the Corresponding (E,E)-1,3-Dienes Using a Bifunctional Polymeric Catalyst Bearing Triphenylphosphine and Phenol Groups. *Synlett* **2010**, *2010* (17), 2617-2620. DOI: 10.1055/s-0030-1258576.
(12) Lewis, R. S.; Wisthoff, M. F.; Grissmerson, J.; Chain, W. J. Metal-Free Functionalization of N,N-Dialkylanilines via Temporary Oxidation to N,N-Dialkylaniline N-Oxides and Group Transfer. *Organic Letters* **2014**, *16* (14), 3832-3835. DOI: 10.1021/ol501813s.
(13) Karaali, N.; Aydin, S.; Baltas, N.; Mentese, E. Synthesis of novel tetra-substituted benzimidazole compounds containing certain heterostructures with antioxidant and anti-urease activities. *Journal of Heterocyclic Chemistry* **2020**, *57* (4), 1806-1815. DOI: https://doi.org/10.1002/jhet.3905.
(14) Verma, P.; Truhlar, D. G. Status and Challenges of Density Functional Theory. *Trends Chem.* **2020**, *2* (4), 302-318.
(15) Wilson, S. Many-body perturbation theory and its application to the molecular structure problem. *Chemical Modelling* **2008**, *5*, 208-248.
(16) Zhao, Y.; Truhlar, D. G. The M06 suite of density functionals for main group thermochemistry, thermochemical kinetics, noncovalent interactions, excited states, and transition elements: two new functionals and systematic testing of four M06-class functionals and 12 other functionals. *Theor. Chem. Acc.* **2008**, *120* (1-3), 215-241.
(17) Dunning, T. H., Jr. Gaussian basis sets for use in correlated molecular calculations. I. The atoms boron through neon and hydrogen. *J. Chem. Phys.* **1989**, *90* (2), 1007-1023.

(18) Neal, M. J.; Hejnosz, S. L.; Rohde, J. J.; Evanseck, J. D.; Montgomery, T. D. Multi-Ion Bridged Pathway of N-Oxides to 1,3-Dipole Dilithium Oxide Complexes. *J. Org. Chem.* **2021**, *86* (17), 11502-11518.
