# Supplementary material for: [3 + 2] Cycloadditions of Tertiary Amine N-Oxides and Silyl Imines as an Innovative Route to 1,2-Diamines
Source: Org Lett. 2023 Jun 15;25(25):4638–43. doi: 10.1021/acs.orglett.3c01396 (PMC10325142; doi:10.1021/acs.orglett.3c01396)
Supplement: Supplementary file 1 — ol3c01396_si_001.zip [file ol3c01396_si_001.zip › 4f/4f_1HNMR/pdata/1/Exp.html]

Experimentals report

^1^H NMR (400 MHz, C_6_D_6_) δ 7.33 (d, *J* = 4.4 Hz, 1H), 7.26 (t, *J* = 8.3 Hz, 1H), 4.05 (d, *J* = 7.8 Hz, 1H), 4.03 (d, *J* = 7.8 Hz, 1H), 2.78 (d, *J* = 5.3 Hz, 1H), 2.74 (d, *J* = 14.1 Hz, 1H), 2.70 (d, *J* = 7.9 Hz, 1H).
