# Supplementary material for: [3 + 2] Cycloadditions of Tertiary Amine N-Oxides and Silyl Imines as an Innovative Route to 1,2-Diamines
Source: Org Lett. 2023 Jun 15;25(25):4638–43. doi: 10.1021/acs.orglett.3c01396 (PMC10325142; doi:10.1021/acs.orglett.3c01396)
Supplement: Supplementary file 1 — ol3c01396_si_001.zip [file ol3c01396_si_001.zip › 4q/4q_1HNMR/pdata/1/Exp.html]

Experimentals report

^1^H NMR (400 MHz, CDCl_3_) δ 8.54 (d, *J* = 0.8 Hz, 1H), 8.53 (d, *J* = 1.6 Hz, 1H), 7.70 (dt, *J* = 1.8, 7.7 Hz, 1H), 7.34 (d, *J* = 7.8 Hz, 1H), 7.23 (d, *J* = 8.6 Hz, 1H), 7.23 (d, *J* = 1.6 Hz, 1H), 7.23 (d, *J* = 12.4 Hz, 1H), 7.22 (d, *J* = 7.5 Hz, 1H), 4.34 (d, *J* = 9.3 Hz, 1H), 4.33 (d, *J* = 9.3 Hz, 1H), 3.25 (d, *J* = 4.4 Hz, 1H), 3.22 (d, *J* = 4.4 Hz, 1H), 3.12 (d, *J* = 9.3 Hz, 1H), 3.09 (d, *J* = 9.4 Hz, 1H).
